# Clonal hematopoiesis driven by mutated DNMT3A promotes inflammatory bone loss

**DOI:** 10.1016/j.cell.2024.05.003

**Published:** 2024-06-04

**Authors:** Hui Wang, Kimon Divaris, Bohu Pan, Xiaofei Li, Jong-Hyung Lim, Gundappa Saha, Marko Barovic, Danai Giannakou, Jonathan M. Korostoff, Yu Bing, Souvik Sen, Kevin Moss, Di Wu, James D. Beck, Christie M. Ballantyne, Pradeep Natarajan, Kari E. North, Mihai G. Netea, Triantafyllos Chavakis, George Hajishengallis

**Affiliations:** 1 Department of Basic and Translational Sciences, Laboratory of Innate Immunity and Inflammation, Penn Dental Medicine, University of Pennsylvania, Philadelphia, PA 19104, USA; 2 Division of Pediatric and Public Health, Adams School of Dentistry, University of North Carolina, Chapel Hill, Chapel Hill, NC 27599, USA; 3 Department of Epidemiology, Gillings School of Global Public Health, University of North Carolina, Chapel Hill, Chapel Hill, NC 27599, USA; 4 Division of Bioinformatics and Biostatistics, National Center for Toxicological Research, US Food and Drug Administration, Jefferson, AR 72079, USA; 5 Shanghai Jiao Tong University, School of Life Sciences and Biotechnology, Sheng Yushou Center of Cell Biology and Immunology, Shanghai 200240, China; 6 Institute for Clinical Chemistry and Laboratory Medicine, University Hospital, Technische Universität Dresden, 01307 Dresden, Germany; 7 Department of Periodontics, Laboratory of Innate Immunity and Inflammation, Penn Dental Medicine, University of Pennsylvania, Philadelphia, PA 19104, USA; 8 Human Genetics Center, Department of Epidemiology, School of Public Health, The University of Texas Health Science Center at Houston, Houston, TX 77030, USA; 9 Department of Neurology, University of South Carolina, Columbia, SC 29209, USA; 10 Center for the Study of Aphasia Recovery, University of South Carolina, Columbia, SC 29209, USA; 11 Department of Biostatistics and Health Data Sciences, School of Medicine, Indiana University, Indianapolis, IN 46202, USA; 12 Division of Oral and Craniofacial Health Sciences, Adams School of Dentistry, University of North Carolina, Chapel Hill, Chapel Hill, NC 27599, USA; 13 Department of Biostatistics, Gillings School of Global Public Health, University of North Carolina, Chapel Hill, Chapel Hill, NC 27599, USA; 14 Division of Comprehensive Oral Health-Periodontology, Adams School of Dentistry, University of North Carolina, Chapel Hill, Chapel Hill, NC 27599, USA; 15 Department of Medicine, Baylor College of Medicine, Houston, TX 77030, USA; 16 Cardiovascular Research Center and Center for Genomic Medicine, Massachusetts General Hospital, Boston, MA 02114, USA; 17 Program in Medical and Population Genetics, Broad Institute of Harvard and MIT, Cambridge, MA 02141, USA; 18 Department of Medicine, Harvard Medical School, Boston, MA 02115, USA; 19 Department of Internal Medicine and Radboud Center for Infectious Diseases, Radboud University Medical Center, 6525 XZ Nijmegen, the Netherlands; 20 Department of Immunology and Metabolism, LIMES, University of Bonn, 53115 Bonn, Germany; 21 These authors contributed equally; 22 Senior author; 23 Lead contact

## Abstract

Clonal hematopoiesis of indeterminate potential (CHIP) arises from aging-associated acquired mutations in hematopoietic progenitors, which display clonal expansion and produce phenotypically altered leukocytes. We associated CHIP-*DNMT3A* mutations with a higher prevalence of periodontitis and gingival inflammation among 4,946 community-dwelling adults. To model DNMT3A-driven CHIP, we used mice with the heterozygous loss-of-function mutation R878H, equivalent to the human hotspot mutation R882H. Partial transplantation with *Dnmt3a*^R878H/+^ bone marrow (BM) cells resulted in clonal expansion of mutant cells into both myeloid and lymphoid lineages and an elevated abundance of osteoclast precursors in the BM and osteoclastogenic macrophages in the periphery. DNMT3A-driven clonal hematopoiesis in recipient mice promoted naturally occurring periodontitis and aggravated experimentally induced periodontitis and arthritis, associated with enhanced osteoclastogenesis, IL-17-dependent inflammation and neutrophil responses, and impaired regulatory T cell immunosuppressive activity. DNMT3A-driven clonal hematopoiesis and, subsequently, periodontitis were suppressed by rapamycin treatment. DNMT3A-driven CHIP represents a treatable state of maladaptive hematopoiesis promoting inflammatory bone loss.

## In brief

DNMT3A mutations associated with clonal hematopoiesis result in elevated osteoclast precursors in the bone marrow (BM) and osteoclastogenic macrophages in the periphery, along with aggravated neutrophilic inflammation and impaired T-regulatory activity, which collectively promote periodontitis and arthritis.

## Graphical abstract

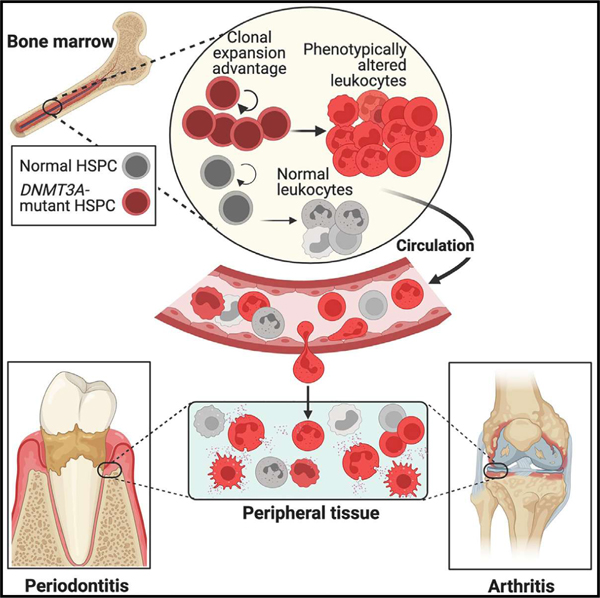

## INTRODUCTION

With advancing age, hematopoietic stem and progenitor cells (HSPCs) in the bone marrow (BM) progressively acquire somatic mutations, some of which may confer a proliferative advantage to the mutantcell, enabling disproportionate clonal expansion relative to normal clones. This fitness advantage leads to the generation of genetically distinct progeny, which constitute an outsized fraction of blood leukocytes and often have altered phenotype compared with normal leukocytes.^1–3^ In the absence of apparent hematologic disease, this condition is designated clonal hematopoiesis of indeterminate potential (CHIP). With a variant allele fraction (VAF) threshold set at 2%, CHIP-mutant clones are rare in young adults (<40 years) but increase with age and are detectable in >10% of individuals older than 65.^1,4–7^ CHIP development may also be driven by selective pressure from cytotoxic chemotherapy or smoking.^8^ CHIP carriers have increased risk of not only hematologic malignancies but also of atherosclerotic cardiovascular disease, heart failure, and inflammation-related disorders, such as chronic liver or kidney disease.^4,5,9–14^ CHIP mutations typically cause loss of function and occur in several genes, yet predominantly in three genes encoding epigenetic regulators: DNA methyl-transferase-3A (*DNMT3A*), the most frequently mutated gene in CHIP, ten-eleven translocation methylcytosine dioxygenase-2 (*TET2*), and associated sex combs-like-1 (*ASXL1*).^4–7^ The selective clonal expansion advantage in CHIP may be accompanied by a modest bias toward myelopoiesis in case of *TET2* mutations, whereas no specific hematopoietic lineage is preferentially affected by DNMT3A mutations.^15–17^ Similarly, in DNMT3A-driven CHIP in mice, *Dnmt3a*-mutant HSPCs expand without apparent bias toward the production of particular types of mature hematopoietic cells.^18^ This model is based on the use of mice harboring a heterozygous mutation (R878H) that causes dominant negative loss of DNMT3A methylation activity^18^ and is equivalent to the human hotspot heterozygous mutation R882H.^19^

Since chronic inflammatory diseases are largely driven by inflammatory immune cells, CHIP-driven alterations to their precursors in the BM are likely to influence multiple disorders. Periodontitis, a prevalent inflammatory disease of the tooth-supporting tissues, remains a significant public health burden^20–23^ and is epidemiologically associated with rheumatoid arthritis,^24–26^ which leads to progressive erosion of cartilage and bone in the joints.^27^ An individual’s susceptibility to periodontitis, arthritis, and chronic diseases in general increases with aging.^28–32^ We reasoned that CHIP may not merely correlate with aging^1,6,7^ but may also contribute causally to inflammatory bone loss disorders, such as periodontitis and arthritis. This hypothesis was tested both at an observational/epidemiological and a preclinical/experimental level in the context of CHIP driven by mutations in *DNMT3A*, the most frequently mutated gene in CHIP.^4–6^

## RESULTS

### Higher periodontitis prevalence in CHIP individuals with *DNMT3A* mutations

In a community-based cohort of 4,946 adult participants of the Atherosclerosis Risk in Communities (ARIC) study^33,34^ ages 52–74 (mean age 62 years), we identified 3.9% of individuals (*n* = 191) as CHIP carriers. Similar to published literature,^4–6^ the most common mutations involved *DNMT3A* (61.8% of total carriers; *n* = 118), followed by *TET2* and *ASXL1* (Table S1). The majority of CHIP carriers (*n* = 108, 2.2% of sample, and 56.5% of total carriers) exhibited VAF > 10% (Table S1). CHIP increased with age and was more common among current (5.7%) vs. ever smokers (3.6%) (Table S2). CHIP due to *DNMT3A* mutations was significantly associated with a diagnosis of severe periodontitis (stage IV vs. stages I–III), as well as quantitative measures of “clinical attachment loss” and “gingival inflammation” (Table 1). The observed differences in periodontitis and clinical measures of periodontal disease were robust to adjustments for participants’ age and sex. For example, when adjusted for age and sex, the prevalence of stage IV periodontitis in carriers with *DNMT3A* mutations was (average marginal effect [AME]) 11 percentage points (95% confidence interval [CI] = 0.0–21.5) higher than in those without CHIP (Table S3). In conclusion, *DNMT3A* CHIP is associated with higher prevalence and severity of periodontitis.

### Clonal expansion of *Dnmt3a*^R878H/+^ cells promotes naturally occurring periodontal inflammation and bone loss

**Figure 1.**
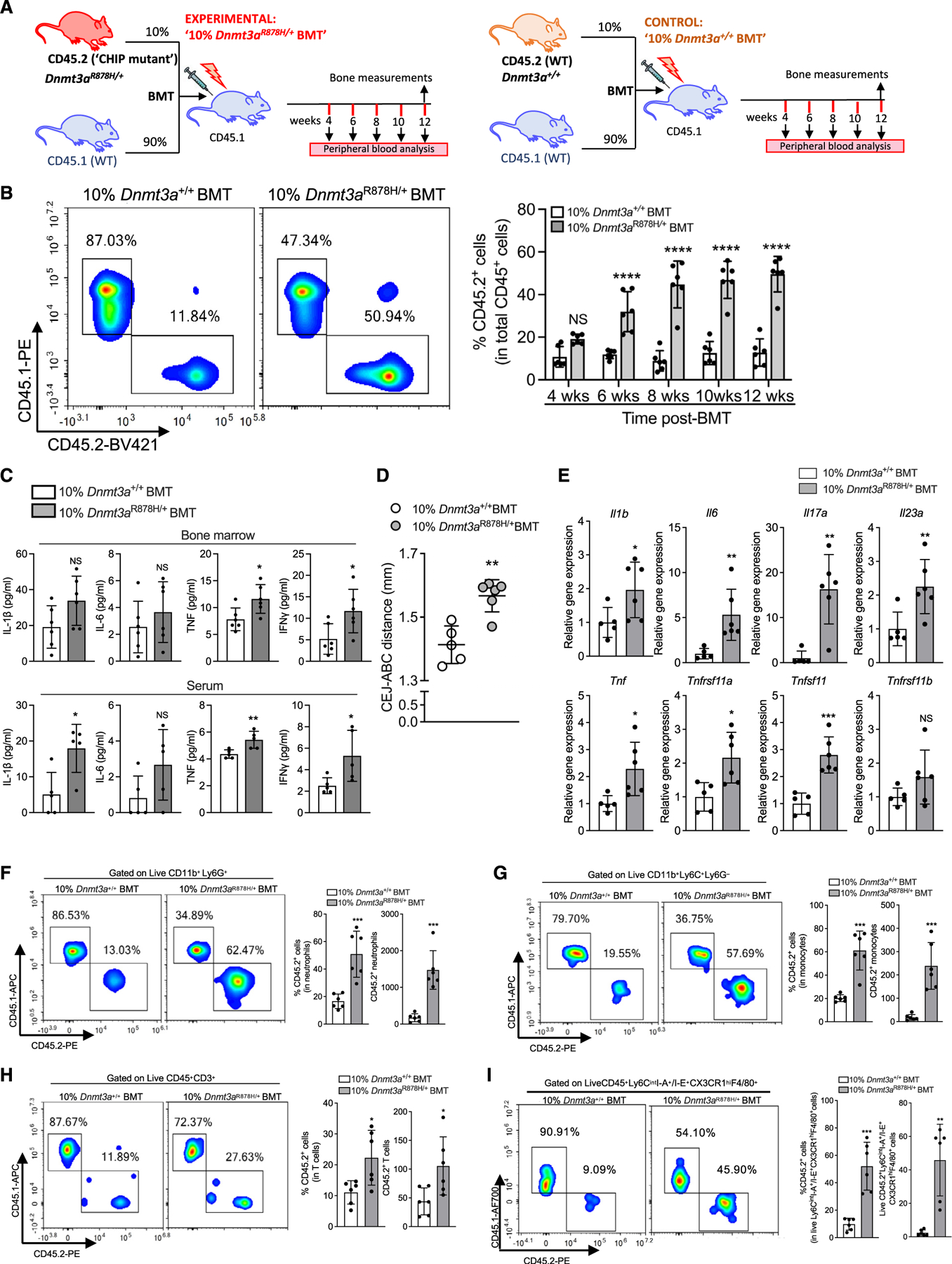
Clonal expansion of *Dnmt3a*^R878H/+^ cells after BMT promotes systemic inflammation and naturally occurring periodontitis (A) Lethally irradiated CD45.1 mice were transplanted with 10% *Dnmt3a*^R878H/+^ CD45.2^+^ BM cells and 90% WT CD45.1^+^ BM cells (10%*Dnmt3a*^R878H/+^BMT group) or 10%*Dnmt3a*^*+/+*^ CD45.2^+^ BM cells and 90% WT CD45.1^+^ cells (10%*Dnmt3a*^+/+^BMT group). (B) Representative FACS plots (12 weeks post-BMT) and percentage of CD45.2^+^ white blood cells in peripheral blood at indicated time intervals. (C–H) All analyses were performed 12 weeks post-BMT. (C) Cytokine levels in the BM extracellular fluid (top) and serum (bottom) 12 weeks post-BMT. (D) Periodontal bone heights (CEJ-ABC distance) 12 weeks post-BMT. (E) Relative gingival mRNA expression of indicated molecules. (F–H) Representative FACS plots (left), percentage (middle), and absolute numbers (right) of CD45.2^+^ neutrophils (live CD11b^+^Ly6G^+^) (F), CD45.2^+^ monocytes (live CD11b^+^Ly6C^+^Ly6G^−^) (G), and CD45.2^+^ T cells (live CD3^+^) (H). (I) Representative FACS plots (left), percentage (middle), and absolute numbers (right) of CD45.2^+^ osteoclastogenic macrophages (Ly6C^int^I-A^+^/I-E^+^CX3CR1^hi^ F4/80^+^). Data are means ± SD (*n* = 5–6 mice/group). *p < 0.05, **p < 0.01, ***p < 0.001, ****p < 0.0001. NS, not significant. Two-way ANOVA with repeated measures and Sidak’s post-test (B); Student’s unpaired t test (C–I) except for (C bottom; IL-1β and IL-6), (E; *Il17a* and *Il23a*), and (I right) (Mann-Whitney U test). See also Figure S1.

Loberg et al. established a DNMT3A-driven CHIP model in mice by transplanting *Dnmt3a*^R878H/+^ BM cells at a 50:50 ratio with wild-type (WT) CD45.1 BM cells and observed significant expansion of *Dnmt3a*^R878H/+^ HSPCs relative to *Dnmt3a*^*+/+*^ controls.^18^ Another BM transplantation (BMT) study, in which DNMT3A-driven CHIP was modeled with *Dnmt3a*^−*/*−^ BM cells and linked CHIP to osteoporosis, involved the use of non-competitive BMT (100%*Dnmt3a*^−*/*−^ BM cells).^36^ Since R50% is not a typical VAF in human CHIP,^4,37^ we transplanted a clinically relevant fraction (10%) of mutant BM cells. Specifically, lethally irradiated CD45.1^+^ mice were subjected to competitive BMT with 10% BM cells from CD45.2^+^ *Dnmt3a*^R878H/+^ mice (or CD45.2^+^ *Dnmt3a*^*+/+*^ controls) and 90% WT BM cells from CD45.1^+^ mice. Experimental (designated “10%*Dnmt3a*^R878H/+^ BMT” mice) and control (“10%*Dnmt3a*^+/+^BMT” mice) recipients were sampled for peripheral blood analysis from 4 to 12 weeks post-BMT (Figure 1A). CD45.2^+^ blood cells expanded significantly over time in 10%*Dnmt3a*^R878H/+^BMT mice relative to 10%*Dnmt3a*^+/+^BMT controls, in which donor-derived CD45.2^+^ (*Dnmt3a*^*+/+*^) cells did not expand (Figure 1B). The donor *Dnmt3a*^R878H/+^ hematopoietic cells produced myeloid (CD11b^+^), B cell (CD19^+^), and T cell (CD3^+^) lineages (Figure S1A) without affecting the total white blood cell counts in 10%*Dnmt3a*^R878H/+^BMT mice, compared with controls (Figure S1B), consistent with earlier observations.^7,9,38,39^ BM analysis at 12 weeks post-BMT confirmed that DNMT3A loss of function promoted HSPC expansion. Indeed, CD45.2^+^ HSPCs in 10%*Dnmt3a*^R878H/+^BMT mice expanded significantly more than their counterparts in the control group, representing >50% of the Lin^−^Sca1^+^cKit^+^ (LSK) population (Figure S1C, left), whereas the absolute LSK cell numbers were comparable between the two groups (Figure S1C, right). Analysis of BM extracellular fluid for cytokines associated with expansion of CHIP-mutant clones^3,40^ revealed significantly increased tumor necrosis factor (TNF) and interferon (IFN)g concentrations in 10%*Dnmt3a*^R878H/+^BMT mice (Figure 1C top); the same cytokines, as well as interleukin (IL)-1b, were elevated in serum (Figure 1C bottom). In summary, competitive BMT with normal and mutant hematopoietic cells leads to increased systemic inflammation and selective clonal expansion of *Dnmt3a*^R878H/+^ HSPCs, which produce both myeloid and lymphoid cells, thus mimicking human *DNMT3A*-mediated CHIP.

Young adult mice are periodontally healthy; however, disruption of periodontal tissue homeostasis, e.g., due to immunoregulatory defects, leads to uncontrolled inflammation and bone loss (in both mice and humans).^41,42^ To determine whether the clonal expansion of *Dnmt3a*^R878H/+^ cells and the associated systemic inflammation (Figures 1B, 1C, S1A, and S1B) affect periodontal tissue homeostasis, we assessed the alveolar bone levels by measuring the distance between the cementoenamel junction (CEJ) and the alveolar bone crest (ABC). 10% *Dnmt3a*^R878H/+^BMT mice exhibited a significant increase over the mean CEJ-ABC distance of the control group (Figure 1D). Consistently, 10%*Dnmt3a*^R878H/+^BMT mice displayed elevated gingival mRNA expression of pro-inflammatory cytokines (e.g., IL-23 and IL-17) and pro-osteoclastogenic factors (e.g., Tnfsf11/RANKL) (Figure 1E). Measurement of bone mass of the femur and spine revealed no significant differences between 10%*Dnmt3a*^R878H/+^BMT and controls (Figures S1D–S1F), except for a modest decrease (8.9%) in trabecular bone volume to total bone volume fraction (BV/TV) in 10% *Dnmt3a*^R878H/+^BMT mice (Figure S1D, left bar graph). Therefore, naturally occurring periodontal bone loss in 10%*Dnmt3a*^R878H/+^ BMT mice could not be attributed to general bone defects.

We thus next investigated potential local mechanisms in the gingival tissue. We first performed flow cytometry to assess the frequency (% in total CD45^+^ cells) and absolute numbers of CD45.2^+^ *Dnmt3a*^R878H/+^ myeloid and T cells (key cells involved in periodontitis^43,44^) relative to CD45.2^+^ *Dnmt3a*^*+/+*^ cells. We found significantly increased abundance (in terms of both frequency and numbers) of CD45.2^+^ *Dnmt3a*^R878H/+^ neutrophils (Figure 1F), monocytes (Figure 1G), and T cells (Figure 1H), compared with the abundance of their respective CD45.2^+^ *Dnmt3a*^*+/+*^ counterparts. Fluorescence-activated cell sorting (FACS) analysis of the gingival tissue also revealed the presence of osteoclastogenic macrophages (OCMs; Ly6C^int^ I-A^+^/I-E^+^CX3CR1^hi^F4/80^+^) (Figure 1I), a macrophage subset that differentiates into osteoclasts in inflamed tissue.^45^ The numbers and frequency of CD45.2^+^ *Dnmt3a*^R878H/+^ OCM among the total OCM population were significantly increased in 10%*Dnmt3a*^R878H/+^BMT mice relative to their CD45.2^+^ *Dnmt3a*^*+/+*^ counterparts in 10%*Dnmt3a*^+/+^BMT mice (Figure 1I). In *ex vivo* experiments, lipopolysaccharide (LPS)-stimulated *Dnmt3a*^R878H/+^ splenic neutrophils and monocytes released higher amounts of IL-1β, IL-6, and TNF than their respective *Dnmt3a*^*+/+*^ counterparts (Figure S1G). Moreover, phorbol myristate acetate (PMA)-stimulated *Dnmt3a*^R878H/+^ peripheral blood neutrophils displayed increased production of intracellular reactive oxygen species (ROS) relative to *Dnmt3a*^*+/+*^ controls (Figure S1H). However, *Dnmt3a*^R878H/+^ and *Dnmt3a*^*+/+*^ blood neutrophils or splenic monocytes exhibited comparable *ex vivo* chemotactic capacity in response to chemokine (C-X-C motif) ligand (CXCL)1 or CXCL2 (Figure S1I left; neutrophils) or to monocyte chemoattractant protein (MCP)-1 (Figure S1I right; monocytes). Therefore, the increased periodontal inflammation and bone loss in 10% *Dnmt3a*^R878H/+^BMT mice could be attributed, at least in part, to the increased abundance of hyper-inflammatory *Dnmt3a*^R878H/+^ myeloid cells, including OCMs. Moreover, in the mutant setting (10%*Dnmt3a*^R878H/+^BMT mice), even WT cells could exhibit altered inflammatory activity, presumably via paracrine stimulation from neighboring *Dnmt3a*^R878H/+^ cells. In support, gingival WT myeloid (CD45.1^+^CD11b^+^) cells isolated from 10%*Dnmt3a*^R878H/+^BMT mice exhibited higher mRNA expression levels of key inflammatory cytokines than their counterparts from the WT setting (10%*Dnmt3a*^+/+^BMT mice) (Figure S1J).

### Clonal expansion of *Dnmt3a*^R878H/+^ cells exacerbates experimental periodontitis and arthritis

**Figure 2.**
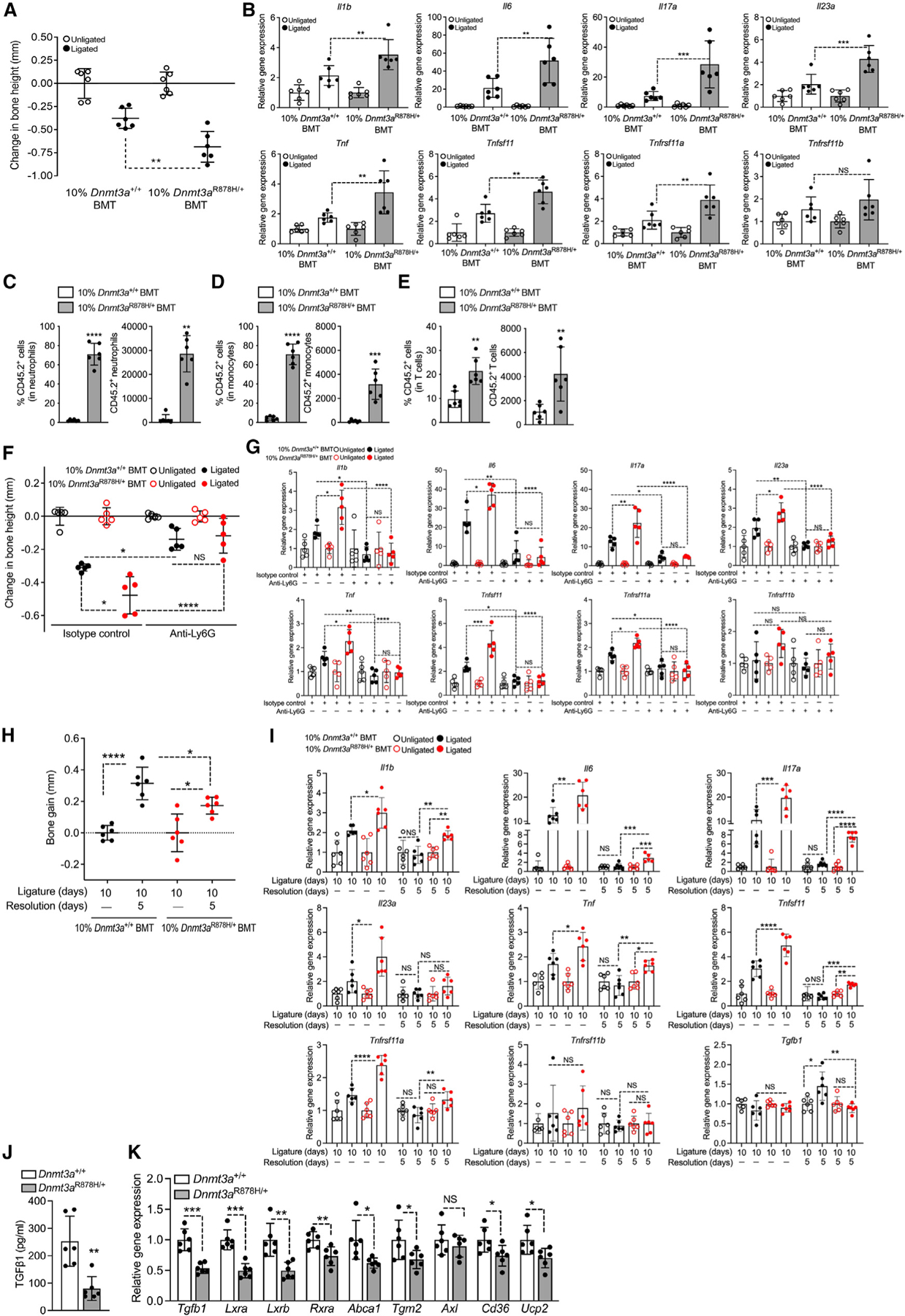
Clonal expansion of *Dnmt3a*^R878H/+^ cells increases the severity of experimental periodontitis and impairs inflammation resolution (A–E) BMT was performed as in Figure 1A, and after 12 weeks, recipient mice were subjected to ligature-induced periodontitis (LIP). (A) Bone loss in LIP-subjected mice. (B) Relative gingival mRNA expression of indicated molecules. (C–E) Percentage (right) and absolute numbers (left) of CD45.2^+^ neutrophils (CD11b^+^Ly6G^+^) (C), CD45.2^+^ monocytes (CD11b^+^Ly6C^+^Ly6G^−^) (D), and CD45.2^+^ T cells (CD3^+^) (E) in the gingival tissue. (F and G) Similar experiment as in (A) and (B) except that, at 12 weeks post-BMT, recipient mice were i.p. administered anti-Ly6G (or isotype control). (F) Bone loss in LIP-subjected mice. (G) Relative gingival mRNA expression of indicated molecules. (H and I) 12 weeks post-BMT 10%*Dnmt3a*^+/+^BMT and 10%*Dnmt3a*^R878H/+^BMT mice were subjected to LIP for 10 days followed, or not, by 5 days without ligatures to enable resolution. (H) Bone gain relative to the bone heights at day 10. (I) Relative gingival mRNA expression of indicated molecules. (J and K) BMDM from *Dnmt3a*^*+/+*^ or *Dnmt3a*^*R878H/+*^ mice were allowed to efferocytose apoptotic neutrophils for 3 h. TGF-β1 protein levels in the supernatants (J) and relative mRNA expression of indicated molecules (K). Data are means ± SD (*n* = 5–6 mice/group). *p < 0.05, **p < 0.01, ***p < 0.001, ****p < 0.0001; NS, not significant. Student’s unpaired t test (A, C–E, J, and K) except for (C right) (Mann-Whitney U test); one-way ANOVA with Tukey’s multiple comparison test (B and F–I).

**Figure 3.**
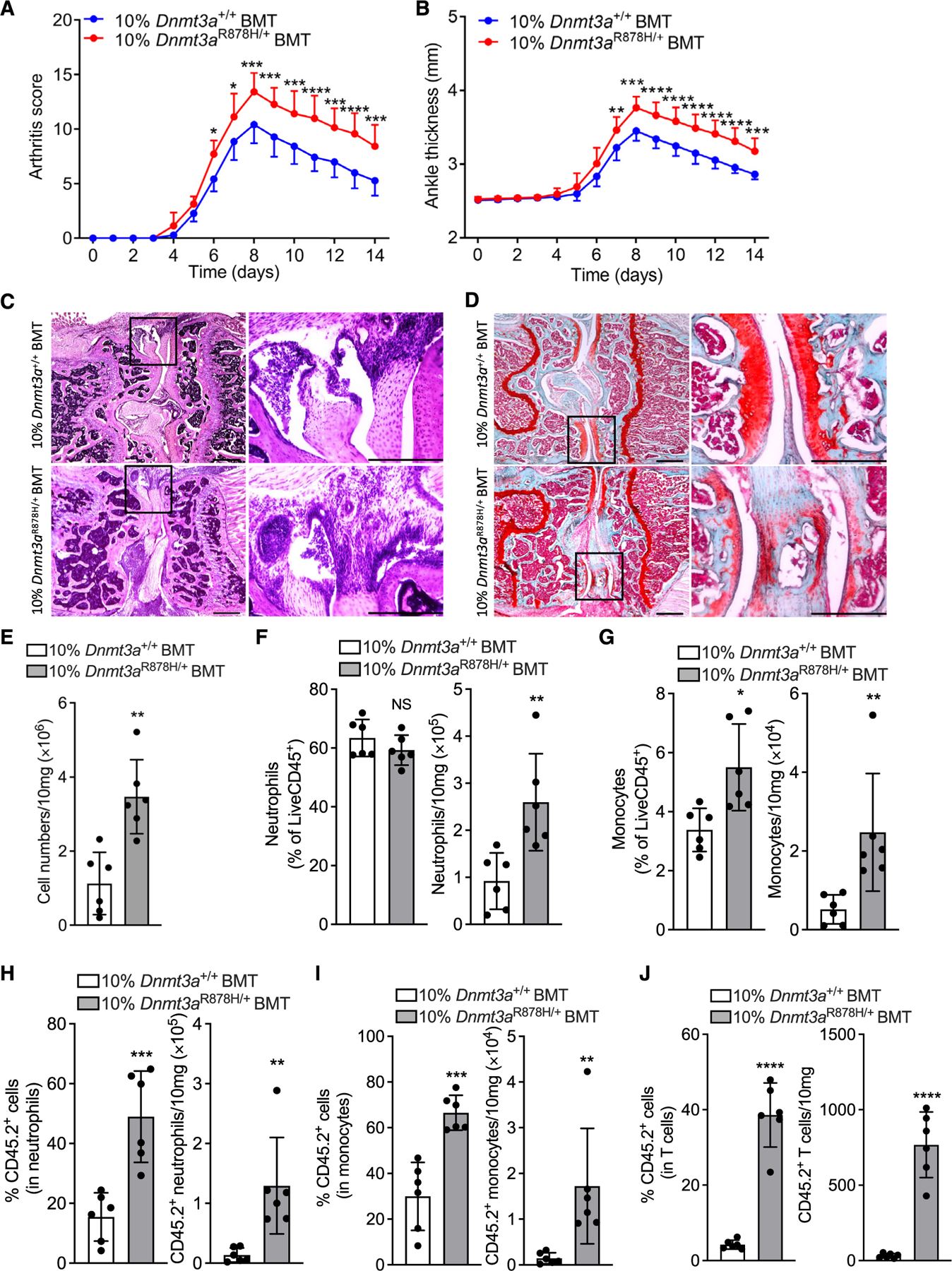
Clonal expansion of *Dnmt3a*^R878H/+^ cells increases the severity of inflammatory arthritis BMT was performed as in Figure 1A, and after 12 weeks, recipient mice were subjected to CAIA. (A and B) (A) Clinical arthritis scores and (B) joint thickness at indicated time points. (C and D) (C) Representative images of H&E and (D) of safranin-O staining of tissue sections from knee joints harvested on day 7. Scale bars, 500 μm. (E–G) (E) Total cell numbers and (F) percentage (left) and numbers (right) of neutrophils (CD45^+^CD11b^+^Ly6G^+^) and (G) of monocytes (CD45^+^CD11b^+^Ly6C^+^Ly6G^−^) in the synovium of knee joints on day 7. (H–J) (H) Percentage (left) and total counts (right) of CD45.2^+^ neutrophils (CD11b^+^Ly6G^+^), (I) CD45.2^+^ monocytes (CD11b^+^Ly6C^+^Ly6G^−^), and (J) CD45.2^+^ T cells (CD3^+^) in the synovium. Data are means ± SD (*n* = 6–7 mice/group). *p < 0.05; **p < 0.01; ***p < 0.001; ****p < 0.0001; NS, not significant. Two-way ANOVA with repeated measures and Sidak’s post-test for comparison with 10%*Dnmt3a*^*+/+*^ BMT mice (A and B); Student’s unpaired t test (E, F, J, and G–I left); Mann-Whitney U test (G–I right).

We next determined whether clonal expansion of *Dnmt3a*^R878H/+^ hematopoietic cells exacerbates pathology in induced models of disease. Specifically, 12 weeks post-BMT, 10%*Dnmt3a*^R878H/+^ BMT mice and controls were subjected to ligature-induced periodontitis (LIP) (Figure 2) or collagen antibody-induced arthritis (CAIA) (Figure 3). Compared with controls, 10% *Dnmt3a*^R878H/+^BMT mice exhibited significantly increased ligature-induced bone loss (Figure 2A) associated with elevated gingival mRNA expression of pro-inflammatory cytokines (e.g., IL-6, IL-17, and IL-23) and pro-osteoclastogenic molecules (Tnfsf11/RANKL and Tnfrsf11a/RANK) (Figure 2B). Moreover, 10%*Dnmt3a*^R878H/+^BMT mice displayed significantly increased frequency and total counts of CD45.2^+^ *Dnmt3a*^R878H/+^ neutrophils (Figure 2C), monocytes (Figure 2D), and T cells (Figure 2E), compared with the frequency and counts of their corresponding counterparts in 10%*Dnmt3a*^+/+^BMT controls. Neutrophils constituted the majority of gingival myeloid cells (Figures 2C and 2D) and can contribute to inflammatory bone loss by releasing pro-inflammatory cytokines and ROS.^46^ After antibody-mediated neutrophil depletion, bone loss (Figure 2F) and periodontal inflammation (Figure 2G) were significantly diminished in LIP-subjected 10%*Dnmt3a*^R878H/+^BMT mice and were indistinguishable from bone loss and periodontal inflammation in similarly treated LIP-subjected 10%*Dnmt3a*^+/+^BMT mice. The finding that neutrophil depletion has a greater impact in mice that received mutant clones substantiates the disease-provoking effect of the R878H mutation in neutrophils.

Ligature removal for 5 days in WT mice previously subjected to 10-day LIP leads to inflammation resolution and bone regeneration.^47,48^ Accordingly, 10%*Dnmt3a*^+/+^BMT mice subjected to the same LIP/resolution model showed ample bone regeneration (Figure 2H). However, by comparison, 10%*Dnmt3a*^R878H/+^BMT mice exhibited significantly diminished bone regeneration (Figure 2H). Consistently, upon resolution, the expression of inflammatory/osteoclastogenic genes (*Il1b*, *Il6*, *Il17a*, *Il23a*, *Tnf*, *Tnfsf11*, and *Tnfrsf11a*) returned to background levels (no significant difference vs. unligated internal control) in the WT setting, whereas almost all of these genes were still expressed at significantly higher levels (vs. unligated internal control) in the mutant setting (Figure 2I). Moreover, the expression of transforming growth factor b1 (*Tgfb1*), which plays a major role in inflammation resolution and tissue repair,^49,50^ was significantly lower during the resolution phase in 10%*Dnmt3a*^R878H/+^BMT mice compared with controls (Figure 2I, bottom right). Macrophages mediate inflammation resolution through phagocytosis of apoptotic neutrophils (efferocytosis).^49^ *Dnmt3a*^*R878H/+*^ BM-derived macrophages (BMDMs) co-cultured with apoptotic neutrophils (and the opsonin DEL-1-Fc that enhances efferocytosis^49^) released significantly reduced TGF-β1 protein levels relative to *Dnmt3a*^*+/+*^ BMDM (Figure 2J). In the same efferocytosis system, *Dnmt3a*^*R878H/+*^ BMDM displayed significantly reduced mRNA expression of *Tgfb1* and additional markers of inflammation resolution (*Lxra, Lxrb, Rxra, Abca1, Tgm2, Axl, Cd36*, and *Ucp2*)^49^ relative to *Dnmt3a*^*+/+*^ BMDM (Figure 2K). Therefore, the defective periodontal inflammation resolution and bone regeneration in the mutant setting (Figures 2H and 2I) could, at least in part, be attributed to the reduced ability of *Dnmt3a*-mutant macrophages to generate pro-resolving/proregenerative molecules upon efferocytosis.

Upon induction of CAIA in 10%*Dnmt3a*^R878H/+^BMT mice and controls, the former exhibited significantly increased clinical arthritis score (Figure 3A) and ankle joint thickness (Figure 3B), as well as aggravated histopathology in knee joints, as revealed by hematoxylin and eosin (H&E) assessment of loss of joint architecture, inflammatory cell infiltrate, and synovial hyperplasia (Figure 3C). Moreover, safranin-O staining of knee joint tissue sections displayed increased cartilage loss and roughening of the articular surface in the mutant setting (Figure 3D). 10% *Dnmt3a*^R878H/+^BMT mice also showed elevated cellularity in the synovium (Figure 3E) with increased abundance of neutrophils (Figure 3F) and monocytes (Figure 3G). Moreover, 10% *Dnmt3a*^R878H/+^BMT mice showed significantly increased frequency and total counts of CD45.2^+^ *Dnmt3a*^R878H/+^ neutrophils (Figure 3H), monocytes (Figure 3I), and T cells (Figure 3J). Overall, expansion of *Dnmt3a*-CHIP clones and infiltration of peripheral tissues with mutant leukocytes aggravates experimentally induced periodontitis and arthritis.

### scRNA-seq reveals insights into inflammatory pathology in DNMT3A-driven CHIP

**Figure 4.**
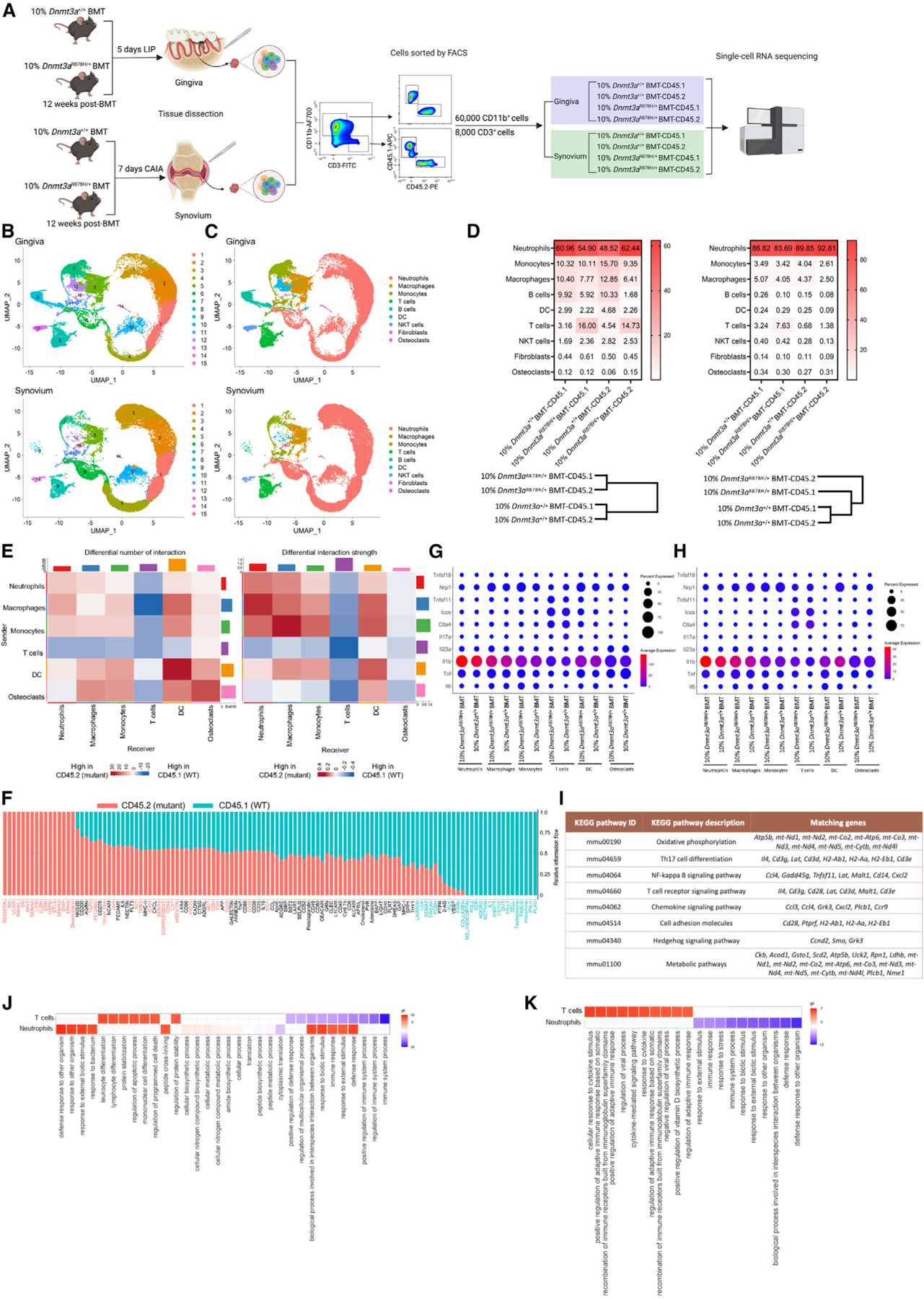
Single-cell RNA sequencing analysis of gingival and synovial cells (A) Experimental design. (B and C) Two-dimensional UMAP representation of 69,509 cells, according to (B) sample origin and results of clustering and (C) results of annotation. (D) Heatmap visualization of the distribution of sequenced cells in gingiva (top left) and synovium (top right) and cluster analysis of cell types in gingiva (bottom left) and synovium (bottom right), normalized for the number of cells per sample in the dataset. (E and F) CellChat analysis of intercellular communication networks in CD45.2^+^ (mutant) and CD45.1^+^ (WT) cells from 10%*Dnmt3a*^R878H/+^BMT mice. (E) Heatmap of differential number and strength of possible interactions between any two of the indicated analyzed cell populations in gingiva (red, increased interaction in mutant cells; blue, increased interaction in WT cells). (F) Visualization of the overall information flow of each indicated signaling pathway by calculating the sum of communication probability among all pairs of synovial cell groups in the inferred network. The red and green colors indicate increased enrichment in mutant or WT cells, respectively. (G and H) Gene expression levels in distinctly defined CD45.2^+^ cell types from gingiva (G) or synovium (H). (I) List of gingival upregulated enriched genes in CD45.2^+^ (*Dnmt3a*^R878H/+^) cells in the indicated KEGG pathway terms analyzed by STRING. (J and K) Top 10 significantly enriched GO terms sorted by PANTHER based on significantly upregulated or downregulated genes (in 10%*Dnmt3a*^R878H/+^BMT-CD45.2 vs. 10%*Dnmt3a*^+/+^BMT-CD45.2) in neutrophils and T cells from gingiva (J) and synovium (K) (Fisher test with FDR-correction, *p* < 0.05). See also Figures S2 and S3.

To better understand how *Dnmt3a*^R878H/+^ leukocytes exacerbate experimental periodontitis and arthritis, we performed single-cell RNA sequencing (scRNA-seq) in sorted CD45.2^+^CD11b^+^, CD45.1^+^CD11b^+^, CD45.2^+^CD3^+^, and CD45.1^+^CD3^+^ cells from 10%*Dnmt3a*^R878H/+^BMT and control mice, which were subjected to 5-day LIP or 7-day CAIA 12 weeks post-BMT. 60,000 CD11b^+^ (CD45.2^+^ or CD45.1^+^) cells and 8,000 CD3^+^ (CD45.2^+^ or CD45.1^+^) cells from each tissue—gingiva or synovium—were mixed, respectively (separately for each tissue), followed by scRNA-seq (Figure 4A). Two-dimensional uniform manifold approximation and projection (UMAP) of 34,335 gingival cells and 35,174 synovial cells and clustering partitioned cells into 15 clusters (C1–C15) (Figures 4B and S2A). Annotation by typical marker genes revealed 9 cell types in gingiva (G) and synovium (S), including neutrophils, monocytes, macrophages, B cells, T cells, dendritic cells, osteoclasts, natural killer T (NKT) cells, and fibroblasts (likely contaminants) (Figures 4C and 4D). C1–C4: C6 and C10 comprised neutrophils; C5: macrophages; C8: T cells; C7 and C11: monocytes; C9: B cells; C12: dendritic cells (DC); C13: NKT cells; C14: fibroblasts; and C15: osteoclasts (Figures 4B and 4C). The annotation was validated by the demonstration of expression of cell type-specific markers (Figures S2B and S2C). Cluster analysis of cell types exhibited two distinct clusters in gingiva: the 10%*Dnmt3a*^R878H/+^BMT-CD45.1 group (WT cells in the mutant setting) was more similar to the 10% *Dnmt3a*^R878H/+^BMT-CD45.2 group (mutant cells in the mutant setting) than it was to the other 2 groups (10%*Dnmt3a*^+/+^BMT-CD45.2 and 10%*Dnmt3a*^+/+^BMT-CD45.1, i.e., WT cells (CD45.2^+^ or CD45.1^+^) in the WT setting) (Figure 4D, bottom left). In the synovium, the 10%*Dnmt3a*^R878H/+^BMT-CD45.1 group did not cluster with the CD45.2 (mutant) group and was more similar to, although still different from WT cells in the WT setting (Figure 4D, bottom right). The increased inflammatory environment in the mutant setting might thus affect WT cell populations in a manner that distinguishes them from WT cell populations in the WT setting.

To further investigate this notion, we explored intercellular communication networks (interaction numbers and strength) in CD45.2^+^ (mutant) and CD45.1^+^ (WT) cells from 10% *Dnmt3a*^R878H/+^BMT mice by performing comparative CellChat analysis.^51^ Differential interactions in cell-cell communication networks between CD45.2^+^ and CD45.1^+^ cells were determined and visualized, with red and blue color indicating, respectively, increased and decreased signaling in CD45.2^+^ mutant cells compared with CD45.1^+^ WT cells. The number and strength of putative signaling interactions within and between myeloid cell types (neutrophils, monocytes/macrophages, DC) generally increased in gingival mutant cells (Figure 4E), whereas only the strength of the signaling increased in synovial mutant cells (Figure S3D). By contrast, the number and strength of putative signaling interactions within T cells and between T cells and myeloid cells decreased in both gingival and synovial mutant cells (Figures 4E and S3D, respectively). There was a slight increase in putative signaling between myeloid cells (macrophages and DC) and osteoclasts in both gingiva and synovium (mutant setting) (Figures 4E and S3D). We moreover compared the information flow (overall communication probability) across CD45.2^+^ (mutant) and CD45.1^+^ (WT) cells for specific signaling pathways. Remarkably increased information flow in gingival mutant cells included resistin, midkine (MK), vascular cell adhesion protein (VCAM), receptor activator of nuclear factor-kB ligand (RANKL), and sialic acid-binding Ig-like lectin 1 (SN) signaling (Figure 4F). Among them, resistin is a potential biomarker in periodontitis,^52,53^ MK and VCAM are putative risk factors for periodontitis,^54,55^ and RANKL drives inflammatory bone loss.^56^ Remarkably increased information flow in synovial mutant cells included angiopoietin-like proteins (ANGPTL), lymphotoxin-a (LT), adiponectin, collagen, and IL-6 (Figure S3E). Of these, LT activates nuclear factor kB (NF-kB) signaling in chondrocytes and contributes to rheumatoid arthritis^57^; adiponectin is increased in the synovial fluid and promotes arthritis^58,59^; IL-6 is a therapeutic target in rheumatoid arthritis.^60^ Together, there appears to be increased autocrine and paracrine signaling probabilities between different cell types during experimental periodontitis and arthritis, and these inferences are consistent with a recent CellChat analysis of DNMT3A-mutant cells from the peripheral blood of heart failure patients.^61^ Mutant cells might thus promote inflammation not only in a cell-intrinsic manner but also via paracrine effects by activating neighboring WT cells, consistent with our finding that WT myeloid cells from the *Dnmt3a*-mutant setting expressed significantly higher levels of *Il1b*, *Il6*, *Il23a*, and *Tnf* than WT myeloid cells from the WT setting (Figure S1J).

Further analysis of the scRNA-seq data (with Seurat’s DotPlot function) showed that—in the gingival mutant setting—CD45.2^+^ (mutant) neutrophils and macrophages expressed more *Il1b*, *Tnf*, and *Il23a*; monocytes expressed more *Il6*, *Tnf*, *Il23a*, and *Tnfsf11* (RANKL); DC expressed more *Tnf*, *Il23a*, and *Tnfsf11*; and T cells expressed more *Tnfsf11* and less *Ctla4*, as compared with their CD45.2^+^ (WT) counterparts in the WT setting (Figure 4G). Similarly, in the synovial mutant setting, CD45.2^+^ (mutant) cells in general expressed inflammatory/osteoclastogenic cytokines at higher levels than their CD45.2^+^ (WT) counterparts in the WT setting (Figure 4H). Moreover, in the gingival mutant setting, CD45.1^+^ (WT) macrophages expressed more *Tnf*; monocytes expressed more *Il6* and *Tnfsf11*; and DC expressed more *Tnfsf11*, compared with their CD45.1^+^ (WT) counterparts in the WT setting (Figure S3A). In the synovial mutant setting, CD45.1^+^ (WT) DC expressed more *Il23a* relative to their CD45.1^+^ (WT) counterparts in the WT setting (Figure S3B). These observations are consistent with our above-described findings that the R878H mutation renders the affected leukocytes more pro-inflammatory than their WT counterparts and that WT cells in the mutant setting are more pro-inflammatory than WT cells in the WT setting. According to Search Tool for the Retrieval of Interacting Genes/Proteins (STRING) analysis, the enriched upregulated genes in CD45.2^+^ (*Dnmt3a*^R878H/+^) cells in the mutant setting indicated enhanced activation of several Kyoto encyclopedia of genes and genomes (KEGG) inflammatory pathways (e.g., “Th17 cell differentiation” and “NF-kB signaling pathway”) relative to CD45.2^+^ (*Dnmt3a*^*+/+*^) cells in the WT setting (Figures 4I and S3C, for gingiva and synovium, respectively).

For Gene Ontology (GO) analysis, we focused on cell types with critical roles in bone loss disorders, namely, neutrophils, T cells, and osteoclasts.^43,62–65^ GO enrichment analysis in gingival neutrophils showed that “immune response,” “defense response,” and related terms were enriched in the significantly upregulated genes in the 10%*Dnmt3a*^R878H/+^BMT-CD45.2 group (vs. 10%*Dnmt3a*^+/+^BMT-CD45.2 group) (Figure 4J). GO enrichment analysis in synovial neutrophils did not reveal any significantly enriched GO terms in the upregulated genes, whereas the “defense response to other organism” and related terms were among the top 10 enriched GO terms in the significantly downregulated genes (Figure 4K). GO enrichment analysis of gingival T cells showed that the GO terms “lymphocyte differentiation” and “regulation of apoptotic process” were among the enriched terms in the significantly upregulated genes in the 10%*Dnmt3a*^R878H/+^BMT-CD45.2 group, whereas “immune system process” and “defense response” were among the significantly enriched terms in the downregulated genes (Figure 4J). GO enrichment analysis of synovial T cells showed that “cellular response to cytokine stimulus,” and “positive regulation of adaptive immune response” were among the top 10 enriched GO terms in the significantly upregulated genes, whereas there were no enriched GO terms in the significantly downregulated genes (Figure 4K).

Analysis in all 15 clusters of the expression of *Acp5* and *Ctsk*, which are major markers of osteoclasts,^66,67^ revealed that these markers were most highly expressed in C15 (Figures S2B and S2C), hence identified as osteoclasts. It should be noted that gingival tissues dissected from LIP-subjected mice do contain tartrate-resistant acid phosphatase (TRAP)-stained osteoclasts in areas adjacent to the underlying bone (Figure S2D); similarly, osteoclasts are found within the synovium at sites proximal to the bone.^68^ The frequency of osteoclasts (C15) in the 10% *Dnmt3a*^R878H/+^BMT-CD45.2 group from gingiva and synovium was increased relative to the 10%*Dnmt3a*^+/+^BMT-CD45.2 control, especially in the gingiva (2.5-fold; Figure 4D, left). Because the influence of CHIP mutations on osteoclasts and T cells is less well understood than that on myeloid cells, we next investigated osteoclastogenesis and T cell function in the context of DNMT3A/CHIP-driven inflammatory disease.

### The R878H mutation is associated with enhanced osteoclastogenesis and impaired Treg cell immunosuppressive activity

**Figure 5.**
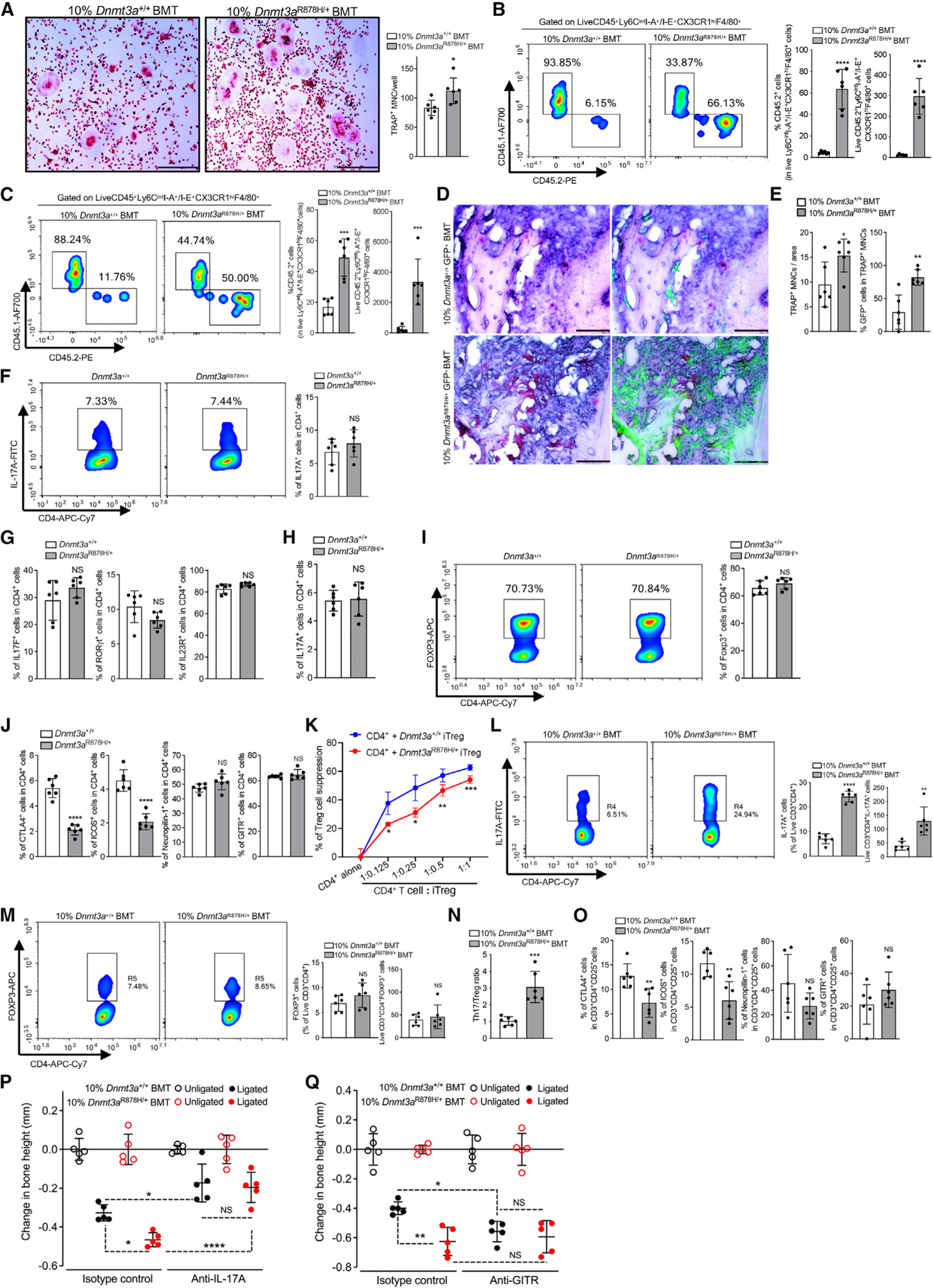
The R878H mutation is associated with enhanced osteoclastogenesis and impaired Treg immunosuppressive activity (A) RANKL-induced osteoclastogenesis using BM-derived OCP (CD11b^−/lo^Ly6C^hi^ cells) from 10%*Dnmt3a*^R878H/+^BMT and 10%*Dnmt3a*^+/+^BMT mice. Representative TRAP-stained images (left) and numbers of TRAP-positive multinucleated cells (MNCs) (right). Scale bars, 500 μm. (B and C) BMT was performed as in Figure 1A, and after 12 weeks post-BMT, recipient mice were subjected to LIP (B) or CAIA (C). Representative FACS plots (left), percentage (middle), and numbers (right) of CD45.2^+^ Ly6C^int^I-A^+^/I-E^+^CX3CR1^hi^F4/80^+^ osteoclastogenic macrophages in gingival tissue (B) or synovium (C). (D and E) Similar BMT as above using GFP-labeled donor cells. 10%*Dnmt3a*^R878H/+^GFP^+^BMT and 10%*Dnmt3a*^*+*/+^GFP^+^BMT mice were subjected to LIP 12 weeks post-BMT. (D) Representative TRAP-stained images without (left) and with GFP signal (right). (E) Numbers of TRAP^+^ MNCs per area (left) and percentages of GFP^+^ cells in TRAP^+^ MNCs (right). Scale bars, 100 μm. (F–H) Naive splenic CD4^+^ T cells from *Dnmt3a*^*+*/+^ or *Dnmt3a*^R878H/+^ mice were subjected to Th17-differentiation assay for 3 days under pathogenic (F and G) or non-pathogenic (H) conditions^70^ (see STAR Methods). (F) Representative FACS plots (left) and percentage (right) of CD4^+^IL-17A^+^ (Th17) cells. (G) Percentages of cells expressing the indicated Th17-associated markers. (H) Percentage of CD4^+^IL-17A^+^ (Th17) cells (non-pathogenic conditions). (I and J) Naive splenic CD4^+^ T cells from *Dnmt3a*^*+*/+^ or *Dnmt3a*^R878H/+^ mice were subjected to Treg-differentiation assay for 3 days. (I) Representative FACS plots (left) and percentage (right) of induced CD4^+^FOXP3^+^ (iTreg) cells. (J) Percentages of cells expressing indicated markers associated with Treg cell function. (K) Suppression of carboxyfluorescein succinimidyl ester (CFSE)-labeled CD4^+^CD25^−^ T cell division by *Dnmt3a*^*+/+*^ iTregs or *Dnmt3a*^R878H/+^ iTregs. (L–O) BMT was performed as in Figure 1A, and after 12 weeks, gingival tissue was processed for FACS. Representative FACS plots (left), percentage (middle), and absolute numbers (right) of Th17 (live CD3^+^CD4^+^IL-17A^+^) cells (L) and of Treg (live CD3^+^CD4^+^FOXP3^+^) cells (M). Corresponding Th17/Treg ratio in the gingival tissue (N). Percentage of CD3^+^CD4^+^CD25^+^ T cells expressing indicated Treg-associated functional markers (O). (P and Q) BMT was performed as in Figure 1A, and after 12 weeks, recipient mice were subjected to LIP after local treatment with anti-IL-17A (or isotype control) (P) or after systemic treatment with anti-GITR (or isotype control) to deplete Tregs (Q). Bone loss was determined 5 days post-LIP. Data are means ± SD (*n* = 5–6 replicates (mice or cultures)/group). *p < 0.05; **p < 0.01; ***p < 0.001; ****p < 0.0001; NS, not significant. Student’s unpaired t test (A–C, E–J, and L–O) except for (G; IL-23R) (Mann-Whitney U test); two-way ANOVA with repeated measures and Sidak’s multiple comparisons test (K); one-way ANOVA with Tukey’s multiple comparisons test (P and Q). See also Figure S4.

Earlier work has identified a CD11b^−/lo^Ly6C^hi^ monocytic population in the BM that is induced upon inflammation and is highly enriched in osteoclast precursors (OCPs).^69^ FACS analysis to identify this OCP-enriched population in the BM of LIP-subjected 10%*Dnmt3a*^R878H/+^BMT mice and controls revealed no significant differences between the two groups regarding the frequency—among BM cells—of CD11b^−/lo^Ly6C^hi^ cells or their total numbers in the BM (Figure S4A). However, the frequency and total numbers of CD45.2^+^ CD11b^−/lo^Ly6C^hi^ cells in the mutant setting were significantly higher than the frequency and total numbers of their CD45.2^+^ counterparts in the WT setting (Figure S4B). Thus, although the presence of the hematopoietic-specific R878H mutation in the BM is not associated with expansion of the CD11b^−/lo^Ly6C^hi^ population, within this population, the donor-derived (CD45.2^+^) mutant cells in 10% *Dnmt3a*^R878H/+^BMT mice greatly outnumber their CD45.2^+^ WT counterparts in the WT setting. To determine whether the R878H mutation in this OCP-enriched population is associated with enhanced osteoclastogenesis, we performed a RANKL-induced osteoclast differentiation assay. CD11b^−/lo^Ly6C^hi^ cells sorted from the BM of 10%*Dnmt3a*^R878H/+^BMT mice yielded more osteoclasts (TRAP^+^ multinucleated cells [MNCs]) than did CD11b^−/lo^Ly6C^hi^ cells sorted from the BM of 10% *Dnmt3a*^+/+^BMT controls (Figure 5A).

Using FACS, we analyzed the gingival tissue and synovium of LIP- or CAIA-subjected 10%*Dnmt3a*^+/+^BMT and 10% *Dnmt3a*^R878H/+^BMT mice for the presence of Ly6C^int^I-A^+^/I-E^+^CX3CR1^hi^F4/80^+^ cells, an OCP macrophage subset that originates from circulating BM-derived cells (OCMs).^45^ The numbers and frequency of CD45.2^+^ *Dnmt3a*^R878H/+^ OCM (in the mutant setting) were significantly increased relative to their *Dnmt3a*^*+/+*^ counterparts (in the WT setting) in gingiva and synovium (Figures 5B and 5C). We next interrogated whether this difference leads to increased osteoclastogenesis. To facilitate the identification of donor-derived (CD45.2^+^) osteoclasts, we generated *Dnmt3a*^R878H/+^GFP^+^ mice (and *Dnmt3a*^+/+^GFP^+^ controls) by crossing *Dnmt3a*^R878H/+^ (or *Dnmt3a*^*+/+*^) mice with ubiquitin C (UBC)-GFP transgenic mice.^71,72^ The generated mice were used as BMT donors according to the standard protocol (Figure 1A). We counted the numbers of GFP-labeled CD45.2^+^ *Dnmt3a*^R878H/+^ and CD45.2^+^ *Dnmt3a*^*+/+*^ osteoclasts in “10%*Dnmt3a*^R878H/+^GFP^+^BMT” and “10%*Dnmt3a*^*+*/+^ GFP^+^BMT” mice, respectively, which were subjected to LIP 12 weeks post-BMT. Compared with controls, “10% *Dnmt3a*^R878H/+^ GFP^+^BMT” mice displayed not only significantly higher numbers of total osteoclasts (TRAP^+^ MNCs) but also significantly increased frequency of donor-derived (CD45.2^+^ GFP^+^) osteoclasts (Figures 5D and 5E). *Dnmt3a*^R878H/+^ osteoclasts were thus present at a higher frequency than *Dnmt3a*^*+/+*^ osteoclasts (Figure 5E, right).

Given that IL-17-expressing CD4^+^ T helper 17 (Th17) cells and FOXP3^+^CD4^+^ regulatory T (Treg) cells play critical roles in the pathogenesis of both periodontitis and arthritis,^63,73^ we examined whether the R878H mutation affects the differentiation of naive T cells to Th17 or Tregs. In a standard naive CD4^+^ T cell differentiation assay based on polyclonal stimulation with anti-CD3/anti-CD28 and appropriate polarizing cytokines,^74^ *Dnmt3a*^*+/+*^ and *Dnmt3a*^R878H/+^ splenic naive CD4^+^ T cells exhibited comparable Th17 differentiation under the influence of TGF-β1, IL-6, IL-1β, and IL-23 (“pathogenic” conditions^70^), as evidenced by similar frequencies of generated IL-17A^+^CD4^+^ T cells (Figure 5F) and comparable expression of additional Th17-associated markers (Figure 5G). Comparable Th17 differentiation was also observed when “non-pathogenic” (TGF-β1 and IL-6) conditions^70^ were used (Figure 5H). Similarly, Treg-differentiation assays with *Dnmt3a*^*+/+*^ or *Dnmt3a*^R878H/+^ splenic naive CD4^+^ T cells (stimulated with anti-CD3/anti-CD28 and IL-2 plus TGF-β1^74^) revealed comparable frequencies of generated CD4^+^FOXP3^+^ (Treg) cells in the two groups (Figure 5I). Further analysis of markers associated with the Treg suppressive function showed that the frequencies of CTLA4- and inducible co-stimulator (ICOS)-expressing (but not of neuropilin-1- and Glucocorticoid-Induced TNFR-Related [GITR] protein-expressing) cells were markedly decreased in differentiation cultures of *Dnmt3a*^R878H/+^ CD4^+^ T cells compared with those of controls (Figure 5J). To determine if the function of *Dnmt3a*^R878H/+^ Tregs is affected, we sorted Tregs from the induction cultures of *Dnmt3a*^*+/+*^ or *Dnmt3a*^R878H/+^ CD4^+^ T cells (*Dnmt3a*^*+/+*^ iTregs and *Dnmt3a*^R878H/+^ iTregs) and compared their immunosuppressive activity. *Dnmt3a*^R878H/+^ iTregs exhibited significantly lower ability to inhibit the proliferation of CD4^+^ T cells after 72-h co-culture than *Dnmt3a*^*+/+*^ iTregs (Figure 5K).

Consistent with increased gingival mRNA expression of IL-17 and Th17-inducing cytokines (e.g., IL-6 and IL-23) (Figure 1E), 10%*Dnmt3a*^R878H/+^BMT mice exhibited significantly higher frequency and numbers of gingival CD4^+^IL-17^+^ (Th17) cells than controls (Figure 5L), although the frequency and numbers of gingival CD4^+^FOXP3^+^ (Treg) cells were comparable (Figure 5M). Consequently, 10%*Dnmt3a*^R878H/+^BMT mice exhibited a higher Th17/Treg ratio in the gingival tissue (Figure 5N). Moreover, the percentage of CTLA4- and ICOS-expressing (but not of neuropilin-1- and GITR-expressing) cells in CD4^+^CD25^+^ Treg cells was significantly decreased in the mutant setting (Figure 5O). These findings indicate that the R878H mutation does not affect the induction of Tregs but impairs their regulatory function, which in turn may contribute to unrestrained Th17 cell expansion and hence increased release of the pro-osteoclastogenic cytokine IL-17. Consistently, antibody-mediated neutralization of IL-17 diminished bone loss (Figure 5P) associated with decreased periodontal inflammation (Figure S4C) in LIP-subjected 10% *Dnmt3a*^R878H/+^BMT and 10%*Dnmt3a*^+/+^BMT mice, with the inhibitory effects being more pronounced in the mutant setting. Additionally, Treg depletion significantly enhanced LIP-induced bone loss in 10%*Dnmt3a*^+/+^BMT mice but not in 10% *Dnmt3a*^R878H/+^BMT mice (Figure 5Q), which contain functionally impaired *Dnmt3a*-mutant Tregs (Figure 5K). Consistently, Treg depletion enhanced periodontal inflammation selectively in 10%*Dnmt3a*^+/+^BMT mice (Figure S4D).

### Rapamycin inhibits clonal expansion of *Dnmt3a*^R878H/+^ cells and bone loss

**Figure 6.**
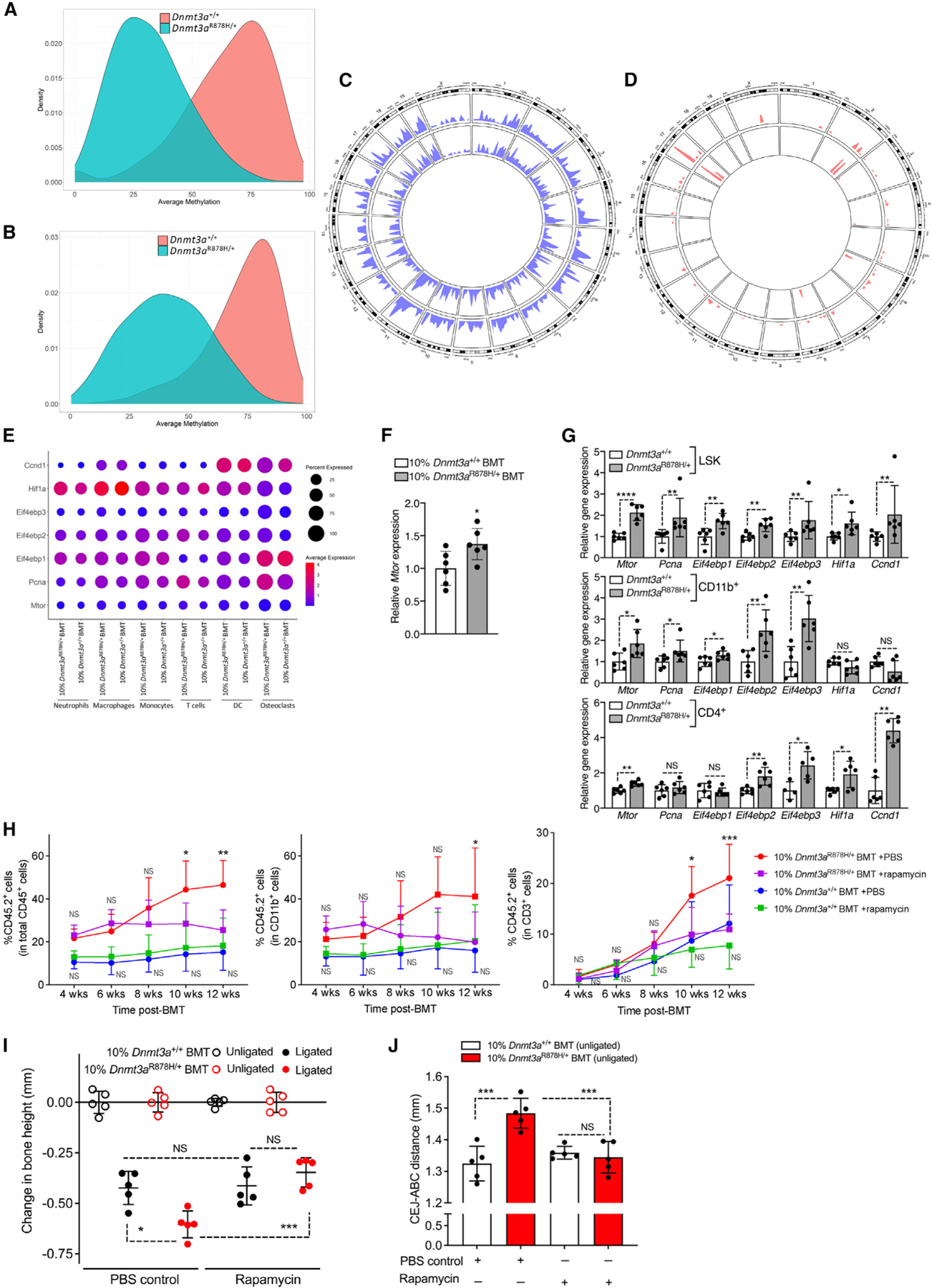
Rapamycin inhibits clonal expansion of *Dnmt3a*^R878H/+^ cells and inflammatory bone loss (A–D) Splenic CD11b^+^ and CD4^+^ cells from *Dnmt3a*^*+/+*^ and *Dnmt3a*^R878H/+^ mice (*n* = 4 mice/group) were processed for whole-genome bisulfite sequencing (WGBS). Density plot of average methylation values from WGBS of CD11b^+^ (A) and CD4^+^ (B) cells. Circos plot representation of genome-wide density of DNA hypomethylation (C) and hypermethylation (D) levels in CD11b^+^ and CD4^+^ cells. The inner track indicates the density of hypo/hyper-methylation in CD4^+^ cells, and the outer track indicates the density of hypo/hyper-methylation in CD11b^+^ cells. (E) Relative mRNA expression of *Mtor* and mTOR-regulated genes in the indicated CD45.2^+^ cell types from gingiva (data from scRNA-seq; see Figure 4). (F and G) BMT was performed as in Figure 1A, and cells were analyzed 12 weeks post-BMT. (F) Relative *Mtor* expression in BM cells and (G) relative mRNA expression of indicated molecules in BM LSK cells (Lin^−^cKit^+^Sca1^+^), splenic CD11b^+^ myeloid cells, and splenic CD4^+^ T cells from Dnmt3a^+/+^ or *Dnmt3a*^R878H/+^ mice. (H–J) BMT was performed as in Figure 1A, and recipient mice were treated with rapamycin or PBS control (see STAR Methods). 12 weeks post-BMT, all groups were subjected to LIP. (H) Percentage of CD45.2^+^ white blood cells within total CD45^+^ cells (left), CD11b^+^ myeloid cells (middle), and CD3^+^ T cells (right) in the peripheral blood. (I) Bone loss in LIP-subjected mice (relative to their unligated contralateral sites). (J) Measurement of CEJ-ABC distance in unligated sites of the same mice. Data are mean ± SD (F and G: *n* = 6 mice/group except *Eif4ebp3* in G bottom; *n* = 4–5 mice/group; H–J: *n* = 5 mice/group). *p < 0.05, **p < 0.01, ***p < 0.001, ****p < 0.0001. NS, not significant. Student’s unpaired t test (F and G) except in (G) for *Pcna* in LSK and CD11b^+^, *Eif4ebp1* in CD4^+^, *Eif4ebp3* in LSK and *Ccnd1* in LSK, CD11b^+^, and CD4^+^ (Mann-Whitney U test); two-way ANOVA with repeated measures and Tukey’s multiple comparisons test (H); one-way ANOVA with Tukey’s multiple comparison test (I and J). See also Figure S5.

To better understand the impact of the *Dnmt3a*-R878H mutation on the DNA methylation status of mature leukocytes, we conducted whole-genome bisulfite sequencing (WGBS) in splenic CD11b^+^ myeloid cells and CD4^+^ T cells from mutant (*Dnmt3a*^R878H/+^) mice and WT controls. Globally, the methylation levels across the genome in both CD11b^+^ and CD4^+^ cells from *Dnmt3a*^R878H/+^ mice were significantly decreased relative to their counterparts from *Dnmt3a*^*+/+*^ controls (Figures 6A and 6B). The average methylation values of CD11b^+^ and CD4^+^ cells from *Dnmt3a*^R878H/+^ mice were significantly lower than those from *Dnmt3a*^*+/+*^ mice at all regions of the genome examined (*p* ≤ 0.0001; Figures S5A and S5B). The genome-wide density of DNA hypomethylation in CD11b^+^ and CD4^+^ cells from *Dnmt3a*^R878H/+^ mice was similar (Figure 6C), although DNA hypermethylation levels in CD11b^+^ and CD4^+^ cells were different (Figure 6D). We used Bedtools to associate differentially methylated regions (DMRs) with genes and performed GO function analysis (top 15). The most hypomethylated DMRs in the CD11b^+^ and CD4^+^ cells from *Dnmt3a*^R878H/+^ mice were enriched in similar GO terms (Figure S5C). Hypermethylated DMRs in CD11b^+^ cells from *Dnmt3a*^R878H/+^ mice were only enriched in RNA metabolic process, whereas there were no enriched GO terms in CD4^+^ cells (Figure S5C). Significantly enriched KEGG pathway terms of hypomethylated genes (*Dnmt3a*^R878H/+^ vs. *Dnmt3a*^*+/+*^) in CD11b^+^ (Figure S5D) and CD4^+^ cells (Figure S5E) were similar and collectively indicated enhanced activation of inflammatory pathways, osteoclastogenesis, and mechanistic target of rapamycin (mTOR) signaling in *Dnmt3a*^R878H/+^ cells. Overall, WGBS analysis revealed common hypomethylation phenotypes in CD11b^+^ and CD4^+^ cells from *Dnmt3a*^R878H/+^ mice.

The results from our WGBS analysis revealing activation of mTOR signaling are consistent with a previous report that DNA hypomethylation associated with the R878H mutation in LSK cells leads to upregulation of mTOR, which in turn promotes overexpression of the cell-cycle regulator cyclin-dependent kinase-1.^75^ Consistently, scRNA-seq analysis of gingival cells (from LIP-subjected mice; Figure 4A) revealed elevated expression of *Mtor* and several mTOR-regulated genes in different cell types (especially osteoclasts) in the mutant setting, compared with the corresponding cell types in the WT setting (Figure 6E). Furthermore, BM cells from 10%*Dnmt3a*^R878H/+^BMT mice at steady state have significantly increased expression of *Mtor* compared with 10%*Dnmt3a*^+/+^BMT controls (Figure 6F). Also in line were our observations for upregulated mTOR expression in FACS-sorted *Dnmt3a*^R878H/+^ cells—namely, BM LSK cells, splenic CD11b^+^ myeloid cells, and splenic CD4^+^ T cells—as compared with their *Dnmt3a*^*+/+*^ counterparts (Figure 6G). The *Dnmt3a*^R878H/+^ cells, especially LSK cells, also exhibited increased expression of mTOR-regulated genes (*Pcna*, *Eif4ebp1*, *Eif4ebp2*, *Eif4ebp3*, H*if1a*, and *Ccnd1*^75–78^) (Figure 6G).

Based on the premise that elevated mTOR activation may drive aberrant expansion of *Dnmt3a*^R878H/+^ clones in our model, we hypothesized that the mTOR inhibitor rapamycin could reverse the increased bone loss associated with DNMT3A-driven CHIP. Starting from 2 weeks post-BMT, we treated 10% *Dnmt3a*^+/+^BMT and 10%*Dnmt3a*^R878H/+^BMT mice with PBS control or rapamycin twice per week for 4 weeks, followed by treatments once a week until sacrifice. CD45.2^+^ (*Dnmt3a*^R878H/+^) hematopoietic cells in PBS-treated 10%*Dnmt3a*^R878H/+^BMT mice expanded over time, whereas CD45.2^+^ hematopoietic cells in PBS-treated 10%*Dnmt3a*^+/+^BMT mice did not (Figure 6H left). Importantly, the expansion of CD45.2^+^ blood cells was suppressed by rapamycin in 10%*Dnmt3a*^R878H/+^BMT (vs. corresponding PBS-treated control) with differences reaching statistical significance at 10 and 12 weeks post-BMT (Figure 6H left). By contrast, rapamycin did not affect the frequency of CD45.2^+^ (*Dnmt3a*^*+*/+^) cells, within the total CD45^+^ leukocytes, in the peripheral blood of 10%*Dnmt3a*^+/+^BMT mice (Figure 6H left). The frequencies of CD45.2^+^ cells within the myeloid (CD11b^+^) and T cell (CD3^+^) lineages in 10%*Dnmt3a*^R878H/+^BMT mice were also decreased by rapamycin (Figure 6H, middle and right). All recipient groups were subjected to LIP at 12 weeks BMT. We observed significantly decreased bone loss in rapamycin-treated relative to PBS-treated 10%*Dnmt3a*^R878H/+^BMT mice (Figure 6I). By contrast, rapamycin had no effect on the bone loss developed by 10%*Dnmt3a*^+/+^BMT mice relative to their PBS-treated controls (Figure 6I). Consistently, rapamycin-treated 10%*Dnmt3a*^R878H/+^BMT mice also displayed significantly decreased gingival mRNA expression of several periodontitis-associated pro-inflammatory cytokines compared with PBS-treated 10%*Dnmt3a*^R878H/+^BMT controls (Figure S5F). To detect potential effects of rapamycin on naturally occurring periodontal bone loss, we also measured the bone heights (CEJ-ABC distance) in the unligated sites of PBS- or rapamycin-treated 10%*Dnmt3a*^+/+^BMT and 10%*Dnmt3a*^R878H/+^BMT mice. Consistent with the data in Figure 1D, PBS-treated 10% *Dnmt3a*^R878H/+^BMT mice experienced significantly higher CEJ-ABC distance values than PBS-treated 10%*Dnmt3a*^*+*/+^BMT mice, indicating bone loss, which, importantly, was fully reversed by rapamycin (Figure 6J).

## DISCUSSION

Our study has shown that DNMT3A-driven CHIP in mice causes naturally occurring periodontal inflammation and bone loss, as well as exacerbates experimentally induced periodontitis and arthritis. These findings suggest that causality may underlie the hereby established association between *DNMT3A-*CHIP mutations and periodontitis and perhaps also the previously proposed association of CHIP and inflammatory arthritides.^79–82^ CHIP is driven mostly by *DNMT3A* mutations and represents a significant public health risk with progressively increased prevalence in adults over 60,^7,83^ when the severity and prevalence of chronic inflammatory diseases increase significantly.^84,85^

Through depletion/intervention experiments, we causally implicated Tregs, IL-17, and neutrophils, a downstream cellular effector of IL-17,^86^ in DNMT3A-driven inflammatory bone loss. IL-17 neutralization or neutrophil depletion diminished periodontal inflammation and bone loss in 10%*Dnmt3a*^R878H/+^BMT mice to levels comparable to those seen in mice that received exclusively WT BM cells. Moreover, given that 10%*Dnmt3a*^R878H/+^BMT mice contain dysfunctional Tregs that fail to restrain Th17 expansion, Treg depletion affected inflammatory bone loss selectively in the WT setting. The elevated Th17/Treg ratio in the periodontal tissue of 10%*Dnmt3a*^R878H/+^BMT mice is in line with clinical observations that human carriers of *DNMT3A* CHIP-driver mutations exhibit significantly elevated Th17/Treg ratio in peripheral blood.^16^ Our study overall shows that different hematopoietic cell types, which are produced downstream of *DNMT3A*-mutant hematopoietic progenitors, are affected in ways that aggravate bone loss.

DNMT3A-driven CHIP exacerbated periodontitis not only by enhancing inflammatory osteoclastogenesis but also by negatively impacting the host’s capacity to resolve the periodontal lesion. Indeed, the R878H mutation compromised the ability of efferocytic macrophages to express key pro-resolving mediators, including *Tgfb1*. The diminished capacity of *Dnmt3a*^*R878H/+*^ macrophages to release TGF-β1 protein upon efferocytosis can be attributed directly to the defective enzymatic activity of mutant DNMT3A. In efferocytic macrophages, DNMT3A normally methylates and epigenetically represses the phosphatase DUSP4, thereby leading to prolonged extracellular signal-regulated kinase (ERK)1/2 activation and induction of *Tgfb1* expression.^87^ Therefore, dysregulated efferocytosis signaling associated with the presence of mutant DNMT3A leads to reduced release of TGF-β1, which would otherwise contribute to inflammation resolution and bone regeneration.^88^

Our WGBS analysis revealed common hypomethylation phenotypes in CD11b^+^ and CD4^+^ cells from *Dnmt3a*^R878H/+^ mice, consistent with a human WGBS study that examined DNA methylation in peripheral blood cells of patients with germline *DNMT3A* mutations.^89^ Their analysis revealed a focal canonical hypomethylation phenotype, which was most severe with the dominant negative *DNMT3A*^R882H^ mutation. The authors concluded that the affected genes were dysregulated by mechanisms that were not specific to lineage or cell type.^89^ Collectively, these previous findings and our present data suggest that the origin of these changes in mature leukocytes lies in their common hematopoietic progenitors.

Our findings that DNMT3A-driven CHIP increases the severity of experimental periodontitis and arthritis and earlier mouse studies linking CHIP and cardiometabolic disorders^9,39,90^ suggest that CHIP may be a common mechanistic basis for inflammatory comorbidities in old age. Aging is considered a non-modifiable risk factor for periodontitis and comorbid chronic diseases.^91^ Our data, however, suggest that the effects of aging on periodontitis (and comorbidities) could be mitigated by targeting CHIP. Screening for CHIP may identify individuals with increased risk for severe periodontitis and comorbidities, including arthritis and cardiovascular disease. These individuals may benefit from therapeutic interventions aiming to block the aberrant expansion of CHIP clones. Specific inflammatory cytokines and mTOR signaling have been implicated in the selective expansion of hematopoietic stem cell (HSC) clones with CHIP-driver mutations^75,92–94^ (and this study). Enhanced mTOR expression and signaling is evidently a consequence of DNA hypomethylation in DNMT3A-driven CHIP^75^ (and this study). Therefore, CHIP may be a reversible process if treated with inhibitors capable of interfering with the fitness advantage of CHIP-mutant clones. As a proof of concept, we have shown here that systemic treatment of 10%*Dnmt3a*^R878H/+^BMT mice with an mTOR inhibitor (rapamycin) inhibits the clonal expansion of *Dnmt3a*^R878H/+^ hematopoietic cells and the development of periodontal inflammation and bone loss.

Rapamycin, an FDA-approved drug for transplantation indications, has been shown in experimental models to protect against aging-related pathologies.^95^ When administered orally to 20-month-old mice via the diet, rapamycin conferred protection against naturally occurring periodontal inflammation and bone loss.^96^ Here, systemic rapamycin did not have a significant effect on the bone levels of normal (10%*Dnmt3a*^+/+^BMT) mice. However, there are important differences in the experimental designs of the two studies, including the age (20- to 22-week-old vs. 20-month-old mice) and route of rapamycin administration (systemic vs. oral). In old mice studied by An et al.,^96^ rapamycin could in principle inhibit inflammatory bone loss by suppressing the senescence-associated secretory phenotype that fuels aging-associated inflammation and/or by promoting clearance of senescent cells.^97^ The protective effect of rapamycin on naturally occurring and experimentally induced bone loss in this study could likely be attributed to the drug’s ability to restrain DNMT3A-driven CHIP and thereby limit the infiltration of hyper-inflammatory *Dnmt3a*-mutant leukocytes into the periodontium.

The epigenetic, transcriptomic, and phenotypic alterations in HSPCs and their mature progeny resulting from loss-of-function CHIP mutations affecting epigenetic modifiers^9,39,98–100^ have mechanistic parallels with trained immunity.^3,101^ In trained immunity, exposure to infectious or inflammatory stimuli induces epigenetic rewiring in hematopoietic progenitors that is propagated to differentiated myeloid cells, which acquire the capacity to respond more robustly to future challenges, leading to protection against infections and tumors.^102–105^ However, trained immunity may be maladaptive when triggered by a chronic inflammatory condition and may lead to the exacerbation of another, comorbid inflammatory disease.^106–108^ This maladaptive epigenetic rewiring associated with trained immunity can be long-lasting (for months) but is, in principle, reversible. On the other hand, an epigenetic state shaped by CHIP mutations on HSPCs and their leukocyte progeny drives a permanent state of maladaptive inflammation and hence mimics a “fixed” type of maladaptive trained immunity.

In conclusion, DNMT3A-driven CHIP integrates aging-related alterations in HSPCs and mature progeny with distinct inflammatory comorbidities. Our study further suggests that rapamycin may represent an effective intervention to mitigate the fitness advantage of DNMT3A-CHIP-mutant clones and suppress their impact on chronic inflammatory disease.

### Limitations of the study

Our findings that CHIP due to *DNMT3A* mutations is associated with a higher prevalence and severity of clinically ascertained periodontal disease lack causality and directionality. However, a causal relationship is plausible given that the expansion of *Dnmt3a*^R878H/+^ hematopoietic cells in mice caused naturally occurring periodontitis and exacerbated experimentally induced periodontitis. Yet, we cannot exclude a bidirectional association between CHIP and periodontitis. This is because periodontitis-associated systemic inflammation could, at least in principle, exacerbate CHIP (by fueling the expansion of mutant clones or even influencing the acquisition of somatic mutations in HSPCs^93,109^), thereby creating a vicious cycle linking CHIP and the disease. In our study, we modeled DNMT3A-driven CHIP using mice harboring a heterozygous mutation (R878H) in hematopoietic cells that is equivalent to the “hotspot” heterozygous mutation R882H in humans.^19^ Future studies in mice could investigate additional *Dnmt3a* mutations and whether their effects differ from those of the R878H mutation. Although through depletion/intervention experiments we confirmed the causal involvement in inflammatory bone loss of neutrophils, Tregs, and the IL-17 pathway, our bioinformatics analyses suggest that multiple cell types are probably affected by the R878H mutation. Thus, it is uncertain which population contributes the most to the observed disease phenotype. Although treatment of mice with rapamycin blocked the aberrant expansion of *Dnmt3a*-mutant clones and their adverse impact on inflammatory bone loss, we cannot safely extrapolate these preclinical findings to human *DNMT3A*-CHIP carriers. This possibility can be tested in clinical trials. Potential success is supported by findings that rapamycin suppresses the proliferation of mTOR-over-expressing human leukemic cell lines harboring *DNMT3A* mutations, including at the R882 hotspot,^75^ based on which our preclinical model was developed.

## STAR★METHODS

### RESOURCE AVAILABILITY

#### Lead contact

Further information and requests for resources and reagents should be directed to and will be fulfilled by the lead contact, George Hajishengallis (geoh@upenn.edu).

#### Materials availability

This study did not generate any unique reagents.

#### Data and code availability

- As of the date of publication, all raw read data (FASTQ files) are available in the NCBI Short-Read Archive (SRA) database (https://www.ncbi.nlm.nih.gov/sra) under the Bio-Project ID PRJNA1002611, and WGBS data are available at the Gene Expression Omnibus database (http://www.ncbi.nlm.nih.gov/geo/) under the accession number GSE255947. Any other data reported in this paper will be shared by the lead contact upon request.
- No original code was generated in this study.
- Any additional information required to reanalyze the data reported in this paper is available from the lead contact upon request.

### EXPERIMENTAL MODEL AND STUDY PARTIPANT DETAILS

#### The Dental ARIC study cohort

The Atherosclerosis Risk in Communities (ARIC) study is a prospective cohort of cardiovascular disease risk factors and endpoints that enrolled approximately 16,000 community-dwelling adults in 4 US communities between 1987 and 1989.^115^ During the 4^th^ study visit between 1996 and 1998, an ancillary dental study (Dental ARIC) was carried out among a subset of approximately 6,000 52–74-year-old dentate participants.^116^ Dental ARIC received approval by the UNC-Chapel Hill IRB under #94-0790. The analysis of existing, deidentified genotype and human periodontal data, which have been deposited on the database of Genotypes and Phenotypes (dbGaP): https://www.ncbi.nlm.nih.gov/projects/gap/cgi-bin/study.cgi?study_id=phs000280.v6.p1, was approved as nonhuman subjects research (NHSR) by the UNC-Chapel Hill (IRB, #11-1035 and #20-1553) and CHIP-related work was reviewed and approved by the ARIC study publications committee (#3131).

#### Human clinical data

As part of Dental ARIC, five trained clinical examiners conducted comprehensive oral examinations that recorded missing teeth and clinical measures of periodontal health in the entire dentition. For the present study, we used quantitative measures of periodontal health including the number of remaining teeth, interproximal clinical attachment loss (CAL), probing depth (PD), and gingival index (GI),^117^ as well as person-level periodontitis diagnoses (Stage I/II, III, and IV) according to the recent World Workshop 2017 (WW17) classification.^35^ Additional sociodemographic, medical, and lifestyle data for Dental ARIC participants were available including age (measured in years), sex (male/female), race (European American or African American), current smoking status (yes/no), diabetes diagnosis or treatment (yes/no), body mass index (BMI) category (normal/underweight, overweight, obese).

#### Ascertainment of CHIP carriage status in ARIC

CHIP was previously determined using whole exome sequencing data using GATK Mutect2^118^ and ANNOVAR^119^ as reported by Bick et al.^6^ and Uddin et al.^120^ Consistent with previous investigations, CHIP was defined as the presence of pathogenic somatic mutations with variant allele frequency (VAF) >2% in specific hematologic cancer-driver genes (e.g., *DNMT3A*, *TET2*, *ASXL1*, *JAK2*) among participants without hematologic malignancy or clonal disease. CHIP data were generated using DNA that was collected in ARIC visits 1–4, prior to the conduct of the dental ancillary study.

#### Mice

WT C57BL/6 and congenic C57BL/6.SJL CD45.1^+^ mice (B6.SJL-*Ptprc*^*a*^*Pepc*^*b*^/BoyJ), as well as B6(Cg)-*Dnmt3a*^tm1Trow^/J (referred to as *Dnmt3a*^fl*-*R878H/+^ mice) and B6.Cg-*Commd10*^Tg(Vav1-icre)A2Kio^/J (*Vav1-iCre* mice) were purchased from the Jackson Laboratory. Loberg et al. modelled DNMT3A-driven clonal hematopoiesis in mice engineered to express the heterozygous R878H mutation, which resulted in pronounced expansion of mutant HSPC.^18^ The mice in this previous work were generated by crossing *Dnmt3a*^fl-R878H/+^ mice to *Mx1-Cre* mice and expression of the *Dnmt3a*^R878H^ mutant allele was induced with polyinosinic-polycytidylic acid (pIpC).^18^ Systemic administration of pIpC induces type I interferons,^121,122^ which in turn can induce activation and/or innate immune training in hematopoietic progenitors,^104^ thereby potentially confounding CHIP-driven effects in our inflammatory disease models. To prevent this potential issue, we crossed *Dnmt3a*^fl-R878H/+^ mice to *Vav1-iCre mice* and generated Cre^+^*Dnmt3a*^R878H/+^ mice with constitutive expression of the iCre recombinase (an optimized variant of Cre) and hematopoietic-specific expression of the heterozygous *Dnmt3a*^R878H^ mutation (referred to as *Dnmt3a*^R878H/+^ mice). Cre^+^*Dnmt3a*^+/+^ or Cre^−^*Dnmt3a*^fl-R878H/+^ (collectively referred to as Dnmt3a^+/+^) mice were used as controls for BMT. In addition, we generated *Dnmt3a*^R878H/+^GFP^+^ mice (as well as *Dnmt3a*^+/+^GFP^+^ controls) by using the UBC-GFP transgenic mice^71,72^(Jackson Labs) as well. Mice were maintained in individually ventilated cages under specific pathogen-free conditions on a standard 12-h light/dark cycle. Food and water were provided ad libitum. Sex- and age-matched mice were used for experiments at 8–10 weeks of age. Because there were no significant differences in the results obtained with male and female mice, the respective data were pooled per treatment group. Animal experiments were approved by the Institutional Animal Care and Use Committee (IACUC) of the University of Pennsylvania and were performed in compliance with institutional, state, and federal policies.

#### Ligature-induced periodontitis and bone measurements

Ligature-induced periodontitis (LIP) in mice simulates severe human periodontitis by generating a local biofilm-retentive milieu leading to dysbiotic inflammation and pronounced bone loss via mechanisms that were validated in humans.^43,49,123–128^ LIP induces bone loss in conventional (but not germ-free) mice.^126,129^ To induce LIP, a 5–0 silk ligature was tied around the left maxillary second molar tooth for 5 days while keeping the contralateral tooth (right maxillary second molar) unligated to serve as baseline control.^130^

Defleshed maxillae were used to measure bone heights, that is, the distances from the cementoenamel junction (CEJ) to the alveolar bone crest (ABC). Measurements were at 6 predetermined points involving the ligated molar and affected adjacent regions as well as the corresponding points of the unligated contralateral molar, by means of a SMZ800 stereoscope with digital imaging measurement system (Nikon). To calculate bone loss in LIP experiments, the six-site total CEJ–ABC distance for the ligated site of each mouse was subtracted from the six-site total CEJ–ABC distance of the contralateral unligated site. The results were presented in millimeters, and negative values indicate bone loss relative to the baseline (unligated control). For naturally occurring bone loss (in experimental and control groups that were not ligated), the distance between the CEJ and the ABC was measured at 6 predetermined points in both sides of the maxilla. The results were presented as total CEJ–ABC distance in experimental mice as compared to that of the controls. In ‘10% *Dnmt3a*^R878H/+^ BMT’ mice, which develop natural bone loss (Figure 1D), the ligature-induced bone loss was calculated against the unligated site that only had natural bone loss (0 baseline; Figure 2A). This normalization allows a meaningful and fair comparison with ligature-induced bone loss in ‘10% Dnmt3a^+/+^ BMT’ controls (in other words, the natural bone loss in ‘10% *Dnmt3a*^R878H/+^ BMT’ mice does not confound the comparison). To enable resolution of periodontitis, which leads to bone regeneration,^47^ in some experiments the ligatures were removed after 10 days and the mice were sacrificed 5 days later (day 15). Periodontal bone measurements were performed as described above and the CEJ–ABC distance data were further transformed to indicate bone gain (or loss, if negative value) relative to the bone levels of control mice that were sacrificed at day 10, as we previously described.^47^

#### Collagen antibody-induced arthritis

Collagen antibody-induced arthritis (CAIA) was induced in mice by i.v. (retro-orbital) injection of 1.5 mg arthritogenic monoclonal antibodies (5-clone collagen antibody cocktail; Chondrex).^131,132^ Three days later, mice were injected i.p. with 50 μg of LPS. Clinical symptoms of arthritis were daily evaluated visually for each paw using a semiquantitative scoring system graded on a scale of 0–4 per paw by a blinded procedure: 0 for normal; 1 for mild redness, slight swelling of ankle or wrist; 2 for moderate swelling of ankle or wrist; 3 for severe swelling, including some digits, ankle and foot; 4 for maximally inflamed joint. The clinical score for each mouse was the sum of the 4 paw scores for a maximum score of 16. Hind ankle joint thickness was measured by using a Käfer pocket dial thickness gauge J 15.

#### Tissue processing and cell preparations

For BM single-cell suspension preparation, mouse femoral bones were flushed with ice-cold PBS (Gibco) supplemented with 5% FBS (Gibco). Cells were forced through 70-μm nylon cell strainer to get single-cell suspension for further flow cytometric analysis. To collect BM extracellular fluid, mice femurs were flushed with 500 μl ice-cold PBS (Gibco) and the supernatant was harvested after centrifugation at 300 x g for 5 min at 4°C. To prepare mouse BM-derived macrophages (BMDM), upon lysis of erythrocytes with RBC lysis buffer (eBioscience), BM cells were plated and cultured in the presence of recombinant murine granulocyte macrophage colony-stimulating factor (GM-CSF; 20 ng/ml, R&D Systems). Culture medium was replaced every two days, and after seven days, differentiated BMDM were used in efferocytosis experiments.^49^ To isolate myeloid cells from the spleen, splenocytes were incubated with biotinylated anti-mouse CD11b antibody (clone M1/70; Biolegend) followed by anti-biotin microbeads from Miltenyi Biotec. Myeloid cells were positively selected using LS columns on the magnetic field of QuadroMACS separator according to the manufacturer’s instructions (Miltenyi Biotec). For isolating splenic T cells, myeloid cells were first removed by negative selection for CD11b^+^ cells and then T cells were obtained by positive selection for CD4^+^ (clone GK1.5; Biolegend) cells. To isolate splenic neutrophils, splenocytes were incubated with biotinylated anti-mouse Ly6G antibody (clone 1A8; Biolegend) followed by anti-biotin microbeads from Miltenyi Biotec. Neutrophils were positively selected using LS columns on the magnetic field of QuadroMACS Separator according to the manufacturer’s instructions (Miltenyi Biotec). For isolating splenic monocytes, neutrophils were first removed by negative selection for Ly6G^+^ cells and then monocytes were obtained by positive selection for Ly6C^+^ (clone HK1.4; Biolegend) cells as we previously described.^104^ Neutrophils were also isolated from the peripheral blood by using EasySep^™^ Mouse Neutrophil Enrichment Kit according to the manufacturer’s directions (STEMCELL). Gingival tissues around the area of ligature placement (and the contralateral control area) were harvested on day 5 for analysis. On day 5, gingiva was dissected around the area of ligature placement and digested for 1h at 37°C with RPMI 1640 medium (Gibco) supplemented with collagenase IV (3.2 mg/ml, Worthington) and DNase (0.15 μg/ml, Roche).^133^ Single-cell suspensions were obtained by mashing the tissue against a strainer using plungers and filtered for staining and flow cytometry. Synovial tissues from joints were digested with 2 mg/mL collagenase type IV (Worthington) and 0.1 mg/mL DNase I (Roche) in DMEM containing 10% fetal bovine serum and penicillin/streptomycin for 30 min at 37 °C. Single-cell suspensions were obtained by mashing the tissue against a strainer using plungers and filtered for staining and flow cytometry.

#### Bone marrow transplantation

BM cells were isolated from C57BL/6 (CD45.2) *Dnmt3a*^R878H/+^ mice and Dnmt3a^+/+^ littermates, as well as from congenic C57BL/6.SJL (CD45.1) mice. As indicated in Figure 1A, in the experimental group, lethally irradiated (9.5 Gy) CD45.1 mice received 10% *Dnmt3a*^R878H/+^ CD45.2 BM cells and 90% WT CD45.1 BM cells (2×10^5^ *Dnmt3a*^R878H/+^ CD45.2 BM cells and 18×10^5^ WT CD45.1 BM cells; total of 2×10^6^ BM cells/recipient mouse). The experimental group was designated ‘10%*Dnmt3a*^R878H/+^BMT’. In the control group , lethally irradiated (9.5 Gy) CD45.1 mice received 10% Dnmt3a^+/+^ CD45.2 BM cells and 90% WT CD45.1 cells (2×10^5^ Dnmt3a^+/+^ CD45.2 BM cells & 18×10^5^ WT CD45.1 BM cells, thus exclusively DNMT3A-sufficient cells; total of 2×10^6^ BM cells/recipient mouse). The control group was designated ‘10%*Dnmt3a*^+/+^BMT’. At 12 weeks post-BMT, ‘10%*Dnmt3a*^R878H/+^BMT’ and ‘10% *Dnmt3a*^+/+^BMT’ recipient mice were subjected, or not, to LIP for 5 days^43,130^ or to CAIA for 14 days.^131,132^

#### Cell depletion and intervention experiments

To deplete neutrophils *in vivo* (in certain LIP experiments), mice were i.p. injected with 0.4 mg of anti-Ly6G (Clone 1A8; Cat# BE0075-1, BioXCell) or rat IgG2a isotype control (Clone 2A3; Cat# BE0089, BioXCell).^43,134^ To deplete Tregs, mice were i.p. injected with 0.5 mg of anti-GITR (Clone DTA-1; Cat# BE0063, BioXCell) or rat IgG2b isotype control (Clone LTF-2; Cat# BE0090, BioXCell). In both cases, treatment was administered 1 day before and 2 days after placing the ligatures (2 total i.p. injections).^135,136^ Depletion efficiency was determined in peripheral blood 5 days post-LIP. Anti-Ly6G effectively eliminated (by 99±0.7 %, *n*=10 mice) neutrophils (CD11b^+^Ly6G^+^), whereas anti-GITR reduced Tregs (CD4^+^CD25^+^FOXP3^+^) by 42% (±13.8; *n*=10 mice), consistent with earlier observations.^134,136^ To neutralize endogenous IL-17, mice were locally microinjected with anti-IL-17A (Clone 17F3; BioXCell) or mouse IgG1 isotype control (Clone MOPC-21; BioXCell) at 10 μg /dose, 1 day before and on days 1 and 3 after ligature placement, for a total of three doses.^41^

#### Treatment of transplanted mice with rapamycin

Two weeks post-BMT, groups of ‘10%*Dnmt3a*^R878H/+^BMT’ and ‘10%*Dnmt3a*^+/+^BMT’ mice were administered by i.p. injection of either PBS (vehicle control; containing 0.69% DMSO to match the concentration of this solvent in the rapamycin preparation) or rapamycin (LC laboratories) at 4 mg/kg mouse^75^ twice per week for 4 weeks, followed by the same dose once a week until sacrifice (*i.e.*, for another 6 weeks).

### METHOD DETAILS

#### Quantitative real-time PCR (qPCR)

Total cellular RNA was isolated from mouse gingiva using the GeneJET RNA Purification Kit (Thermo Fisher Scientific). For real-time PCR, total RNA was reverse-transcribed using High-Capacity RNA-to-cDNA Kit (Applied Biosystems) and real-time PCR with cDNA was performed using the Applied Biosystems 7500 Fast Real-Time PCR System, according to the manufacturer’s protocol (Applied Biosystems). TaqMan probes and gene-specific primers for detection and quantification of murine genes investigated in this study were purchased from Thermo Fisher Scientific: *Gapdh*, Mm99999915_g1; *Il6*, Mm00446190_m1; *Il17a*, Mm00439618_m1; *Il1b*, Mm00434228_m1; *Il23a*, Mm00518984_m1; *Tnf*, Mm00443258_m1; *Tnfsf11*, Mm00441906_m1; *Tnfrsf11a*, Mm00437132_m1; *Tnfrsf11b*, Mm00435454_m1; *Tgfb1*, Mm01178820_m1; *Mtor*, Mm00444968_m1; *Pcna*, Mm05873628_g1; *Eif4ebp1*, Mm04207378_g1; *Eif4ebp2*, Mm00515675_m1; *Eif4ebp3*, Mm01406408_m1; *Hif1a*, Mm00468869_m1; *Ccnd1*, Mm00432359_m1; *Nr1h2* (*Lxrb*), Mm00437265_g1; *Nr1h3* (*Lxra*), Mm00443451_m1; *Rxra*, Mm00441185_m1; *Abca1*, Mm00442646_m1; *Tgm2*, Mm00436979_m1; *Cd36*, Mm00432403_m1; *Ucp2*, Mm00627599_m1; *Axl*, Mm00437221_m1. Data were analyzed using the comparative (DDCt) method and were normalized to *Gapdh* mRNA and are presented in figures as fold change relative to control, which was assigned an average value of 1.

#### Flow cytometry and sorting

Flow cytometric analysis was performed on a NovoCyte flow cytometer (ACEA Biosciences). For cell surface phenotypic analysis, a lineage (Lin) cocktail, including monoclonal antibodies against the following molecules (antibody clone) was used: CD3e (145-2C11), CD11b (M1/70), Gr1 (RB6-8C5), B220 (RA3-6B2) and TER119 (TER-119). Other antibody reagents used in experiments included monoclonal antibodies against Sca1 (E13-161.7), cKit (2B8), CD45.1 (A20), CD45.2 (104), CD3 (17A2), CD19 (6D5), CD11b (M1/70), Ly6C (HK1.4), Ly6G (1A8), I-A/I-E (M5/114.15.2), CX3CR1 (SA011F11), CD45 (30-F11), F4/80 (BM8), FOXP3 (FJK-16s), IL-17A (TC11-18H10.1), CTLA4 (UC10-4B9), ICOS (7E.17G9), neuropilin-1 (3E12), GITR (DTA-1), CD25 (3C7), IL-17F (9D3.1C8), RORγt (Q31-378) and IL-23R (12B2B64). Data were analyzed with NovoExpress software (ACEA Biosciences). For FACS analysis or sorting, different cell types investigated were defined as follows: LSK, Lin^−^Sca-1^+^cKit^+^; neutrophils, live CD45^+^CD11b^+^Ly6G^+^; monocytes, live CD45^+^CD11b^+^Ly6G^−^Ly6C^+^; Treg, live CD45^+^CD3^+^CD4^+^FOXP3^+^; Th17, live CD45^+^CD3^+^CD4^+^IL-17A^+^; osteoclastogenic macrophages, live CD45^+^Ly6C^int^I-A^+^/I-E^+^CX3CR1^hi^F4/80^+^; BM osteoclast precursors, live CD11b^−/low^ Ly6C^hi^. The percentage of specific CD45.2^+^ cell types was calculated with regard to the total CD45^+^ (CD45.1^+^ plus CD45.2^+^) population of that cell type. Cell sorting was performed on a FACSARIA^™^ instrument (Becton, Dickinson Immunocytometry Systems, USA).

#### ROS induction and measurement

Peripheral blood neutrophils were stimulated with 10^−7^ M PMA for 10 min at 37°C and stained using the CellROX Green flow cytometry assay kit according to manufacturer’s instructions (Thermo Fisher). The cells were then washed and ROS production was analyzed by flow cytometry. Mean fluorescence intensity (MFI) was calculated.

#### Chemotaxis assay

The chemotaxis of neutrophils towards 10 ng/ml of CXCL1 or CXCL2, as well as the chemotaxis of monocytes to 20 ng/ml MCP-1, were tested using a Transwell system with 5-μm pores (Corning) according to a previously described protocol.^137^ The number of migrated cells in the absence of chemokine was assigned an average value of 1 and the experimental results were expressed as a ratio (chemotactic index) relative to this value.

#### Cytokine measurements

For *ex vivo* stimulation of splenic monocytes and neutrophils with LPS, isolated cells were seeded into 96-wells plates and stimulated with 10 ng/ml of *E. coli* O111:B4 LPS (InVivogen) for 24h. The culture supernatants were collected for measuring IL-1β, IL-6, and TNF concentrations using mouse ELISA kits, according to the manufacturer’s instructions (Invitrogen). ELISA was also used to measure cytokine concentrations in the BM extracellular fluid and the serum.

#### *Ex vivo* mouse T cell differentiation, restimulation and suppressive assay

All cytokines and antibodies used in these experiments were from BioLegend. Naive splenic CD4^+^ T cells were isolated using EasySep Mouse Naı¨ve CD4^+^ T Cell isolation kit (STEMCELL). The purity of the isolated CD4^+^ T cells was ~95%. The cells were activated by plate-bound anti-CD3 (clone 145-2C11; 2 μg/ml) and anti-CD28 (clone 37.51; 1 μg/ml) in the presence of different sets of cytokines in complete RPMI 1640 (Gibco), supplemented with 10% FBS (Atlanta Biologicals), 1% penicillin/streptomycin (Thermo-Fisher), and 50 μM 2-mercaptoethanol (Thermo-Fisher). For induction of Treg differentiation (iTregs), CD4^+^ T cells were incubated with TGFb1 (5 ng/ml) and IL-2 (40 ng/ml) for three days. For Th17 polarization under ‘non-pathogenic’ conditions,^70^ CD4^+^ T cells were incubated with IL-6 (50 ng/ml) and TGFb1 (1 ng/ml) for three days. For Th17 polarization under ‘pathogenic conditions’^70^, CD4^+^ T cells were incubated with IL-6 (50 ng/ml), TGFb1 (1 ng/ml), IL-1β (10 ng/ml) and IL-23 (10 ng/ml) for three days. Using EasySep Mouse CD4^+^CD25^+^ Regulatory T Cell Isolation Kit II (STEMCELL), CD4^+^CD25^+^ cells were sorted to high purity (>95%) from Treg induction cultures. For Treg suppression assay, CD4^+^CD25^−^ T cells were isolated using CD4 positive selection and CD25 negative selection (EasySep^™^ Mouse CD4^+^CD25^+^ Regulatory T Cell Isolation Kit II). The cells (90–95% pure) were labeled with CFSE (5 μM; Invitrogen) and plated at 2.5×10^4^ cells/well. Flow cytometry analyzing CFSE dilution was performed by gating on CD4^+^CFSE^+^ cells, stimulated for 72 hrs with anti-CD3 (2 μg/ml) and anti-CD28 (1 μg/ml), which were cultured alone or with purified Dnmt3a^+/+^ iTregs or *Dnmt3a*^R878H/+^ iTregs.

#### Histology

Knee joints were collected and fixed in 4% paraformaldehyde for 3 days, followed by decalcification in formic acid for 2 to 3 weeks. The tissues were immersed stepwise in 10%, 20% and 30% sucrose in PBS. The tissues were embedded in Optimal Cutting Temperature (OCT) media and 10-mm-thick sections were prepared, which were mounted on SuperFrost Plus slides. Pathology of joints was evaluated by hematoxylin and eosin (H&E) staining and observation by bright-field microscopy. Cartilage erosion was evaluated by Safranin-O and Fast Green staining according to the manufacturer’s protocol (ScienCell). Mouse maxillae with intact surrounding tissue were fixed in 4% paraformaldehyde for 24h at 4°C, decalcified in formic acid for 2 weeks, followed by immersing overnight in 30% sucrose in PBS and then embedded in optimal cutting temperature compound. TRAP staining of coronal tissue sections (8-μm thick) was performed using the TRAP staining kit, as instructed by the manufacturer (Cosmo Bio USA). Images were captured using a Nikon Eclipse Ni-E microscope. TRAP-positive multinucleated cells (more than three nuclei) were considered to be osteoclasts.

#### Osteoclastogenesis

RANKL-induced osteoclastogenesis was performed according to a standard protocol^138^ using mouse BM osteoclast precursor cells. BM cells were flushed from femurs and tibias of mice. After lysis of erythrocytes using RBC lysis buffer (eBioscience), CD11b^−/lo^Ly6C^hi^ OCPs were sorted using a FACSARIA^™^ instrument (Becton Dickinson Immunocytometry Systems, USA). The sorted cells (25 × 10^3^/well in a 96-well plate) were cultured in DMEM media/10% FBS with 100 ng/ml M-CSF and 100 ng/ml soluble recombinant RANKL (R&D Systems) for 5 days to generate osteoclasts. The cells were fixed and stained for TRAP using an TRAP staining kit (Cosmo Bio USA) and TRAP-positive multinucleated (≥ 3 nuclei) cells were counted.^138^

#### Efferocytosis

BMDM from *Dnmt3a*^*+/+*^ or *Dnmt3a*^*R878H/+*^ mice were co-cultured for 3h with apoptotic neutrophils (at a 5:1 ratio of apoptotic neutrophils to BMDM) in the presence of the efferocytosis opsonin DEL-1-Fc (500 ng/ml).^49^ The neutrophils were rendered apoptotic upon overnight culture in HBSS with 1% FBS. TGFb1 protein levels in culture supernatants were measured by ELISA. For gene expression analysis, BMDM were washed three times with PBS prior to RNA isolation.^49^

#### Microcomputed tomography (microCT)

After fixation in 10% formalin, the femur bone and 2^nd^ lumbar vertebra were scanned at the Penn Center for Musculoskeletal Disorders (PCMD) using a mCT 45 (Scanco Medical AG, Brüttisellen, Switzerland) with an isotropic voxel size of 7.4 μm, x-ray energy of 55 kVp and 400 ms integration time. For femur bone and vertebra, a 50-slice-thick region of interest was analyzed with Gaussian noise filter (sigma=1.2, support=2.0) and global threshold (364 mg HA/cm^3^). A 3D standard microstructural analysis of trabecular bone was performed to quantify bone volume fraction (BV/TV), trabecular number (Tb.N), thickness (Tb.Th), and separation (Tb.Sp). For cortical bone analysis, cortical bone area (Ct.Ar), total cross-sectional area (Tt.Ar), cortical area fraction (Ct.Ar/Tt.Ar), cortical thickness (Ct.Th) were quantified. 3D reconstruction and structural parameters quantification were performed using Scanco Medical software.^139^

#### Single-cell RNA sequencing

At 12 weeks post-BMT, ‘10%*Dnmt3a*^R878H/+^BMT’ and ‘10%*Dnmt3a*^+/+^BMT’ mice were subjected to LIP for 5 days or CAIA for 7 days. Dissected gingival and synovial tissues were pooled from 5 and 3 mice, respectively, and processed for sorting (by means of FACSAria cell sorter) of CD11b^+^ and CD3^+^ cells, which were further sorted into CD45.1^+^ and CD45.2^+^ cells. 60,000 CD45.2^+^CD11b^+^ or CD45.1^+^ CD11b^+^ cells and 8,000 CD45.2^+^CD3^+^ or CD45.1^+^CD3^+^ cells from each gingiva or synovium were mixed respectively. The cells were resuspended in 1X PBS containing 0.04% BSA (400 μg/ml) and processed with Chromium Next GEM Single Cell 3^0^ Kit v3.1, 16 rxns (10x Genomics) at the CAG Sequencing Core of Children’s Hospital of Philadelphia, PA, USA. Cell suspensions contained more than 99% viable cells, as determined by microscopy. The cell numbers were adjusted to 700–1200 cells per μl and added to 10x Chromium RT mix to achieve loading target numbers of around 20,000 cells. cDNA synthesis was performed per the manufacturer’s instructions, and library preparation and sequencing (Novaseq SP reagent kit; 100 cycles) were performed using the Novaseq 6000 platform (Illumina) per the manufacturer’s instructions.

#### Whole-genome bisulfite sequencing

Splenic CD11b^+^ and CD4^+^ cells were isolated from Dnmt3a^+/+^ or *Dnmt3a*^R878/+^ mice using LS columns on the magnetic field of QuadroMACS Separator according to the manufacturer’s instructions (Miltenyi Biotec). High-molecular-weight DNA was isolated using MagAttract HMW DNA Kit according to the manufacturer’s instructions (Qiagen). Whole-genome bisulfite sequencing was performed using the Zymo-Seq WGBS Library Kit (Zymo research, D5465) according to the manufacturer’s recommendations at the High Throughput Sequencing Core of Children’s Hospital of Philadelphia, PA, USA. The sequence was generated on Illumina NovaSeq 6000 using NovaSeq 6000 S4 Reagent Kit v1.5 (200 cycles) at 40X coverage.

### QUANTIFICATION AND STATISTICAL ANALYSIS

#### Single-cell RNA sequencing analysis

Cell Ranger 6.1.2 (https://support.10xgenomics.com/single-cell-gene-expression/software/downloads/6.1) was used to process raw sequencing results.^110^ Briefly, Cell Ranger mkfastq pipeline was used to demultiplex sample index reads to generate FASTQ files for each sequencing library, and then raw reads were aligned to the mm10 (GENCODE vM23/Ensembl 98) using STAR aligner with default parameters. Subsequently, data filtering, integration, normalization and scaling were performed using the R package Seurat version 4.3.0^111,140^ and dimensionality reduction and clustering were further done by Uniform Manifold Approximation and Projection (UMAP) analysis. Low-quality cells were filtered out using the following criteria: (i) number of detected genes of each cell was required to be larger than 500, and (ii) percentage of UMIs derived from mitochondrial genes below 10%. The UMI counts were normalized by library size factors. As a result, Cell Ranger recovered total of 69,509 cells: Gingiva: ‘10%*Dnmt3a*^R878H/+^BMT’-CD45.1, 6,745 cells; ‘10%*Dnmt3a*^R878H/+^BMT’-CD45.2, 5,303 cells; ‘10% *Dnmt3a*^+/+^BMT’-CD45.1, 9,613 cells; ‘10%*Dnmt3a*^+/+^BMT’-CD45.2, 12,674 cells; Synovium: ‘10%*Dnmt3a*^R878H/+^BMT’-CD45.1, 8,663 cells; ‘10%*Dnmt3a*^R878H/+^BMT’-CD45.2, 9,801 cells; ‘10%*Dnmt3a*^+/+^BMT’-CD45.1, 9,242 cells; and ‘10%*Dnmt3a*^+/+^ BMT’-CD45.2: 7,468 cells. Cluster analysis based on cell types was performed using Heatmapper^141^ (Distance measurement method= “Kendall’s Tau”, clustering method = “Average Linkage”). Differential expression analysis of genes was done by FindAllMarkers function from Seurat package with Wilcoxon test (min.pct = 0.1, logfc.threshold = 0.25), and indicated genes were presented by Seurat package’s DotPlot function. Gene Ontology (GO) enrichment analysis of each cluster and major cell types were performed through rbioapi^112^ by calling PANTHER (Protein ANalysis THrough Evolutionary Relationships; http://www.pantherdb.org) enrichment with Fisher test with FDR-correction, *p*<0.05. Major plots were generated by default function of Seurat (v4.3.0) and customized R scripts. Total differentially expressed genes (‘10%*Dnmt3a*^R878H/+^BMT’-CD45.2 vs. ‘10%*Dnmt3a*^+/+^BMT’-CD45.2) in the various cell types were analyzed using STRING analysis function, and KEGG pathway terms (p-values corrected for multiple testing within each category using the Benjamini–Hochberg procedure; *p*<0.05) related to periodontitis and arthritis were displayed. For insights into the differentially upregulated genes, we used the Search Tool for the Retrieval of Interacting Genes/Proteins (STRING), a database of known and predicted protein-protein interactions with functional analysis^113^(https://string-db.org). CellChat analysis was performed to infer intercellular communication (CD45.2^+^ [mutant] and CD45.1^+^ [WT] cells from ‘10%*Dnmt3a*^R878H/+^BMT’ mice) by integrating the data from scRNA-seq analysis with the ligand-receptor database CellChatDB (v2).^51,114^ Briefly, we followed the official workflow and converted our Seurat project into CellChat project and applied the preprocessing functions ‘identifyOverExpressedGenes’ and ‘identifyOverExpressedInteractions’ with default setting. We calculated the communication probability/strength with ‘thresholdedMean’ and filtered out communications for cell groups with cells less than six. We searched all four interaction databases- ‘Secreted Signaling’, ‘Cell-Cell Contact’, ‘ECM-Receptor’ and ‘Non-protein Signaling’ from CellChatDB. For the main analyses, the core functions ‘computeCommunProb’, ‘computeCommunProbPathway’ and ‘aggregateNet’ were applied using standard parameters. Finally, all related figures were drawn by available tool functions including ‘netVisual_heatmap’, ‘netVisual_diffInteraction’, ‘rankNet’ and ‘netVisual_aggregate’.

#### Whole-genome bisulfite sequencing analysis

The reads were mapped to mouse genome “mm10” using BISulfite-seq CUI Toolkit (BISCUIT), as described in https://github.com/huishenlab/Biscuit_Snakemake_Workflow. The methylation ratios at all CpGs were analyzed using the “metilene” tool to identify differentially methylated regions (DMRs).^142^ The criteria for each DMR included: spanning >10 CpGs; mean methylation difference between groups >0.2; false discovery rate (FDR) <0.05. The gene annotations were based on protein-coding genes from Ensembl release 95 and its accompanying regulatory build.

#### Statistical analysis

##### Analytical approach in human studies

We used descriptive methods to examine the distribution of any CHIP and specifically *DNMT3A* mutation carriage in the study sample, overall and across strata of social, clinical, and lifestyle characteristics. We tested bivariate associations of *DNMT3A* CHIP status with WW17 periodontitis diagnosis using chi-squared and non-parametric trend tests^143^ and with quantitative measures of periodontal health using Student’s *t*-tests, employing a conventional *p*<0.05 statistical significance criterion. To quantify crude, as well as age- and sex-adjusted differences in periodontitis (*i.e.*, Stage IV [severe] periodontitis versus Stages I/II [initial/moderate] disease) and relevant quantitative measures attributable to *DNMT3A* CHIP, we used logistic and linear regression models and marginal effects estimation^144^ and reported average marginal effects (A.M.E.) expressed in absolute changes in percentage points (p.p.) and 95% confidence intervals (CI). Analyses were done using Stata/MP version 18.0 (StataCorp LLC, Texas, US).

##### Mouse studies statistics

After testing for normality, data were analyzed by parametric or non-parametric tests. Parametric tests included two-tailed unpaired Student’s *t* test (when comparing two groups) and one-way ANOVA followed by a Tukey’s multiple comparison test (when comparing more than two groups). In a few instances where data did not follow normal distribution, the non-parametric two-tailed Mann-Whitney U-test was used for comparison of two groups, and the Kruskal-Wallis test followed by Dunn’s multiple comparisons test was used for comparison of more than two groups. Two-way repeated measures ANOVA and Sidak’s or Tukey’s multiple comparisons test were used to analyze data with multiple comparisons in repeated measures designs. All statistical analyses were performed using GraphPad Prism software (version 9; GraphPad Inc). *P* values < 0.05 were considered to be statistically significant.

## Supplementary Material

MMC1

2

## Figures and Tables

**Table 1.** Periodontal disease diagnoses, clinical measures of periodontal health, and inflammatory markers’ associations with *DNMT3A*-CHIP mutation carrier status among the dental ARIC participants

	Entire sample	*DNMT3A* mutation carriers	*DNMT3A* mutation non-carriers	*p* ^b^
	*n* (column %)	*n* (row %)	*n* (row %)	
Periodontitis diagnosis^a^	–	–	–	0.048, 0.017^c^
Stages I and II	1,912 (38.7)	36 (1.9)	1,876 (98.1)	–
Stage III	1,475 (29.8)	33 (2.2)	1,442 (97.8)	–
Stage IV	1,559 (31.5)	49 (3.1)	1,510 (96.9)	–
Interproximal CAL ≥ 3 mm				
% of sites (SD)	24.1 (24.1)	29.3 (26.4)	23.9 (24.0)	0.017
Gingival index ≥ 1				
% of sites (SD)	34.9 (41.1)	43.4 (43.2)	34.7 (41.1)	0.027
Probing depth ≥ 4 mm				
% of sites (SD)	7.4 (11.4)	8.0 (12.6)	7.4 (11.4)	0.544
Number of teeth				
Mean (SD)	21.7 (7.2)	20.6 (7.4)	21.8 (7.2)	0.085

a World Workshop 2017 classification.^35^

b Obtained from chi-squared tests.

c Non-parametric test for trend (Cuzick). CAL, clinical attachment loss; SD, standard deviation.

**Table:** KEY RESOURCES TABLE

REAGENT or RESOURCE	SOURCE	IDENTIFIER
Antibodies		
FITC anti-mouse Lineage Cocktail	Biolegend	Cat#133301
Rat anti-mouse CD16/32	Biolegend	Cat# 101302; RRID: AB_312801
Rat anti-mouse cKit (CD117)	Biolegend	Cat#105808; RRID: AB_313217
Rat anti-mouse Sca1 (Ly6-A/E)	Biolegend	Cat#122514; RRID: AB_756199
Rat anti-mouse IL-17A	Biolegend	Cat#506908; RRID: AB_536010
Mouse anti-mouse IL-17A	BioXCell	Cat# BE0173; RRID: AB_10950102
Mouse IgG1 isotype control	BioXCell	Cat# BE0083; RRID: AB_1107784
Rat anti-mouse Ly6G	BioXCell	Cat# BE0075-1; RRID: AB_1107721
Rat IgG2a isotype control	BioXCell	Cat# BE0089; RRID: AB_1107769
Rat anti-mouse GITR	BioXCell	Cat# BE0063; RRID: AB_1107688
Rat IgG2b isotype control	BioXCell	Cat# BE0090; RRID: AB_1107780
Rat anti-mouse CD45	Biolegend	Cat#103128; RRID: AB_493715
Rat anti-mouse CD19	Biolegend	Cat# 115510; RRID: AB_313645
Rat anti-mouse CD3	Biolegend	Cat#100204, 100206; RRID: AB_312661, RRID: AB_312663
Armenian Hamster anti-mouse CD3ε	Biolegend	Cat# 100340; RRID: AB_11149115
Syrian Hamster anti-mouse CD28	Biolegend	Cat# 102116; RRID: AB_11147170
Rat anti-mouse CD4	Biolegend	Cat# 100414, 100404; RRID: AB_312699, AB_312689
Mouse anti-mouse CD45.1	Biolegend	Cat# 110708, 110714; RRID: AB_313497, RRID: AB_313503
Mouse anti-mouse CD45.2	Biolegend	Cat# 109832, 109808; RRID: AB_2565511, RRID: AB_313445
Rat anti-mouse CD11b	Biolegend	Cat# 101222, 101212, 101204; RRID: AB_493705, RRID: AB_312795, AB_312787
Rat anti-mouse Ly6G	Biolegend	Cat# 127618; RRID: AB_1877261
Rat anti-mouse Ly6C	Biolegend	Cat# 128006, 128018; RRID: AB_1186135, RRID: AB_1732082
Armenian Hamster anti-mouse CTLA4	Biolegend	Cat#106310; RRID: AB_2087653
Rat anti-mouse CD278 (ICOS)	Biolegend	Cat# 117424, 117406; RRID: AB_2832419, RRID: AB_2122712
Rat anti-mouse GITR	Biolegend	Cat# 126308; RRID: AB_1089125
Rat anti-mouse CD304 (Neuropilin-1)	Biolegend	Cat# 145206; RRID: AB_2562032
Rat anti-mouse IL-23R	Biolegend	Cat#150906; RRID: AB_2687346
Mouse anti-mouse IL-17F	Biolegend	Cat# 517006; RRID: AB_10661903
Rat anti-mouse CD25	Biolegend	Cat#101908; RRID: AB_961212
Rat anti-mouse I-A/I-E	Biolegend	Cat#107614; RRID: AB_313329
Mouse anti-mouse CX3CR1	Biolegend	Cat#149020; RRID: AB_2565703
Rat anti-mouse F4/80	Biolegend	Cat#123112; RRID: AB_893482
Mouse Anti-Mouse RORγt	BD Pharmingen	Cat# 562683; RRID: AB_2737720
Rat anti-mouse FOXP3	eBioscience	Cat# 17-5773-82, 11-5773-82; RRID: AB_469457, RRID: AB_465243
Arthritogenic monoclonal antibodies (5-clone collagen antibody cocktail)	Chondrex	Cat#53100
Chemicals, peptides, and recombinant proteins		
Collagenase, Type 4	Worthington	Cat# LS004186
ACK Lysing Buffer	Gibco	Cat# A1049201
DNase I	Roche	Cat# 04536282001
LIVE/DEAD^™^ Fixable Violet Dead Cell Stain Kit	Invitrogen	Cat# L34964
Streptavidin microBeads	Miltenyi Biotec	Cat# 130-048-101
LPS-EB (LPS from *E. coliO111:B4*)	Invivogen	Cat# tlrl-eblps
DAPI (4’,6-Diamidino-2-Phenylindole,Dihydrochloride)	Thermo Fisher Scientific	Cat# D1306
Rapamycin	LC Laboratories	Cat# R-5000_1g
GeneJET RNA Purification Kit	Thermo Fisher Scientific	Cat# K0731
CellTrace^™^ CFSE Cell Proliferation Kit	Invitrogen	Cat# C34554
Recombinant Mouse TGF-β1	Biolegend	Cat# 763102
Recombinant Mouse IL-6	Biolegend	Cat# 575702
Recombinant Mouse IL-2	Biolegend	Cat# 575402
Recombinant Mouse IL-1β	Biolegend	Cat# 575102
Recombinant Mouse IL-23	Biolegend	Cat# 589002
Recombinant Mouse M-CSF	R&D Systems	Cat# 416-ML-010/CF
Recombinant Mouse GM-CSF	R&D Systems	Cat# 415-ML-010/CF
Recombinant Mouse TRANCE/RANK L/TNFSF11	R&D Systems	Cat# 462-TR-010/CF
Recombinant Mouse CXCL1/KC	R&D Systems	Cat# 453-KC-010/CF
Recombinant Mouse CXCL2/MIP-2	R&D Systems	Cat# 452-M2-010/CF
Recombinant Mouse CCL2/JE/MCP-1	R&D Systems	Cat# 479-JE-010/CF
Critical commercial assays		
IL-1β mouse ELISA kit	Invitrogen	Cat# 88-7013-88
IL-6 mouse ELISA kit	Invitrogen	Cat# 88-7064-88
IFNg mouse ELISA kit	Invitrogen	Cat#88-7314-88
TNF mouse ELISA kit	Invitrogen	Cat# 88-7324-88
TGFb1 Human/Mouse ELISA Kit	Invitrogen	Cat#88-8350-88
CellROX Green Flow Cytometry Assay Kit	Invitrogen	Cat# C10492
TRAP Staining Kit	Cosmo Bio USA	Cat# PMC-AK04F
Safranin O Staining Kit	ScienCell	Cat# 8348
High-Capacity RNA-to-cDNA Kit	Applied Biosystems	Cat# 4387406
NovaSeq 6000 SP Reagent Kit v1.5 (100 cycles)	Illumina	Cat#20028401
Chromium Next GEM Single Cell 3ʹ Kit v3.1, 16 rxns	10x Genomics	Cat# PN-1000268
Deposited data		
Single-cell RNA sequencing data	This paper	PRJNA1002611
Whole genome bisulfite sequencing data	This paper	GSE255947
Experimental models: Organisms/strains		
Mouse: C57BL/6	The Jackson Laboratory	Stock#000664
Mouse: C57BL/6-CD45.1 B6.SJL-Ptprca Pepcb/BoyJ	The Jackson Laboratory	Stock#002014
Mouse: B6(Cg)-Dnmt3atm1Trow/J	The Jackson Laboratory	Stock#032289
Mouse: B6.Cg-Commd10Tg(Vav1-icre) A2Kio/J	The Jackson Laboratory	Stock#008610
Software and algorithms		
GraphPad Prism 9	Graphpad Software	https://www.graphpad.com/
NovoExpress software	ACEA Biosciences	https://www.aceabio.com
Cell Ranger 6.1.2	Zheng et al.^110^	https://support.10xgenomics.com/single-cell-gene-expression/software/downloads/6.1
R package Seurat	Stuart et al.^111^	https://github.com/satijalab/seurat/releases/tag/v3.0.0
R package rbioapi	Rezwani et al.^112^	https://github.com/moosa-r/rbioapi/
STRING	Szklarczyk et al.^113^	https://string-db.org
CellChatDB (V2)	Jin et al.^51^ and Jin et al.^114^	https://github.com/sqjin/CellChat
BISCUIT	See link in the next cell in lieu of reference to the Snakemake workflow which processes paired-end WGBS data using BISCUIT	https://github.com/huishenlab/Biscuit_Snakemake_Workflow

## Highlights

- CHIP-*DNMT3A* mutations are associated with increased prevalence of periodontitis
- DNMT3A-driven CHIP in mice causes inflammatory bone loss
- Altered function of neutrophils and Tregs exacerbates CHIP-driven inflammation
- DNMT3A-driven CHIP and inflammatory bone loss are reversed by rapamycin

## References

[1] Jaiswal S, and Ebert BL (2019). Clonal hematopoiesis in human aging and disease. Science 366, eaan4673. 10.1126/science.aan4673. 31672865 PMC8050831

[2] Yura Y, Sano S, and Walsh K (2020). Clonal hematopoiesis: A new step linking inflammation to heart failure. JACC Basic Transl. Sci. 5, 196–207. 10.1016/j.jacbts.2019.08.006. 32140625 PMC7046537

[3] Chavakis T, Wielockx B, and Hajishengallis G (2022). Inflammatory modulation of hematopoiesis: linking trained immunity and clonal hematopoiesis with chronic disorders. Annu. Rev. Physiol. 84, 183–207. 10.1146/annurev-physiol-052521-013627. 34614373

[4] Jaiswal S, Fontanillas P, Flannick J, Manning A, Grauman PV, Mar BG, Lindsley RC, Mermel CH, Burtt N, Chavez A, (2014). Age-related clonal hematopoiesis associated with adverse outcomes. N. Engl. J. Med. 371, 2488–2498. 10.1056/NEJMoa1408617. 25426837 PMC4306669

[5] Genovese G, Kähler AK, Handsaker RE, Lindberg J, Rose SA, Bakhoum SF, Chambert K, Mick E, Neale BM, Fromer M, (2014). Clonal hematopoiesis and blood-cancer risk inferred from blood DNA sequence. N. Engl. J. Med. 371, 2477–2487. 10.1056/NEJMoa1409405. 25426838 PMC4290021

[6] Bick AG, Weinstock JS, Nandakumar SK, Fulco CP, Bao EL, Zekavat SM, Szeto MD, Liao X, Leventhal MJ, Nasser J, (2020). Inherited causes of clonal haematopoiesis in 97,691 whole genomes. Nature 586, 763–768. 10.1038/s41586-020-2819-2. 33057201 PMC7944936

[7] Steensma DP (2018). Clinical implications of clonal hematopoiesis. Mayo Clin. Proc. 93, 1122–1130. 10.1016/j.mayocp.2018.04.002. 30078412

[8] Coombs CC, Zehir A, Devlin SM, Kishtagari A, Syed A, Jonsson P, Hyman DM, Solit DB, Robson ME, Baselga J, (2017). Therapy-related clonal hematopoiesis in patients with non-hematologic cancers is common and associated with adverse clinical outcomes. Cell Stem Cell 21, 374–382.e4. 10.1016/j.stem.2017.07.010. 28803919 PMC5591073

[9] Fuster JJ, MacLauchlan S, Zuriaga MA, Polackal MN, Ostriker AC, Chakraborty R, Wu CL, Sano S, Muralidharan S, Rius C, (2017). Clonal hematopoiesis associated with TET2 deficiency accelerates atherosclerosis development in mice. Science 355, 842–847. 10.1126/science.aag1381. 28104796 PMC5542057

[10] Dorsheimer L, Assmus B, Rasper T, Ortmann CA, Ecke A, Abou-El-Ardat K, Schmid T, Brüne B, Wagner S, Serve H, (2019). Association of mutations contributing to clonal hematopoiesis with prognosis in chronic ischemic heart failure. JAMA Cardiol. 4, 25–33. 10.1001/jamacardio.2018.3965. 30566180 PMC6439691

[11] Jaiswal S, and Libby P (2020). Clonal haematopoiesis: connecting ageing and inflammation in cardiovascular disease. Nat. Rev. Cardiol. 17, 137–144. 10.1038/s41569-019-0247-5. 31406340 PMC9448847

[12] Dawoud AAZ, Gilbert RD, Tapper WJ, and Cross NCP (2022). Clonal myelopoiesis promotes adverse outcomes in chronic kidney disease. Leukemia 36, 507–515. 10.1038/s41375-021-01382-3. 34413458 PMC8807385

[13] Yu B, Roberts MB, Raffield LM, Zekavat SM, Nguyen NQH, Biggs ML, Brown MR, Griffin G, Desai P, Correa A, (2021). Supplemental association of clonal hematopoiesis with incident heart failure. J. Am. Coll. Cardiol. 78, 42–52. 10.1016/j.jacc.2021.04.085. 34210413 PMC8313294

[14] Wong WJ, Emdin C, Bick AG, Zekavat SM, Niroula A, Pirruccello JP, Dichtel L, Griffin G, Uddin MM, Gibson CJ, (2023). Clonal haematopoiesis and risk of chronic liver disease. Nature 616, 747–754. 10.1038/s41586-023-05857-4. 37046084 PMC10405350

[15] Moran-Crusio K, Reavie L, Shih A, Abdel-Wahab O, Ndiaye-Lobry D, Lobry C, Figueroa ME, Vasanthakumar A, Patel J, Zhao X, (2011). Tet2 loss leads to increased hematopoietic stem cell self-renewal and myeloid transformation. Cancer Cell 20, 11–24. 10.1016/j.ccr.2011.06.001. 21723200 PMC3194039

[16] Mas-Peiro S, Hoffmann J, Fichtlscherer S, Dorsheimer L, Rieger MA, Dimmeler S, Vasa-Nicotera M, and Zeiher AM (2020). Clonal haematopoiesis in patients with degenerative aortic valve stenosis undergoing transcatheter aortic valve implantation. Eur. Heart J. 41, 933–939. 10.1093/eurheartj/ehz591. 31504400 PMC7033916

[17] Buscarlet M, Provost S, Zada YF, Bourgoin V, Mollica L, Dubé M-P, and Busque L (2018). Lineage restriction analyses in CHIP indicate myeloid bias for TET2 and multipotent stem cell origin for DNMT3A. Blood 132, 277–280. 10.1182/blood-2018-01-829937. 29764839

[18] Loberg MA, Bell RK, Goodwin LO, Eudy E, Miles LA, SanMiguel JM, Young K, Bergstrom DE, Levine RL, Schneider RK, and Trowbridge JJ (2019). Sequentially inducible mouse models reveal that Npm1 mutation causes malignant transformation of Dnmt3a-mutant clonal hematopoiesis. Leukemia 33, 1635–1649. 10.1038/s41375-018-0368-6. 30692594 PMC6609470

[19] Russler-Germain DA, Spencer DH, Young MA, Lamprecht TL, Miller CA, Fulton R, Meyer MR, Erdmann-Gilmore P, Townsend RR, Wilson RK, and Ley TJ (2014). The R882H DNMT3A mutation associated with AML dominantly inhibits wild-type DNMT3A by blocking its ability to form active tetramers. Cancer Cell 25, 442–454. 10.1016/j.ccr.2014.02.010. 24656771 PMC4018976

[20] Kassebaum NJ, Bernabé E, Dahiya M, Bhandari B, Murray CJ, and Marcenes W (2014). Global burden of severe periodontitis in 1990–2010: a systematic review and meta-regression. J. Dent. Res. 93, 1045–1053. 10.1177/0022034514552491. 25261053 PMC4293771

[21] Peres MA, Macpherson LMD, Weyant RJ, Daly B, Venturelli R, Mathur MR, Listl S, Celeste RK, Guarnizo-Herreño CC, Kearns C, (2019). Oral diseases: a global public health challenge. Lancet 394, 249–260. 10.1016/S0140-6736(19)31146-8. 31327369

[22] Hajishengallis G (2015). Periodontitis: from microbial immune subversion to systemic inflammation. Nat. Rev. Immunol. 15, 30–44. 10.1038/nri3785. 25534621 PMC4276050

[23] Righolt AJ, Jevdjevic M, Marcenes W, and Listl S (2018). Global-, regional-, and country-level economic impacts of dental diseases in 2015. J. Dent. Res. 97, 501–507. 10.1177/0022034517750572. 29342371

[24] Genco RJ, and Sanz M (2020). Clinical and public health implications of periodontal and systemic diseases: an overview. Periodontol. 2000 83, 7–13. 10.1111/prd.12344. 32385880

[25] Potempa J, Mydel P, and Koziel J (2017). The case for periodontitis in the pathogenesis of rheumatoid arthritis. Nat. Rev. Rheumatol. 13, 606–620. 10.1038/nrrheum.2017.132. 28835673

[26] Hajishengallis G, and Chavakis T (2021). Local and systemic mechanisms linking periodontal disease and inflammatory comorbidities. Nat. Rev. Immunol. 21, 426–440. 10.1038/s41577-020-00488-6. 33510490 PMC7841384

[27] Smolen JS, Aletaha D, Barton A, Burmester GR, Emery P, Firestein GS, Kavanaugh A, McInnes IB, Solomon DH, Strand V, (2018). Rheumatoid arthritis. Nat. Rev. Dis. Primers 4, 18001. 10.1038/nrdp.2018.1. 29417936

[28] Hajishengallis G (2014). Aging and its impact on innate immunity and inflammation: implications for periodontitis. J. Oral Biosci. 56, 30–37. 10.1016/j.job.2013.09.001. 24707191 PMC3974203

[29] Ebersole JL, Graves CL, Gonzalez OA, Dawson D 3rd, Morford LA, Huja PE, Hartsfield JK Jr., Huja SS, Pandruvada S, and Wallet SM (2016). Aging, inflammation, immunity and periodontal disease. Periodontol. 2000 72, 54–75. 10.1111/prd.12135. 27501491

[30] Dorshkind K, Montecino-Rodriguez E, and Signer RAJ (2009). The ageing immune system: is it ever too old to become young again? Nat. Rev. Immunol. 9, 57–62. 10.1038/nri2471. 19104499

[31] Weyand CM, and Goronzy JJ (2016). Aging of the immune system. Mechanisms and therapeutic targets. Ann. Am. Thorac. Soc. 13, S422–S428. 10.1513/AnnalsATS.201602-095AW. 28005419 PMC5291468

[32] Chalan P, van den Berg A, Kroesen B-J, Brouwer L, and Boots A (2015). Rheumatoid arthritis, immunosenescence and the hallmarks of aging. Curr. Aging Sci. 8, 131–146. 10.2174/1874609808666150727110744. 26212057 PMC5388800

[33] Divaris K, Monda KL, North KE, Olshan AF, Reynolds LM, Hsueh WC, Lange EM, Moss K, Barros SP, Weyant RJ, (2013). Exploring the genetic basis of chronic periodontitis: a genome-wide association study. Hum. Mol. Genet. 22, 2312–2324. 10.1093/hmg/ddt065. 23459936 PMC3652417

[34] Naorungroj S, Slade GD, Divaris K, Heiss G, Offenbacher S, and Beck JD (2017). Racial differences in periodontal disease and 10-year self-reported tooth loss among late middle-aged and older adults: the dental ARIC study. J. Public Health Dent. 77, 372–382. 10.1111/jphd.12226. 28585323 PMC5718983

[35] Papapanou PN, Sanz M, Buduneli N, Dietrich T, Feres M, Fine DH, Flemmig TF, Garcia R, Giannobile WV, Graziani F, (2018). Periodontitis: consensus report of workgroup 2 of the 2017 World Workshop on the Classification of Periodontal and Peri-Implant Diseases and Conditions. J. Clin. Periodontol. 45, S162–S170. 10.1111/jcpe.12946. 29926490

[36] Kim PG, Niroula A, Shkolnik V, McConkey M, Lin AE, S1abicki M, Kemp JP, Bick A, Gibson CJ, Griffin G, (2021). Dnmt3a-mutated clonal hematopoiesis promotes osteoporosis. J. Exp. Med. 218, e20211872. 10.1084/jem.20211872. 34698806 PMC8552148

[37] Abplanalp WT, Cremer S, John D, Hoffmann J, Schuhmacher B, Merten M, Rieger MA, Vasa-Nicotera M, Zeiher AM, and Dimmeler S (2021). Clonal hematopoiesis-driver DNMT3A mutations alter immune cells in heart failure. Circ. Res. 128, 216–228. 10.1161/CIRCRESAHA.120.317104. 33155517

[38] Busque L, Patel JP, Figueroa ME, Vasanthakumar A, Provost S, Hamilou Z, Mollica L, Li J, Viale A, Heguy A, (2012). Recurrent somatic TET2 mutations in normal elderly individuals with clonal hematopoiesis. Nat. Genet. 44, 1179–1181. 10.1038/ng.2413. 23001125 PMC3483435

[39] Jaiswal S, Natarajan P, Silver AJ, Gibson CJ, Bick AG, Shvartz E, McConkey M, Gupta N, Gabriel S, Ardissino D, (2017). Clonal hematopoiesis and risk of atherosclerotic cardiovascular disease. N. Engl. J. Med. 377, 111–121. 10.1056/NEJMoa1701719. 28636844 PMC6717509

[40] Florez MA, Tran BT, Wathan TK, DeGregori J, Pietras EM, and King KY (2022). Clonal hematopoiesis: mutation-specific adaptation to environmental change. Cell Stem Cell 29, 882–904. 10.1016/j.stem.2022.05.006. 35659875 PMC9202417

[41] Eskan MA, Jotwani R, Abe T, Chmelar J, Lim JH, Liang S, Ciero PA, Krauss JL, Li F, Rauner M, (2012). The leukocyte integrin antagonist Del-1 inhibits IL-17-mediated inflammatory bone loss. Nat. Immunol. 13, 465–473. 10.1038/ni.2260. 22447028 PMC3330141

[42] Moutsopoulos NM, and Konkel JE (2018). Tissue-specific immunity at the oral mucosal barrier. Trends Immunol. 39, 276–287. 10.1016/j.it.2017.08.005. 28923364 PMC5843496

[43] Dutzan N, Kajikawa T, Abusleme L, Greenwell-Wild T, Zuazo CE, Ikeuchi T, Brenchley L, Abe T, Hurabielle C, Martin D, (2018). A dysbiotic microbiome triggers TH17 cells to mediate oral mucosal immunopathology in mice and humans. Sci. Transl. Med. 10, eaat0797. 10.1126/scitranslmed.aat0797. 30333238 PMC6330016

[44] Hajishengallis G, and Korostoff JM (2017). Revisiting the Page & Schroeder model: the good, the bad and the unknowns in the periodontal host response 40 years later. Periodontol. 2000 75, 116–151. 10.1111/prd.12181. PMC5539911 28758305

[45] Hasegawa T, Kikuta J, Sudo T, Matsuura Y, Matsui T, Simmons S, Ebina K, Hirao M, Okuzaki D, Yoshida Y, (2019). Identification of a novel arthritis-associated osteoclast precursor macrophage regulated by FoxM1. Nat. Immunol. 20, 1631–1643. 10.1038/s41590-019-0526-7. 31740799

[46] Hajishengallis G, and Chavakis T (2022). Mechanisms and therapeutic modulation of neutrophil-mediated inflammation. J. Dent. Res. 101, 1563–1571. 10.1177/00220345221107602. 35786033 PMC9703529

[47] Yuh DY, Maekawa T, Li X, Kajikawa T, Bdeir K, Chavakis T, and Hajishengallis G (2020). The secreted protein DEL-1 activates a b3 integrin-FAK-ERK1/2-RUNX2 pathway and promotes osteogenic differentiation and bone regeneration. J. Biol. Chem. 295, 7261–7273. 10.1074/jbc.RA120.013024. 32280065 PMC7247308

[48] Nagai K, Ideguchi H, Kajikawa T, Li X, Chavakis T, Cheng J, Messersmith PB, Heber-Katz E, and Hajishengallis G (2020). An injectable hydrogel-formulated inhibitor of prolyl-4-hydroxylase promotes T regulatory cell recruitment and enhances alveolar bone regeneration during resolution of experimental periodontitis. FASEB J. 34, 13726–13740. 10.1096/fj.202001248R. 32812255 PMC7722135

[49] Kourtzelis I, Li X, Mitroulis I, Grosser D, Kajikawa T, Wang B, Grzybek M, von Renesse J, Czogalla A, Troullinaki M, (2019). DEL-1 promotes macrophage efferocytosis and clearance of inflammation. Nat. Immunol. 20, 40–49. 10.1038/s41590-018-0249-1. 30455459 PMC6291356

[50] Huynh M-LN, Fadok VA, and Henson PM (2002). Phosphatidylserine-dependent ingestion of apoptotic cells promotes TGF-β1 secretion and the resolution of inflammation. J. Clin. Invest. 109, 41–50. 10.1172/JCI11638. 11781349 PMC150814

[51] Jin S, Guerrero-Juarez CF, Zhang L, Chang I, Ramos R, Kuan CH, Myung P, Plikus MV, and Nie Q (2021). Inference and analysis of cell-cell communication using CellChat. Nat. Commun. 12, 1088. 10.1038/s41467-021-21246-9. 33597522 PMC7889871

[52] Patel SP, and Raju PA (2013). Resistin in serum and gingival crevicular fluid as a marker of periodontal inflammation and its correlation with single-nucleotide polymorphism in human resistin gene at −420. Contemp. Clin. Dent. 4, 192–197. 10.4103/0976-237X.114878. 24015008 PMC3757881

[53] Gokhale NH, Acharya AB, Patil VS, Trivedi DJ, Setty S, and Thakur SL (2014). Resistin levels in gingival crevicular fluid of patients with chronic periodontitis and type 2 diabetes mellitus. J. Periodontol. 85, 610–617. 10.1902/jop.2013.130092. 23805816

[54] Reddy DSP, Vineetha V, Alekya D, Sameevulla MD, Reddy NR, and Babu DSM (2019). “Estimation of Midkine Levels in Gingival Crevicular Fluid and Serum in Periodontal Health, Disease and After Treatment” — A clinico biochemical study. J. Orofac. Sci. 11, 110–115. 10.4103/jofs.jofs_149_19.

[55] Sun Q, Zhang Z, and Ou Y (2019). A allele of ICAM-1 Rs5498 and VCAM-1 Rs3181092 is correlated with increased risk for periodontal disease. Open Life Sci. 14, 638–646. 10.1515/biol-2019-0072. 33817202 PMC7874761

[56] Takegahara N, Kim H, and Choi Y (2022). RANKL biology. Bone 159, 116353. 10.1016/j.bone.2022.116353. 35181574 PMC9035122

[57] Buhrmann C, Shayan P, Aggarwal BB, and Shakibaei M (2013). Evidence that TNF-beta (lymphotoxin alpha) can activate the inflammatory environment in human chondrocytes. Arthritis Res. Ther. 15, R202. 10.1186/ar4393. 24283517 PMC3979010

[58] Otero M, Lago R, Gomez R, Lago F, Dieguez C, Gómez-Reino JJ, and Gualillo O (2006). Changes in plasma levels of fat-derived hormones adiponectin, leptin, resistin and visfatin in patients with rheumatoid arthritis. Ann. Rheum. Dis. 65, 1198–1201. 10.1136/ard.2005.046540. 16414972 PMC1798289

[59] Szumilas K, Szumilas P, Słuczanowska-Głąbowska S, Zgutka K, and Pawlik A (2020). Role of adiponectin in the pathogenesis of rheumatoid arthritis. Int. J. Mol. Sci. 21, 8265. 10.3390/ijms21218265. 33158216 PMC7662687

[60] Narazaki M, Tanaka T, and Kishimoto T (2017). The role and therapeutic targeting of IL-6 in rheumatoid arthritis. Expert Rev. Clin. Immunol. 13, 535–551. 10.1080/1744666X.2017.1295850. 28494214

[61] Abplanalp WT, Schuhmacher B, Cremer S, Merten M, Shumliakivska M, Macinkovic I, Zeiher AM, John D, and Dimmeler S (2023). Cell-intrinsic effects of clonal hematopoiesis in heart failure. Nat. Cardiovasc. Res. 2, 819–834. 10.1038/s44161-023-00322-x. PMC11357996 39196061

[62] Uriarte SM, and Hajishengallis G (2023). Neutrophils in the periodontium: interactions with pathogens and roles in tissue homeostasis and inflammation. Immunol. Rev. 314, 93–110. 10.1111/imr.13152. 36271881 PMC10049968

[63] McInnes IB, and Schett G (2011). The pathogenesis of rheumatoid arthritis. N. Engl. J. Med. 365, 2205–2219. 10.1056/NEJMra1004965. 22150039

[64] Shin J, Maekawa T, Abe T, Hajishengallis E, Hosur K, Pyaram K, Mitroulis I, Chavakis T, and Hajishengallis G (2015). DEL-1 restrains osteoclastogenesis and inhibits inflammatory bone loss in nonhuman primates. Sci. Transl. Med. 7, 307ra155. 10.1126/sci-translmed.aac5380. PMC4593066 26424570

[65] Tsukasaki M, and Takayanagi H (2019). Osteoimmunology: evolving concepts in bone-immune interactions in health and disease. Nat. Rev. Immunol. 19, 626–642. 10.1038/s41577-019-0178-8. 31186549

[66] Chen Y, Wang H, Yang Q, Zhao W, Chen Y, Ni Q, Li W, Shi J, Zhang W, Li L, (2022). Single-cell RNA landscape of the osteoimmunology microenvironment in periodontitis. Theranostics 12, 1074–1096. 10.7150/thno.65694. 35154475 PMC8771561

[67] Zhou Y, Yang D, Yang Q, Lv X, Huang W, Zhou Z, Wang Y, Zhang Z, Yuan T, Ding X, (2020). Single-cell RNA landscape of intratumoral heterogeneity and immunosuppressive microenvironment in advanced osteosarcoma. Nat. Commun. 11, 6322. 10.1038/s41467-020-20059-6. 33303760 PMC7730477

[68] Schett G (2007). Cells of the synovium in rheumatoid arthritis. Osteoclasts. Arthritis Res. Ther. 9, 203. 10.1186/ar2110. 17316459 PMC1860063

[69] Charles JF, Hsu L-Y, Niemi EC, Weiss A, Aliprantis AO, and Nakamura MC (2012). Inflammatory arthritis increases mouse osteoclast precursors with myeloid suppressor function. J. Clin. Invest. 122, 4592–4605. 10.1172/JCI60920. 23114597 PMC3533532

[70] Lee Y, Awasthi A, Yosef N, Quintana FJ, Xiao S, Peters A, Wu C, Kleinewietfeld M, Kunder S, Hafler DA, (2012). Induction and molecular signature of pathogenic TH17 cells. Nat. Immunol. 13, 991–999. 10.1038/ni.2416. 22961052 PMC3459594

[71] Schaefer BC, Schaefer ML, Kappler JW, Marrack P, and Kedl RM (2001). Observation of antigen-dependent CD8+ T-cell/dendritic cell interactions in vivo. Cell. Immunol. 214, 110–122. 10.1006/cimm.2001.1895. 12088410

[72] Liu S, Lockhart JR, Fontenard S, Berlett M, and Ryan TM (2020). Mapping the chromosomal insertion site of the GFP transgene of UBC-GFP mice to the MHC locus. J. Immunol. 204, 1982–1987. 10.4049/jimmunol.1901338. 32122998

[73] Hajishengallis G (2014). Immunomicrobial pathogenesis of periodontitis: keystones, pathobionts, and host response. Trends Immunol. 35, 3–11. 10.1016/j.it.2013.09.001. 24269668 PMC3947349

[74] Li X, Colamatteo A, Kalafati L, Kajikawa T, Wang H, Lim J-H, Bdeir K, Chung K-J, Yu X, Fusco C, (2020). The DEL-1/b3 integrin axis promotes regulatory T cell responses during inflammation resolution. J. Clin. Invest. 130, 6261–6277. 10.1172/JCI137530. 32817592 PMC7685741

[75] Dai YJ, Wang YY, Huang JY, Xia L, Shi XD, Xu J, Lu J, Su XB, Yang Y, Zhang WN, (2017). Conditional knockin of Dnmt3a R878H initiates acute myeloid leukemia with mTOR pathway involvement. Proc. Natl. Acad. Sci. USA 114, 5237–5242. 10.1073/pnas.1703476114. 28461508 PMC5441829

[76] Jiang SJ, and Wang S (2015). Dual targeting of mTORC1 and mTORC2 by INK-128 potently inhibits human prostate cancer cell growth in vitro and in vivo. Tumour Biol. 36, 8177–8184. 10.1007/s13277-015-3536-6. 25990456

[77] Xu H, Pan Y, and Zhang N (2021). A breakthrough in liver regeneration for treatment of liver cancer. Cancer Biol. Med. 18, 631–634. 10.20892/j.issn.2095-3941.2021.0293. 35979856 PMC8330539

[78] Lamm N, Rogers S, and Cesare AJ (2019). The mTOR pathway: implications for DNA replication. Prog. Biophys. Mol. Biol. 147, 17–25. 10.1016/j.pbiomolbio.2019.04.002. 30991055

[79] Hecker JS, Hartmann L, Rivière J, Buck MC, van der Garde M, Rothenberg-Thurley M, Fischer L, Winter S, Ksienzyk B, Ziemann F, (2021). CHIP and hips: clonal hematopoiesis is common in patients undergoing hip arthroplasty and is associated with autoimmune disease. Blood 138, 1727–1732. 10.1182/blood.2020010163. 34139005

[80] Agrawal M, Niroula A, Cunin P, McConkey M, Shkolnik V, Kim PG, Wong WJ, Weeks LD, Lin AE, Miller PG, (2022). TET2-mutant clonal hematopoiesis and risk of gout. Blood 140, 1094–1103. 10.1182/blood.2022015384. 35714308 PMC9461470

[81] Tariq F, Alobaidi B, Xavier M, Mccorkindale M, Veltman J, Isaacs J, Pratt A, Anderson A, and Collin M (2020). Clonal haematopoiesis-associated somatic mutations in rheumatoid arthritis. Ann. Rheum. Dis. 79, 226.2–22226. 10.1136/annrheumdis-2020-eular.5568.

[82] Savola P, Lundgren S, Keränen MAI, Almusa H, Ellonen P, Leirisalo-Repo M, Kelkka T, and Mustjoki S (2018). Clonal hematopoiesis in patients with rheumatoid arthritis. Blood Cancer J. 8, 69. 10.1038/s41408-018-0107-2. 30061683 PMC6066480

[83] Shlush LI (2018). Age-related clonal hematopoiesis. Blood 131, 496–504. 10.1182/blood-2017-07-746453. 29141946

[84] Billings M, Holtfreter B, Papapanou PN, Mitnik GL, Kocher T, and Dye BA (2018). Age-dependent distribution of periodontitis in two countries: findings from NHANES 2009 to 2014 and SHIP-TREND 2008 to 2012. J. Clin. Periodontol. 45, S130–S148. 10.1111/jcpe.12944. 29926501

[85] MacNee W, Rabinovich RA, and Choudhury G (2014). Ageing and the border between health and disease. Eur. Respir. J. 44, 1332–1352. 10.1183/09031936.00134014. 25323246

[86] Zenobia C, and Hajishengallis G (2015). Basic biology and role of interleukin-17 in immunity and inflammation. Periodontol. 2000 69, 142–159. 10.1111/prd.12083. 26252407 PMC4530463

[87] Ampomah PB, Cai B, Sukka SR, Gerlach BD, Yurdagul A Jr., Wang X, Kuriakose G, Darville LNF, Sun Y, Sidoli S, (2022). Macrophages use apoptotic cell-derived methionine and DNMT3A during efferocytosis to promote tissue resolution. Nat. Metab. 4, 444–457. 10.1038/s42255-022-00551-7. 35361955 PMC9050866

[88] Newman H, Shih YV, and Varghese S (2021). Resolution of inflammation in bone regeneration: from understandings to therapeutic applications. Biomaterials 277, 121114. 10.1016/j.biomaterials.2021.121114. 34488119 PMC8545578

[89] Smith AM, LaValle TA, Shinawi M, Ramakrishnan SM, Abel HJ, Hill CA, Kirkland NM, Rettig MP, Helton NM, Heath SE, (2021). Functional and epigenetic phenotypes of humans and mice with DNMT3A Overgrowth Syndrome. Nat. Commun. 12, 4549. 10.1038/s41467-021-24800-7. 34315901 PMC8316576

[90] Fuster JJ, Zuriaga MA, Zorita V, MacLauchlan S, Polackal MN, Viana-Huete V, Ferrer-Pérez A, Matesanz N, Herrero-Cervera A, Sano S, (2020). TET2-loss-of-function-driven clonal hematopoiesis exacerbates experimental insulin resistance in aging and obesity. Cell Rep. 33, 108326. 10.1016/j.celrep.2020.108326. 33113366 PMC7856871

[91] Al-Zahrani MS, Kayal RA, and Bissada NF (2006). Periodontitis and cardiovascular disease: a review of shared risk factors and new findings supporting a causality hypothesis. Quintessence Int. 37, 11–18. 16429698

[92] Pietras E, Rabe J, Higa K, Chavez J, Mills T, Nemkov T, Nerlov C, Dinarello C, D’Alessandro A, and DeGregori J (2018). IL-1 drives clonal hematopoiesis via selective induction of growth arrest in normal HSC. Exp. Hematol. 64, 8265. 10.1016/j.exphem.2018.06.119.

[93] Hormaechea-Agulla D, Matatall KA, Le DT, Kain B, Long X, Kus P, Jaksik R, Challen GA, Kimmel M, and King KY (2021). Chronic infection drives Dnmt3a-loss-of-function clonal hematopoiesis via IFN-gamma signaling. Cell Stem Cell 28, 1428–1442.e6. 10.1016/j.stem.2021.03.002. 33743191 PMC8349829

[94] Caiado F, Kovtonyuk LV, Gonullu NG, Fullin J, Boettcher S, and Manz MG (2023). Aging drives Tet2+/− clonal hematopoiesis via IL-1 signaling. Blood 141, 886–903. 10.1182/blood.2022016835. 36379023 PMC10651783

[95] Selvarani R, Mohammed S, and Richardson A (2021). Effect of rapamycin on aging and age-related diseases-past and future. GeroScience 43, 1135–1158. 10.1007/s11357-020-00274-1. 33037985 PMC8190242

[96] An JY, Kerns KA, Ouellette A, Robinson L, Morris HD, Kaczorowski C, Park SI, Mekvanich T, Kang A, McLean JS, (2020). Rapamycin rejuvenates oral health in aging mice. eLife 9, e54318. 10.7554/eLife.54318. 32342860 PMC7220376

[97] Niedernhofer LJ, and Robbins PD (2018). Senotherapeutics for healthy ageing. Nat. Rev. Drug Discov. 17, 377. 10.1038/nrd.2018.44. 29651106

[98] Yura Y, Miura-Yura E, Katanasaka Y, Min KD, Chavkin N, Polizio AH, Ogawa H, Horitani K, Doviak H, Evans MA, (2021). The cancer therapy-related clonal hematopoiesis driver gene Ppm1d promotes inflammation and non-ischemic heart failure in mice. Circ. Res. 129, 684–698. 10.1161/CIRCRESAHA.121.319314. 34315245 PMC8409899

[99] Sano S, Wang Y, Yura Y, Sano M, Oshima K, Yang Y, Katanasaka Y, Min KD, Matsuura S, Ravid K, (2019). JAK2 (V617F)-Mediated clonal hematopoiesis accelerates pathological remodeling in murine heart failure. JACC Basic Transl. Sci. 4, 684–697. 10.1016/j.jacbts.2019.05.013. 31709318 PMC6834960

[100] Fidler TP, Xue C, Yalcinkaya M, Hardaway B, Abramowicz S, Xiao T, Liu W, Thomas DG, Hajebrahimi MA, Pircher J, (2021). The AIM2 inflammasome exacerbates atherosclerosis in clonal haematopoiesis. Nature 592, 296–301. 10.1038/s41586-021-03341-5. 33731931 PMC8038646

[101] Netea MG, Domínguez-Andrés J, Barreiro LB, Chavakis T, Divangahi M, Fuchs E, Joosten LAB, van der Meer JWM, Mhlanga MM, Mulder WJM, (2020). Defining trained immunity and its role in health and disease. Nat. Rev. Immunol. 20, 375–388. 10.1038/s41577-020-0285-6. 32132681 PMC7186935

[102] Kaufmann E, Sanz J, Dunn JL, Khan N, Mendonca LE, Pacis A, Tzelepis F, Pernet E, Dumaine A, Grenier JC, (2018). BCG educates hematopoietic stem cells to generate protective innate immunity against tuberculosis. Cell 172, 176–190.e19. 10.1016/j.cell.2017.12.031. 29328912

[103] Mitroulis I, Ruppova K, Wang B, Chen LS, Grzybek M, Grinenko T, Eugster A, Troullinaki M, Palladini A, Kourtzelis I, (2018). Modulation of myelopoiesis progenitors is an integral component of trained immunity. Cell 172, 147–161.e12. 10.1016/j.cell.2017.11.034. 29328910 PMC5766828

[104] Kalafati L, Kourtzelis I, Schulte-Schrepping J, Li X, Hatzioannou A, Grinenko T, Hagag E, Sinha A, Has C, Dietz S, (2020). Innate immune training of granulopoiesis promotes anti-tumor activity. Cell 183, 771–785.e12. 10.1016/j.cell.2020.09.058. 33125892 PMC7599076

[105] Moorlag SJCFM, Khan N, Novakovic B, Kaufmann E, Jansen T, van Crevel R, Divangahi M, and Netea MG (2020). b-Glucan Induces Protective Trained Immunity against Mycobacterium tuberculosis Infection: A Key Role for IL-1. Cell Rep. 31, 107634. 10.1016/j.celrep.2020.107634. 32433977 PMC7242907

[106] Li X, Wang H, Yu X, Saha G, Kalafati L, Ioannidis C, Mitroulis I, Netea MG, Chavakis T, and Hajishengallis G (2022). Maladaptive innate immune training of myelopoiesis links inflammatory comorbidities. Cell 185, 1709–1727.e18. 10.1016/j.cell.2022.03.043. 35483374 PMC9106933

[107] Mitroulis I, Hajishengallis G, and Chavakis T (2024). Bone marrow inflammatory memory in cardiometabolic disease and inflammatory comorbidities. Cardiovasc. Res. 119, 2801–2812. 10.1093/cvr/cvad003. 36655373 PMC10874275

[108] Hata M, Andriessen EMMA, Hata M, Diaz-Marin R, Fournier F, Crespo-Garcia S, Blot G, Juneau R, Pilon F, Dejda A, (2023). Past history of obesity triggers persistent epigenetic changes in innate immunity and exacerbates neuroinflammation. Science 379, 45–62. 10.1126/science.abj8894. 36603072

[109] Heyde A, Rohde D, McAlpine CS, Zhang S, Hoyer FF, Gerold JM, Cheek D, Iwamoto Y, Schloss MJ, Vandoorne K, (2021). Increased stem cell proliferation in atherosclerosis accelerates clonal hematopoiesis. Cell 184, 1348–1361.e22. 10.1016/j.cell.2021.01.049. 33636128 PMC8109274

[110] Zheng GXY, Terry JM, Belgrader P, Ryvkin P, Bent ZW, Wilson R, Ziraldo SB, Wheeler TD, McDermott GP, Zhu J, (2017). Massively parallel digital transcriptional profiling of single cells. Nat. Commun. 8, 14049. 10.1038/ncomms14049. 28091601 PMC5241818

[111] Stuart T, Butler A, Hoffman P, Hafemeister C, Papalexi E, Mauck WM 3rd, Hao Y, Stoeckius M, Smibert P, and Satija R (2019). Comprehensive integration of single-cell data. Cell 177, 1888–1902.e21. 10.1016/j.cell.2019.05.031. 31178118 PMC6687398

[112] Rezwani M, Pourfathollah AA, and Noorbakhsh F (2022). rbioapi: user-friendly R interface to biologic web services’ API. Bioinformatics 38, 2952–2953. 10.1093/bioinformatics/btac172. 35561170

[113] Szklarczyk D, Gable AL, Lyon D, Junge A, Wyder S, Huerta-Cepas J, Simonovic M, Doncheva NT, Morris JH, Bork P, (2019). STRING v11: protein–protein association networks with increased coverage, supporting functional discovery in genome-wide experimental datasets. Nucleic Acids Res. 47, D607–D613. 10.1093/nar/gky1131. 30476243 PMC6323986

[114] Jin S, Plikus MV, and Nie Q (2023). CellChat for systematic analysis of cell-cell communication from single-cell and spatially resolved transcriptomics. Preprint at bioRxiv. 10.1101/2023.11.05.565674. 39289562

[115] Atherosclerosis Risk in Communities Investigators (1989). The Atherosclerosis Risk in Communities (ARIC) Study: design and objectives. Am. J. Epidemiol. 129, 687–702. 2646917

[116] Beck JD, Elter JR, Heiss G, Couper D, Mauriello SM, and Offenbacher S (2001). Relationship of periodontal disease to carotid artery intima-media wall thickness: the Atherosclerosis Risk in Communities (ARIC) study. Arterioscler. Thromb. Vasc. Biol. 21, 1816–1822. 10.1161/hq1101.097803. 11701471

[117] Loe H, and Silness J (1963). Periodontal disease in pregnancy. I. Prevalence and severity. Acta Odontol. Scand. 21, 533–551. 10.3109/00016356309011240. 14121956

[118] Benjamin D, Sato T, Cibulskis K, Getz G, Stewart C, and Lichtenstein L (2019). Calling somatic SNVs and indels with Mutect2. Preprint at bioRxiv. 10.1101/861054.

[119] Wang K, Li M, and Hakonarson H (2010). ANNOVAR: functional annotation of genetic variants from high-throughput sequencing data. Nucleic Acids Res. 38, e164. 10.1093/nar/gkq603. 20601685 PMC2938201

[120] Uddin MDM, Nguyen NQH, Yu B, Brody JA, Pampana A, Nakao T, Fornage M, Bressler J, Sotoodehnia N, Weinstock JS, (2022). Clonal hematopoiesis of indeterminate potential, DNA methylation, and risk for coronary artery disease. Nat. Commun. 13, 5350. 10.1038/s41467-022-33093-3. 36097025 PMC9468335

[121] Kadowaki N, Antonenko S, and Liu Y-J (2001). Distinct CpG DNA and polyinosinic-polycytidylic acid double-stranded RNA, respectively, stimulate CD11c Type 2 dendritic cell precursors and CD11c dendritic cells to produce Type I IFN. J. Immunol. 166, 2291–2295. 10.4049/jimmunol.166.4.2291. 11160284

[122] Essers MAG, Offner S, Blanco-Bose WE, Waibler Z, Kalinke U, Duchosal MA, and Trumpp A (2009). IFNalpha activates dormant haematopoietic stem cells in vivo. Nature 458, 904–908. 10.1038/nature07815. 19212321

[123] Hajishengallis G (2023). Illuminating the oral microbiome and its host interactions: animal models of disease. FEMS Microbiol. Rev. 47, fuad018. 10.1093/femsre/fuad018. 37113021 PMC10198557

[124] Maekawa T, Abe T, Hajishengallis E, Hosur KB, DeAngelis RA, Ricklin D, Lambris JD, and Hajishengallis G (2014). Genetic and intervention studies implicating complement C3 as a major target for the treatment of periodontitis. J. Immunol. 192, 6020–6027. 10.4049/jimmunol.1400569. 24808362 PMC4078411

[125] Tsukasaki M, Komatsu N, Nagashima K, Nitta T, Pluemsakunthai W, Shukunami C, Iwakura Y, Nakashima T, Okamoto K, and Takayanagi H (2018). Host defense against oral microbiota by bone-damaging T cells. Nat. Commun. 9, 701. 10.1038/s41467-018-03147-6. 29453398 PMC5816021

[126] Jiao Y, Darzi Y, Tawaratsumida K, Marchesan JT, Hasegawa M, Moon H, Chen GY, Núñez G, Giannobile WV, Raes J, (2013). Induction of bone loss by pathobiont-mediated nod1 signaling in the oral cavity. Cell Host Microbe 13, 595–601. 10.1016/j.chom.2013.04.005. 23684310 PMC3721316

[127] Kitamoto S, Nagao-Kitamoto H, Jiao Y, Gillilland MG III, Hayashi A, Imai J, Sugihara K, Miyoshi M, Brazil JC, Kuffa P, (2020). The intermucosal connection between the mouth and gut in commensal pathobiont-driven colitis. Cell 182, 447–462.e14. 10.1016/j.cell.2020.05.048. 32758418 PMC7414097

[128] Hasturk H, Hajishengallis G, Forsyth Institute Center for Clinical and Translational Research staff, Lambris JD, Mastellos DC, and Yancopoulou D (2021). Phase IIa clinical trial of complement C3 inhibitor AMY-101 in adults with periodontal inflammation. J. Clin. Invest. 131, e152973. 10.1172/JCI152973. 34618684 PMC8631591

[129] Xiao E, Mattos M, Vieira GHA, Chen S, Corrêa JD, Wu Y, Albiero ML, Bittinger K, and Graves DT (2017). Diabetes enhances IL-17 expression and alters the oral microbiome to increase its pathogenicity. Cell Host Microbe 22, 120–128.e4. 10.1016/j.chom.2017.06.014. 28704648 PMC5701758

[130] Abe T, and Hajishengallis G (2013). Optimization of the ligature-induced periodontitis model in mice. J. Immunol. Meth. 394, 49–54. 10.1016/j.jim.2013.05.002. PMC3707981 23672778

[131] Khachigian LM (2006). Collagen antibody-induced arthritis. Nat. Protoc. 1, 2512–2516. 10.1038/nprot.2006.393. 17406499

[132] Wang H, Li X, Kajikawa T, Shin J, Lim JH, Kourtzelis I, Nagai K, Korostoff JM, Grossklaus S, Naumann R, (2021). Stromal cell-derived DEL-1 inhibits Tfh cell activation and inflammatory arthritis. J. Clin. Invest. 131, e150578. 10.1172/JCI150578. 34403362 PMC8483759

[133] Dutzan N, Abusleme L, Konkel JE, and Moutsopoulos NM (2016). Isolation, characterization and functional examination of the gingival immune cell network. J. Vis. Exp, 53736. 10.3791/53736. 26967370 PMC4828169

[134] Boivin G, Faget J, Ancey P-B, Gkasti A, Mussard J, Engblom C, Pfirschke C, Contat C, Pascual J, Vazquez J, (2020). Durable and controlled depletion of neutrophils in mice. Nat. Commun. 11, 2762. 10.1038/s41467-020-16596-9. 32488020 PMC7265525

[135] Garlet GP, Cardoso CR, Mariano FS, Claudino M, de Assis GF, Campanelli AP, Avila-Campos MJ, and Silva JS (2010). Regulatory T cells attenuate experimental periodontitis progression in mice. J. Clin. Periodontol. 37, 591–600. 10.1111/j.1600-051X.2010.01586.x. 20642629

[136] Coe D, Begom S, Addey C, White M, Dyson J, and Chai J-G (2010). Depletion of regulatory T cells by anti-GITR mAb as a novel mechanism for cancer immunotherapy. Cancer Immunol. Immunother. 59, 1367–1377. 10.1007/s00262-010-0866-5. 20480365 PMC11030908

[137] Wolf R, Mascia F, Dharamsi A, Howard OMZ, Cataisson C, Bliskovski V, Winston J, Feigenbaum L, Lichti U, Ruzicka T, (2010). Gene from a psoriasis susceptibility locus primes the skin for inflammation. Sci. Transl. Med. 2, 61ra90. 10.1126/sci-translmed.3001108. PMC6334290 21148126

[138] Takahashi N, Udagawa N, Tanaka S, and Suda T (2003). Generating murine osteoclasts from bone marrow. Methods Mol. Med. 80, 129–144. 10.1385/1-59259-366-6:129. 12728715

[139] Bouxsein ML, Boyd SK, Christiansen BA, Guldberg RE, Jepsen KJ, and Müller R (2010). Guidelines for assessment of bone microstructure in rodents using micro-computed tomography. J. Bone Miner. Res. 25, 1468–1486. 10.1002/jbmr.141. 20533309

[140] Hao Y, Hao S, Andersen-Nissen E, Mauck WM III, Zheng S, Butler A, Lee MJ, Wilk AJ, Darby C, Zager M, (2021). Integrated analysis of multimodal single-cell data. Cell 184, 3573–3587.e29. 10.1016/j.cell.2021.04.048. 34062119 PMC8238499

[141] Babicki S, Arndt D, Marcu A, Liang Y, Grant JR, Maciejewski A, and Wishart DS (2016). Heatmapper: web-enabled heat mapping for all. Nucleic Acids Res. 44, W147–W153. 10.1093/nar/gkw419. 27190236 PMC4987948

[142] Jühling F, Kretzmer H, Bernhart SH, Otto C, Stadler PF, and Hoffmann S (2016). metilene: Fast and sensitive calling of differentially methylated regions from bisulfite sequencing data. Genome Res. 26, 256–262. 10.1101/gr.196394.115. 26631489 PMC4728377

[143] Cuzick J (1985). A wilcoxon-type test for trend. Stat. Med. 4, 87–90. 10.1002/sim.4780040112. 3992076

[144] Williams R (2012). Using the margins command to estimate and interpret adjusted predictions and marginal effects. The. Stata Journal 12, 308–331. 10.1177/1536867X1201200209.

