# Supplementary material for: Clonal hematopoiesis driven by mutated DNMT3A promotes inflammatory bone loss
Source: Cell. Author manuscript; Available in PMC 2024 Jul 14. (PMC11246233; doi:10.1016/j.cell.2024.05.003)
Supplement: MMC1 [file NIHMS1993381-supplement-MMC1.pdf]

Cell, Volume *187*

Supplemental information

Clonal hematopoiesis driven by mutated DNMT3A promotes inflammatory bone loss

Hui Wang, Kimon Divaris, Bohu Pan, Xiaofei Li, Jong-Hyung Lim, Gundappa Saha, Marko Barovic, Danai Giannakou, Jonathan M. Korostoff, Yu Bing, Souvik Sen, Kevin Moss, Di Wu, James D. Beck, Christie M. Ballantyne, Pradeep Natarajan, Kari E. North, Mihai G. Netea, Triantafyllos Chavakis, and George Hajishengallis

Table S1. Prevalence of CHIP mutations according to variant allele frequency (VAF) among the Dental ARIC participants (*n*=4,946) – Related to Table 1

Participants with CHIP	>2% VAF carriers	>10% VAF carriers
	n (column %)	*n* (column %)
All	191 (100); 3.9% of sample	108 (100); 2.2% of sample
DNMT3A mutation carriers	118 (61.8)	72 (66.7)
TET2 mutation carriers	24 (12.6)	8 (7.4)
ASXL1 mutation carriers	17 (8.9)	16 (14.8)
JAK2 mutation carriers	2 (1.1)	1 (0.9)
Other *(PPM1D, SF3B1, SRSF2, TP53)*	30 (15.7)	11 (10.2)

**Table S2. Descriptive information of Dental ARIC study participants with clinical and CHIP data (*n*=4,946) – Related to Table 1**				
	CHIP carriers	CHIP non-carriers	*DNMT3A* mutation carriers	*DNMT3A* mutation non-carriers
	n (row %)	*n* (row %)	*n* (row %)	*n* (row %)
**CHIP:** Any	191 (3.9)	4,755 (96.1)	118 (2.4)	4,828 (97.6)
**Age:** years, mean [SD]	64.5 [5.4]	62.3 [5.6]	64.1 [5.6]	62.3 [5.6]
**Sex**				
male	86 (3.9)	2,143 (96.1)	65 (2.4)	2,652 (97.6)
female	105 (3.9)	2,612 (96.1)	53 (2.4)	2,176 (97.6)
**Race**				
European American	138 (3.6)	3,736 (96.4)	79 (2.0)	3,795 (98.0)
African American	53 (4.9)	1,019 (95.1)	39 (3.6)	1,033 (96.4)
**Education level**				
Basic	30 (4.4)	648 (95.6)	19 (2.8)	659 (97.2)
Intermediate	70 (3.3)	2,029 (96.7)	40 (1.9)	2,059 (98.1)
Advanced	90 (4.2)	2,075 (95.8)	59 (2.7)	2,106 (97.3)
**Current smoker**				
no	154 (3.6)	4,155 (96.4)	92 (2.1)	4,217 (97.9)
yes	35 (5.7)	582 (94.3)	25 (4.1)	592 (95.9)
**Diabetic**				
no	166 (3.9)	4,065 (96.1)	103 (2.4)	4,128 (97.6)
yes	23 (3.4)	656 (96.6)	14 (2.1)	665 (97.9)
**Body mass index (BMI)**				
<25.0 kg/m^2	58 (4.6)	1,191 (95.4)	38 (3.0)	1,211 (97.0)
25.0-29.9 kg/m^2	74 (3.7)	1,938 (96.3)	43 (2.1)	1,969 (97.9)
≥30.0 kg/m^2	59 (3.5)	1,615 (96.5)	37 (2.2)	1,637 (97.8)
**Episodic dental care**				
no	131 (3.7)	3,410 (96.3)	15 (0.4)	3,526 (99.6)
yes	59 (4.3)	1,320 (95.7)	9 (0.7)	1,370 (99.3)

**Table S3. Associations between periodontal disease diagnoses, clinical measures of periodontal disease according to DNMT3A mutation carrier status obtained from marginal effects estimation* - Related to Table 1**			
	crude model	sex adjusted	sex + age adjusted
	p.p. (95% CI)	p.p. (95% CI)	p.p. (95% CI)
**Periodontitis diagnosis** (reference: Stages I and II)			
Stage IV	13.0 (2.3, 23.7)	12.8 (2.1, 23.5)	10.8 (0.0, 21.5)
**Clinical attachment loss**			
(% of sites with CAL ≥3 mm)	5.4 (1.0, 9.8)	5.4 (1.1, 9.7)	4.9 (0.6, 9.2)
**Gingival inflammation**			
(% of sites with gingiva index ≥1)	8.8 (1.0, 16.5)	8.7 (1.0, 16.4)	9.2 (1.5, 17.0)
*Crude, sex-, and sex- and age-adjusted logistic and linear regression models and average marginal effects (percentage points, p.p.) and associated 95 confidence intervals (CI).			
