# Supplementary material for: Clonal hematopoiesis driven by mutated DNMT3A promotes inflammatory bone loss
Source: Cell. Author manuscript; Available in PMC 2024 Jul 14. (PMC11246233; doi:10.1016/j.cell.2024.05.003)
Supplement: 2 [file NIHMS1993381-supplement-2.pdf]

CellPress
OPEN ACCESS

Supplemental figures

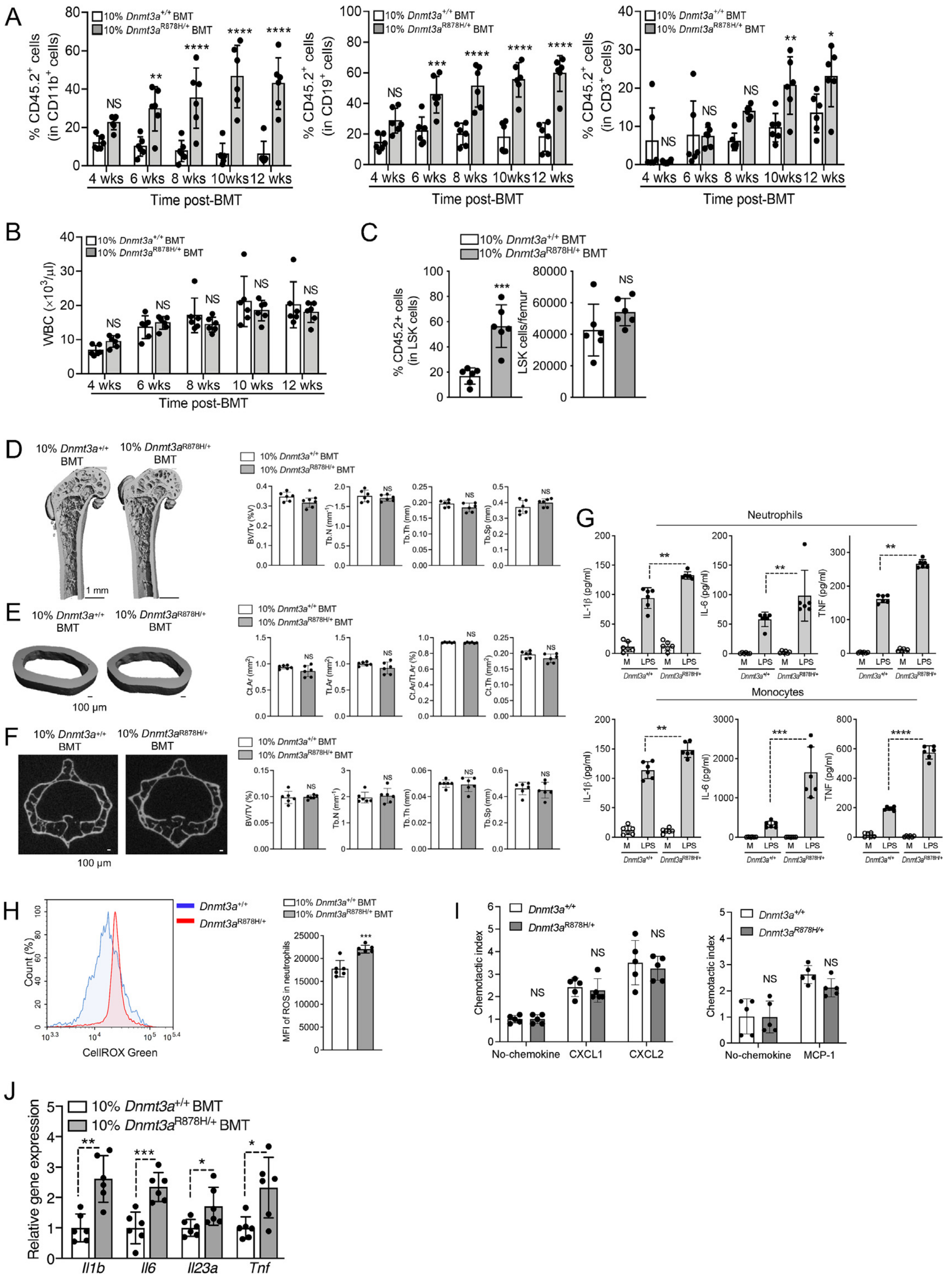

(legend on next page)

Figure S1. Clonal expansion of *Dnmt3a*$^{R878H/+}$ cells, their effects on skeletal bone, and inflammatory activities of the mutant myeloid cells, related to Figure 1

(A–F) Lethally irradiated CD45.1 mice were transplanted with either 10%*Dnmt3a*$^{R878H/+}$ CD45.2^{+} BM cells and 90% WT CD45.1^{+} BM cells (10% *Dnmt3a*$^{R878H/+}$BMT group) or with 10%*Dnmt3a*$^{+/+}$ CD45.2^{+} BM cells and 90% WT CD45.1^{+} cells (10%*Dnmt3a*$^{+/+}$BMT group). See Figure 1A.

(A) Percentage of CD45.2^{+} cells within the indicated white blood cell lineages at indicated time intervals.

(B) Total WBC cell counts in peripheral blood at indicated time intervals.

(C) Percentage of CD45.2^{+} cells within the Lin^{-}Sca1^{+}cKit^{+} (LSK) cell population (left) and total numbers of LSK cells (right).

(D) 3D microcomputed tomography (microCT) reconstruction of mouse femur bone from the two groups (left; scale bars, 1 mm) and microCT measurement of trabecular bone structural parameters in femur (right).

(E) 3D microCT reconstruction of the femur midshaft region (left; scale bars, 100 μm) and microCT measurement of the cortical bone structural parameters from the midshaft region (right).

(F) Cross-sectional images of lumbar vertebra (left; scale bars, 100 μm) and microCT measurement of trabecular bone structural parameters in lumbar vertebra (right).

BV/TV, bone volume fraction (ratio of trabecular bone volume to total bone volume); Tb.Th, trabecular thickness; Tb.N, trabecular number; Tb.Sp, trabecular separation; Ct.Ar, cortical bone area; Tt.Ar, total cross-sectional area; Ct.Ar/Tt.Ar, cortical area fraction; Ct.Th, cortical thickness.

(G) Cytokine levels in the supernatants of splenic neutrophils (Ly6G^{+}) and monocytes (Ly6G^{-}Ly6C^{+}), isolated from *Dnmt3a*$^{+/+}$ or *Dnmt3a*$^{R878H/+}$ mice, after 24-h incubation in the presence of 10 ng/mL LPS or medium-only control (M).

(H) Representative FACS histogram (left) and mean fluorescence intensity (MFI) of ROS in PMA-stimulated neutrophils from the peripheral blood of *Dnmt3a*$^{+/+}$ or *Dnmt3a*$^{R878H/+}$ mice.

(I) Chemotactic index of peripheral blood neutrophils (left) or splenic monocytes (right) from *Dnmt3a*$^{+/+}$ and *Dnmt3a*$^{R878H/+}$ mice. Chemotaxis toward CXCL1 or CXCL2 (neutrophils) or to MCP-1 (monocytes) was tested in a transwell system.

(J) Relative mRNA expression of indicated inflammatory cytokines in WT myeloid (CD45.1^{+}CD11b^{+}) cells isolated 12 weeks post-BMT from the gingiva of 10% *Dnmt3a*$^{R878H/+}$BMT or 10%*Dnmt3a*$^{+/+}$BMT mice (see experimental design in A–F).

Data are mean ± SD (A–F and J, $n = 6$ mice/group) or cell culture (G and H, $n = 6$; I, $n = 5$ replicates/group). *$p < 0.05$, **$p < 0.01$, ***$p < 0.001$; ****$p < 0.0001$; NS, not significant. Two-way ANOVA with repeated measures and Sidak's post-test (A and B); Student's unpaired t test (C–J) except for IL-1β and IL-6 in (G) top (Mann-Whitney U test).

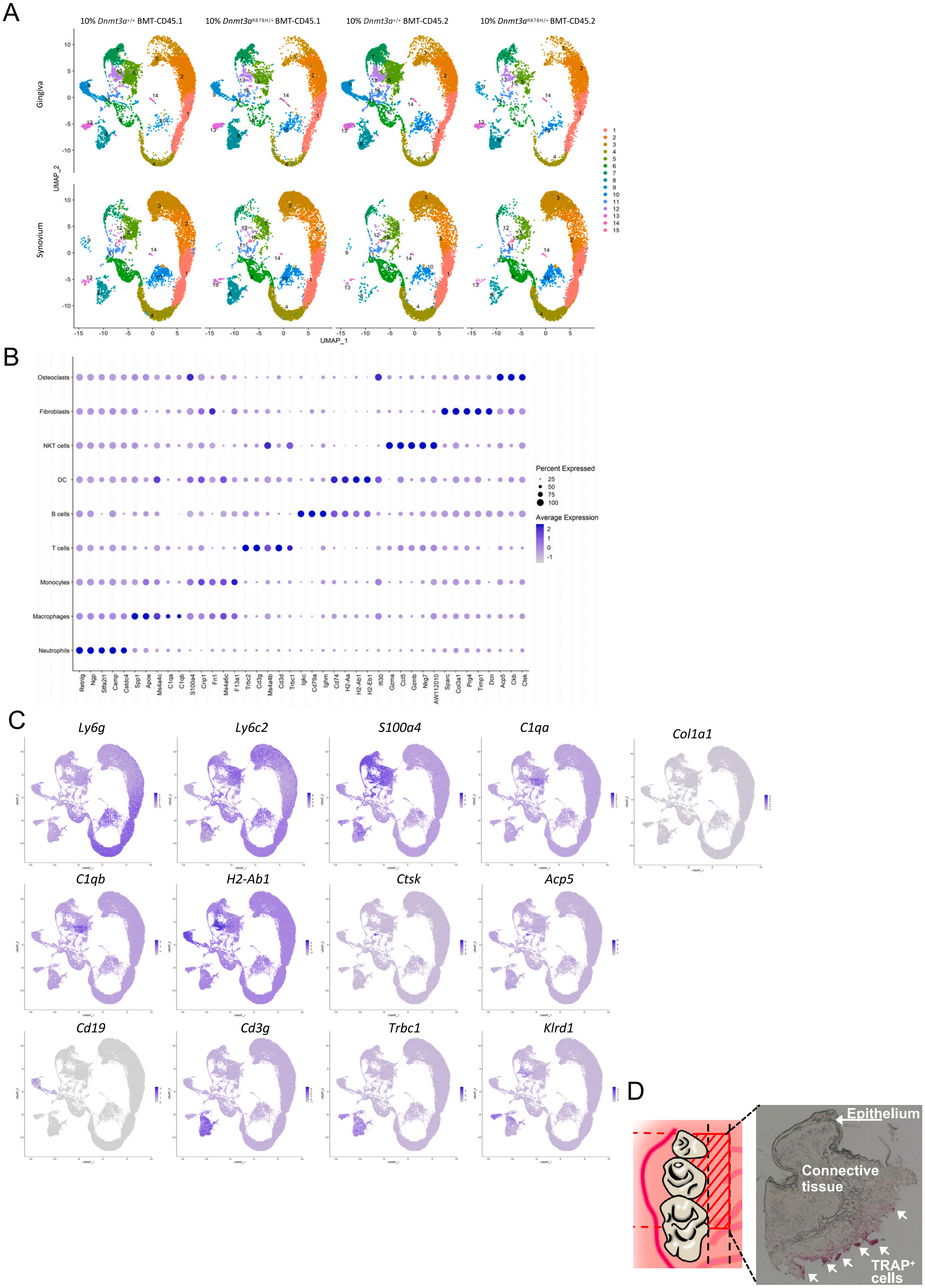

(legend on next page)

Figure S2. Two-dimensional UMAPs, top 5 marker genes, and specific makers for different cell types, related to Figure 4

(A–C) Lethally irradiated CD45.1 mice were transplanted with either 10%*Dnmt3a*$^{R878H/+}$ CD45.2 BM cells and 90% WT CD45.1 BM cells (10%*Dnmt3a*$^{R878H/+}$BMT group) or with 10%*Dnmt3a*$^{+/+}$ CD45.2 BM cells and 90% WT CD45.1 cells (10%*Dnmt3a*$^{+/+}$BMT group). After 12 weeks, both groups of recipient mice were subjected to LIP for 5 days or CAIA for 7 days, and dissected gingival and synovial tissues were processed for FACS of CD11b^{+} and CD3^{+} cells, which were further sorted into CD45.1^{+} and CD45.2^{+} cells, followed by scRNA-seq.

(A) Two-dimensional UMAP representation of 69,509 cells, according to sample origin and results of clustering.

(B) The expression of top 5 marker genes in distinctly defined cell types.

(C) Expression of specific makers to define different cell types. *Ly6c2* and *S100a4* define monocytes; *Ly6g*, neutrophils; *C1qa* and *C1qb*, macrophages; *H2-Ab1*, DC; *CD19*, B cells; *Acp5* and *Ctsk*, osteoclasts; *Col1a1*, fibroblasts; *CD3g* and *Trbc1*, T cells; and *Klrd1* and *Trbc1* define NKT cells.

(D) Dissected gingival tissue contains osteoclasts. Gingival tissues were dissected from LIP-subjected mice and stained with tartrate-resistant acid phosphatase (TRAP). TRAP^{+} cells (osteoclasts) can be observed in the areas overlying the alveolar bone.

CellPress
OPEN ACCESS

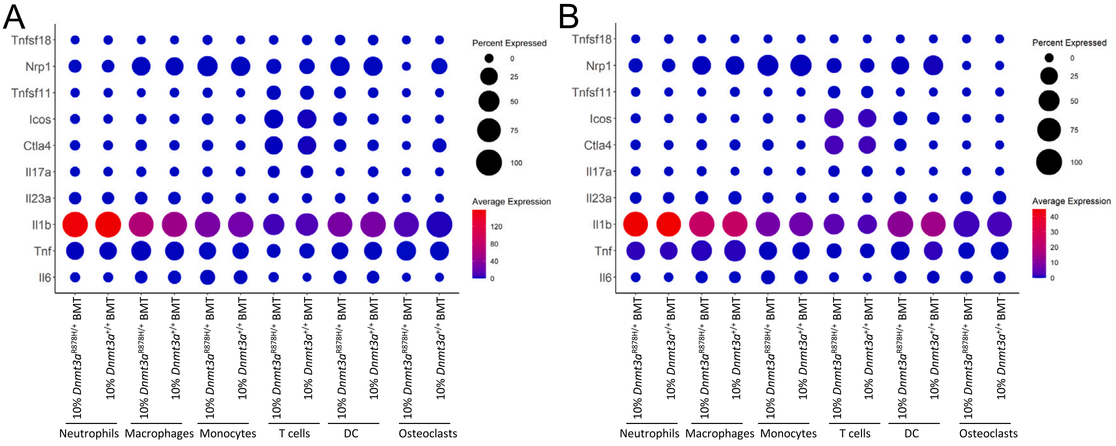

C

KEGG pathway ID	KEGG pathway description	Matching genes
mmu04514	Cell adhesion molecules	*Cd40, Cd4, Itgb8, H2-Aa, H2-Q7,H2-Eb1*
mmu04060	Cytokine-cytokine receptor interaction	*Ccl3, Cd40, Ccl4, Cd4, Tnf, Xcl1, Ifng*
mmu04064	NF-kappa B signaling pathway	*Cd40, Ccl4, Tnf, Bcl2a1b*
mmu04620	Toll-like receptor signaling pathway	*Ccl3, Cd40, Ccl4, Tnf*
mmu04659	Th17 cell differentiation	*Cd4, H2-Aa, Ifng, H2-Eb1*
mmu04218	Cellular senescence	*Rad50, Ccna2, H2-Q7, Cdk6*
mmu04350	TGF-beta signaling pathway	*Tnf, Ifng, Id1*

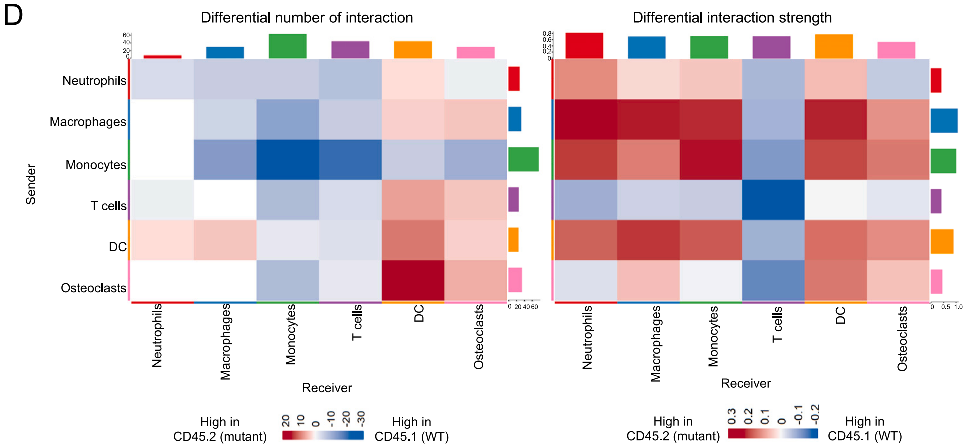

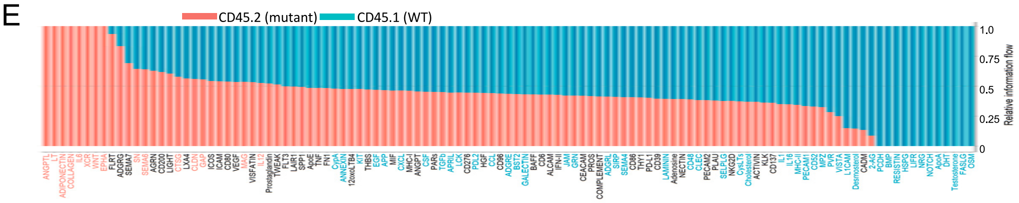

Figure S3. Expression of indicated genes in CD45.1^{+} cells, upregulated enriched genes in CD45.2^{+} cells in the indicated KEGG pathway terms and differential number and strength of possible interactions with overall information flow in synovium, related to Figure 4

Expression of indicated genes in distinctly defined CD45.1^{+} cell types (A and B), upregulated enriched genes in CD45.2^{+} cells in the indicated KEGG pathway terms (C) and differential number and strength of possible interactions with overall information flow in synovium (D and E). Lethally irradiated CD45.1 mice were transplanted with either 10%*Dnmt3a*$^{R878H/+}$ CD45.2 BM cells and 90% WT CD45.1 BM cells (10%*Dnmt3a*$^{R878H/+}$BMT group) or with 10%*Dnmt3a*$^{+/+}$ CD45.2 BM cells and 90% WT CD45.1 cells (10%*Dnmt3a*$^{+/+}$BMT group). After 12 weeks, both groups of recipient mice were subjected to LIP for 5 days or CAIA for 7 days, and dissected gingival and synovial tissues were processed for FACS of CD11b^{+} and CD3^{+} cells, which were further sorted into CD45.1^{+} and CD45.2^{+} cells, followed by scRNA-seq.

(A and B) Level of expression of indicated genes in distinctly defined CD45.1^{+} cell types from gingiva (A) or synovium (B).

(legend continued on next page)

(C) List of synovial upregulated enriched genes in CD45.2^{+} (*Dnmt3a*$^{R878H/+}$) cells in the indicated KEGG pathway terms analyzed by STRING.
(D and E) CellChat analysis of intercellular communication networks in CD45.2^{+} (mutant) and CD45.1^{+} (WT) cells from 10%*Dnmt3a*$^{R878H/+}$BMT mice.
(D) Heatmap of differential number and strength of possible interactions between any two of the indicated analyzed cell populations in synovium (red, increased interaction in mutant cells; blue, increased interaction in WT cells).
(E) Visualization of the overall information flow of each indicated signaling pathway by calculating the sum of communication probability among all pairs of synovial cell groups in the inferred network. The red and green colors indicate increased enrichment in mutant or WT cells, respectively.

CellPress
OPEN ACCESS

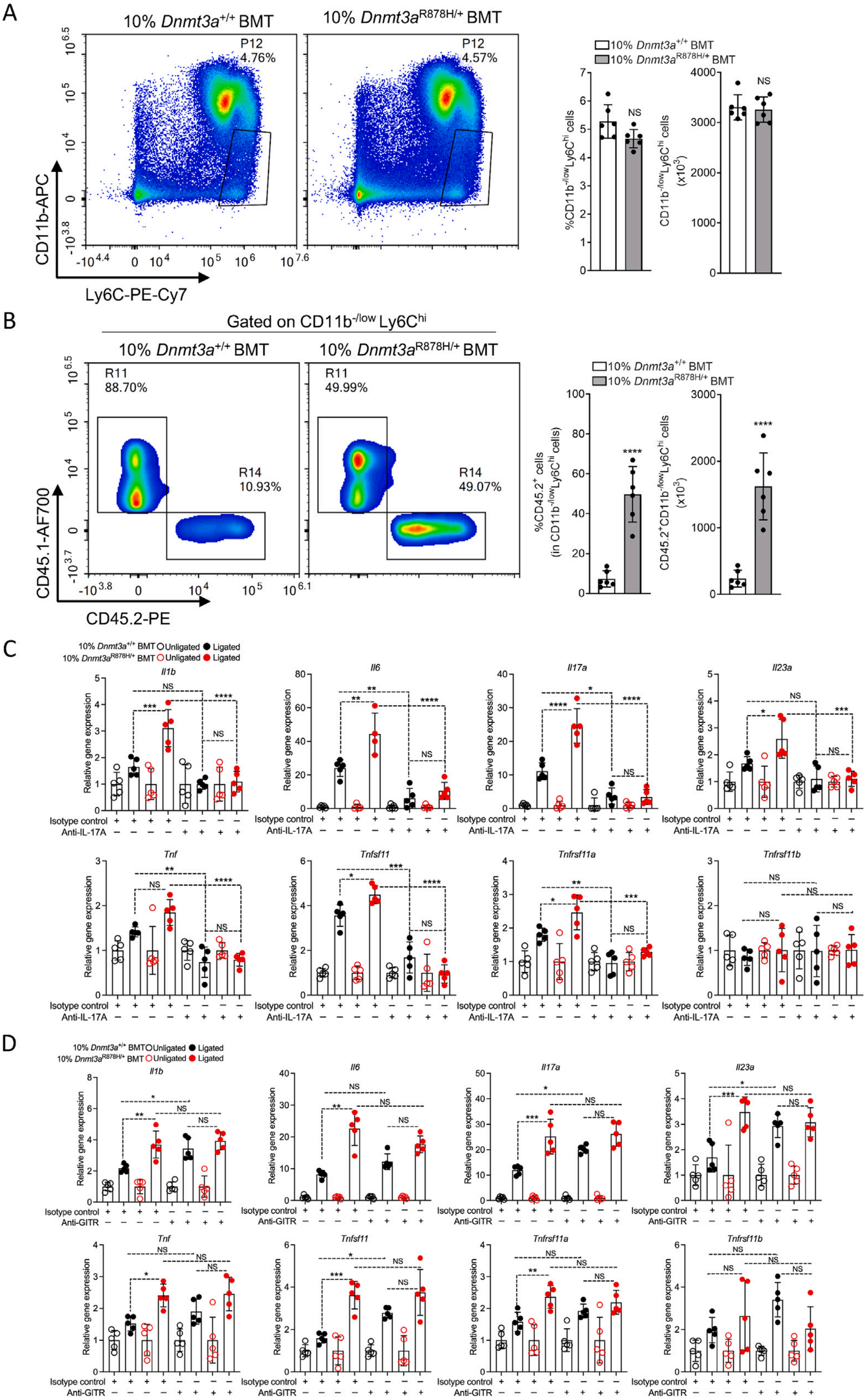

(legend on next page)

Figure S4. Clonal expansion of *Dnmt3a*$^{R878H/+}$ hematopoietic cells is not associated with increased numbers or frequency of CD11b$^{–/lo}$Ly6C^{hi} OCPs and effect of IL-17 neutralization and Treg depletion on inflammatory responses, related to Figure 5

Clonal expansion of *Dnmt3a*$^{R878H/+}$ hematopoietic cells is not associated with increased absolute numbers or frequency of CD11b$^{–/lo}$Ly6C^{hi} OCPs among BM cells (A and B) and effect of IL-17 neutralization and Treg depletion on inflammatory responses (C and D).

(A and B) Lethally irradiated CD45.1 mice were transplanted with either 10%*Dnmt3a*$^{R878H/+}$ CD45.2^{+} BM cells and 90% WT CD45.1^{+} BM cells (10% *Dnmt3a*$^{R878H/+}$BMT group) or with 10%*Dnmt3a*$^{+/+}$ CD45.2^{+} BM cells and 90% WT CD45.1^{+} cells (10%*Dnmt3a*$^{+/+}$BMT group). 12 weeks post-BMT, mice were subjected to LIP for 5 days, and femur BM cells were collected from the two groups for FACS analysis of osteoclast precursors.

(A) Representative FACS plots (left) and percentage within total BM cells (middle) and total numbers (right) of osteoclast precursors (OCPs), defined as CD11b$^{–/low}$ Ly6C^{hi}, in BM.

(B) Representative FACS plots (left) and percentage (middle) and total numbers (right) of CD45.2^{+} CD11b$^{-/low}$Ly6C^{hi} OCP in BM.

(C and D) BMT was performed as described above (A and B), and, after 12 weeks, recipient mice were subjected to LIP after local treatment with anti-IL-17A (or isotype control) (C) or after systemic treatment with anti-GITR (or isotype control) to deplete Tregs (D). Relative gingival mRNA expression of the indicated molecules after IL-17A neutralization (C) or after Treg cell depletion (D).

Data are mean ± SD (A and B, $n = 6$ mice/group; C, 4–5 mice/group; D, $n = 5$ mice/group). *$p < 0.05$, **$p < 0.01$, ***$p < 0.001$, ****$p < 0.0001$; NS, not significant. Student's unpaired t test (A and B); one-way ANOVA and Tukey's multiple comparisons test (C and D) except for (D; *Il6*) (Kruskal-Wallis test and Dunn's multiple comparisons test).

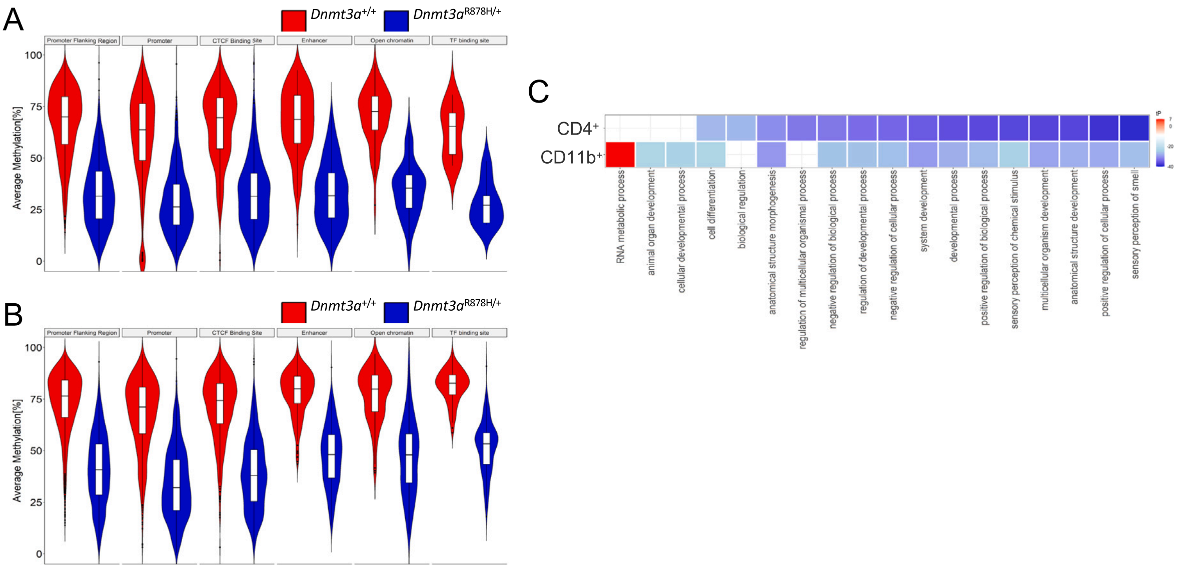

D

KEGG pathway ID	KEGG pathway description	Matching DMRs associated genes
mmu04010	MAPK signaling pathway	*Pdgfb, Akt1, Braf, Tgfb3, Ikbkg, Igf1r, Kit, Epha2, Srf, Mapkapk2, Map3k14, Fgf17, Rps6ka2, Map3k8, Map4k3, Pdgfrb, Rps6ka4, Efna1, Nfkb1, Map3k6, Flt1, Tnfrsf1a, Tradd, Mapkapk3, Gadd45a, Gng12, Ppp3ca, Prkca, Ptprr, Efna5, Map3k5, Mapt, Dusp16, Tek, Rps6ka1, Taok3, Cacna1c, Kdr, Csf1r, Nfatc1, Map4k4, Mef2c*
mmu04151	PI3K-Akt signaling pathway	*Pdgfb, Akt1, Ikbkg, Igf1r, Kit, Epha2, Ppp2cb, Ppp2r3c, Fgf17, Ywhaz, Ppp2r2b, Pdgfrb, Rptor, Itgav, Creb3l4, Efna1, Nfkb1, Flt1, Col1a2, Col4a1, Creb3l2, Itga9, Gng12, Pik3ap1, Fn1, Phlpp1, Pik3r1, Jak3, F2r, Prkca, Itgb5, Efna5, Ppp2r5c, Gng7, Tek, Pik3cd, Bcl2l11, Bcl2, Kdr, Csf1r, Sgk1, Ccnd3, Lpar5, Ppp2r1b, Creb5*
mmu04340	Hedgehog signaling pathway	*Smo, Ptch1, Gli2, Grk3, Smurf1, Sufu, Bcl2, Kif3a, Csnk1e, Cdon, Megf8*
mmu04380	Osteoclast differentiation	*Pparg, Akt1, Ikbkg, Fhl2, Map3k14, Nfkb1, Tnfrsf1a, Btk, Ppp3ca, Pik3r1, Nfatc2, Ncf4, Fyn, Pik3cd, Csf1r, Nfatc1, Stat1, Acp5*
mmu04659	Th17 cell differentiation	*Il12rb1, Ikbkg, Runx1, Nfkb1, Rora, Ppp3ca, Jak3, Rara, Nfatc2, Il17d, Stat6, Gata3, Nfatc1, Cd247, Stat1*
mmu04668	TNF signaling pathway	*Akt1, Ikbkg, Map3k14, Map3k8, Rps6ka4, Creb3l4, Nfkb1, Tnfrsf1a, Il15, Tradd, Creb3l2, Pik3r1, Map3k5, Pik3cd, Bcl3, Creb5*
mmu04660	T cell receptor signaling pathway	*Akt1, Ikbkg, Map3k14, Map3k8, Nfkb1, Grap2, Ppp3ca, Pik3r1, Nfatc2, Card11, Pak6, Fyn, Pik3cd, Nfatc1, Cd247*
mmu04350	TGF-beta signaling pathway	*Acvr1b, Tgfb3, Id3, Bmp7, Ppp2cb, Smad6, Smad1, Smurf1, Bmpr2, Acvr1, Thsd4, Tgif1, Ppp2r1b*
mmu04514	Cell adhesion molecules	*Cd274, Sdc4, Cldn7, Itgav, Jam3, F11r, Itga9, Cldn4, Cldn14, Cdh3, Pecam1, Mpzl1, Jam2, Cntnap2, Selp, H2-M5, H2-Q6, Ptprm*

E

KEGG pathway ID	KEGG pathway description	Matching DMRs associated genes
mmu04010	MAPK signaling pathway	*Gna12, Pdgfb, Akt1, Braf, Igf1r, Kit, Epha2, Srf, Hspa8, Mapkapk2, Grb2, Fgf17, Myc, Map3k8, Map4k3, Tnf, Map4k2, Rps6ka4, Fgf8, Efna1, Pdgfc, Nfkb1, Map3k6, Flt1, Vegfc, Dusp4, Tradd, Map2k5, Rasa2, Mapkapk3, Gadd45a, Ppp3ca, Prkca, Ptprr, Nf1, Efna5, Ppp3cc, Erbb3, Fgfr1, Map3k4, Map3k20, Relb, Map3k5, Mapt, Dusp16, Tek, Map3k1, Taok3, Cacna1s, Cacna1c, Kdr, Csf1r, Nlk, Tab2, Nfatc1, Map4k4, Mef2c, Hgf*
mmu04151	PI3K-Akt signaling pathway	*Ccnd2, Pdgfb, Akt1, Igf1r, Kit, Epha2, Ppp2cb, Grb2, Ppp2r3c, Fgf17, Myc, Ppp2r2b, Fgf8, Rptor, Lamc1, Itgav, Pik3ca, Creb3l4, Il6ra, Efna1, Pdgfc, Eif4e, Nfkb1, Flt1, Col1a2, Eif4ebp1, Vegfc, Creb3l2, Ppp2r2d, Itga9, Pik3ap1, Phlpp1, Pik3r1, Creb1, Jak3, Prkca, Pik3cg, Prkaa1, Jak2, Itgb5, Efna5, Rxra, Erbb3, Fgfr1, Ppp2r5c, Tek, Creb3, Foxo3, Pik3cd, Bcl2l11, Bcl2, Kdr, Csf1r, Sgk1, Cdk6, Ccnd3, Lpar5, Hgf, Creb5*
mmu04659	Th17 cell differentiation	*Il12rb1, Irf4, Runx1, Smad4, Il6ra, Nfkb1, Il21r, Rora, Smad3, Ppp3ca, Jak3, Jak2, Rara, Nfatc2, Rxra, Ppp3cc, Stat6, Gata3, Ahr, Stat3, Il21, Lck, Nfatc1, Il6st*
mmu04218	Cellular senescence	*Ccnd2, Akt1, Mapkapk2, Rad50, Myc, Pik3ca, Nfkb1, Itpr1, Eif4ebp1, Ets1, Smad3, Gadd45a, Itpr2, E2f2, Ppp3ca, Foxo1, Pik3r1, Capn2, Trpv4, Nfatc2, Fbxw11, Ppp3cc, E2f1, Foxo3, Pik3cd, Cdk6, Ccnd3, Nfatc1, H2-M5, H2-Q6*
mmu04668	TNF signaling pathway	*Akt1, Tnfaip3, Map3k8, Tnf, Rps6ka4, Jag1, Pik3ca, Creb3l4, Nfkb1, Vegfc, Il15, Tradd, Creb3l2, Pik3r1, Socs3, Creb1, Map3k5, Creb3, Pik3cd, Cflar, Bcl3, Tab2, Creb5*
mmu01100	Metabolic pathways	*Gmpr, Dbt, Agpat3, Ckb, Gnmt, Tbxas1, Man1a, Ggt1, Srm, Mvd, Aprt, B4galnt1, Pdhx, Cad, Amdhd1, Ada, Pip4k2b, Gpld1, Auh, Pcbd2, Cryl1, Tkt, Dpys, Sqle, Naprt, Umps, Impa2, Got1, Scd2, Hexa, Taldo1, Prps2, Atp6v1g3, Adcy10, Mgst3, Cat, Hsd17b12, Car2, Pik3ca, Npr1, Extl2, Gucy1b1, Atp6v0d2, Nans, B4galt1, Gne, St3gal3, Scp2, Ppt1, Azin2, Ak2, Extl1, Ugdh, Aldh2, Ldhb, Bcat1, Me3, Ebp, Prps1, Msmo1, Galnt7, Acsl1, St3gal2, Galnt2, St3gal4, Elovl5, St6galnac5, Sgpp2, Gmds, Pde9a, Mgat5, Fig4, B4galnt4, Galm, Ndufa4l2, Mpst, Ak7, Ndufb10, Pank1, Aldh4a1, Inpp5f, Bpgm, Chsy1, Adk, Pigv, Man1c1, Gsto2, Glb1, B3galnt1, Fkrp, Pgm1, Pik3cg, Phgdh, Aldh7a1, Chdh, B4galt6, Lclat1, Pygl, Bckdha, Cyp26c1, Ext1, Csgalnact1, Plcg2, Hoga1, Man2a1, Aldoa, Chpf2, Pde10a, Dgkg, Arsb, St6galnac6, Camkmt, Idh1, Mgst2, Pde11a, Dgki, Plcb2, Pik3cd, Gstm6, Pde4b, Anpep, Cox4i2, Plcb4, Sirt4, Sms, B3galt5, Entpd8, Gls, Bdh1, Synj2, Hk1, Ipmk, Acyp1, Synj1, Pde4d, Adcy9, Mgat4a, Entpd1, Alas1, Sgms1, Selenoi, Lpin2, Pde6h, Hal, Fhit, Mgat1, Mgll, Atp6v0e, Pafah1b2, Adcy4, Nt5c2, Hlcs, Hsd17b3, Gbe1, Gcnt1, Ddc, St6galnac3, Suclg2, Atp6v0e2, Pde2a, Pomgnt2, Inpp4b, Asah2, Ndst1, Gla*
mmu04380	Osteoclast differentiation	*Akt1, Spi1, Ncf1, Grb2, Tnf, Pik3ca, Nfkb1, Mitf, Ppp3ca, Pik3r1, Socs3, Creb1, Nfatc2, Ppp3cc, Plcg2, Relb, Fyn, Pik3cd, Csf1r, Tab2, Lck, Nfatc1*
mmu04062	Chemokine signaling pathway	*Akt1, Braf, Tiam1, Ncf1, Cxcl16, Grb2, Ptk2b, Gnaq, Pik3ca, Nfkb1, Shc4, Pik3r1, Jak3, Pik3cg, Jak2, Elmo1, Plcg2, Pxn, Src, Plcb2, Foxo3, Pik3cd, Plcb4, Ccr5, Adcy9, Stat3, Pard3, Adcy4*
mmu04660	T cell receptor signaling pathway	*Akt1, Grb2, Map3k8, Tnf, Pik3ca, Nfkb1, Ppp3ca, Pik3r1, Dlg1, Nfatc2, Ppp3cc, Card11, Nck2, Pak6, Fyn, Pik3cd, Lck, Nfatc1*
mmu04150	mTOR signaling pathway	*Akt1, Braf, Igf1r, Wnt10a, Wnt8a, Grb2, Tnf, Rptor, Atp6v1g3, Pik3ca, Eif4e, Eif4ebp1, Rragb, Pik3r1, Prkca, Prkaa1, Fzd5, Grb10, Pik3cd, Fzd7, Sgk1, Lpin2, Tbc1d7*

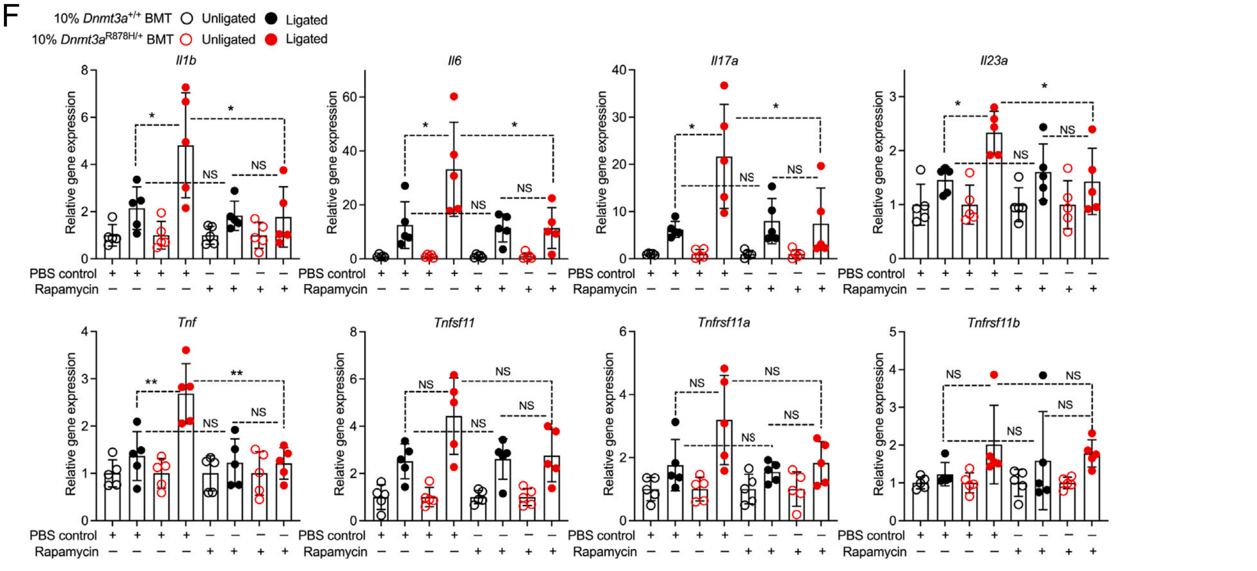

(legend on next page)

Figure S5. Whole-genome bisulfite sequencing of CD11b$^+$ and CD4$^+$ cells from *Dnmt3a*$^{R878H/+}$ mice and WT controls (A–E) and analysis of periodontal inflammation in rapamycin-treated '10%*Dnmt3a*$^{R878H/+}$BMT' mice (F) , related to Figure 6

Whole-genome bisulfite sequencing of CD11b$^+$ and CD4$^+$ cells from *Dnmt3a*$^{R878H/+}$ mice and WT controls (A–E) and analysis of periodontal inflammation in rapamycin-treated 10%*Dnmt3a*$^{R878H/+}$BMT mice (F).

(A–E) Splenic CD11b$^+$ and CD4$^+$ cells from *Dnmt3a*$^{+/+}$ and *Dnmt3a*$^{R878H/+}$ mice (n = 4 mice/group) were processed for WGBS.

(A and B) Average methylation values for CD11b$^+$ (A) and CD4$^+$ (B) cells from *Dnmt3a*$^{+/+}$ and *Dnmt3a*$^{R878H/+}$ mice in indicated regions of the genome. Hypothesis testing was performed via unpaired Student's t test within each genomic region ($p \leq 0.0001$).

(C) Top 15 significantly enriched GO terms sorted by PANTHER based on differentially methylated regions (DMRs) (in *Dnmt3a*$^{R878H/+}$ vs. *Dnmt3a*$^{+/+}$) in CD11b$^+$ and CD4$^+$ cells (Fisher test with FDR-correction, $p < 0.05$).

(D and E) List of hypomethylated genes in CD11b$^+$ (D) and CD4$^+$ (E) cells in the indicated KEGG pathway terms analyzed by STRING.

(F) BMT was performed as described in Figure 1A. The recipient 10%*Dnmt3a*$^{+/+}$BMT and 10%*Dnmt3a*$^{R878H/+}$BMT mice were treated with rapamycin or PBS control (see STAR Methods). 12 weeks post-BMT, all groups were subjected to LIP. Shown is relative gingival mRNA expression of the indicated molecules determined by quantitative real-time PCR. Data are mean ± SD (n = 5 mice/group). *$p < 0.05$, **$p < 0.01$. NS, not significant. One-way ANOVA with Tukey's multiple comparison test, except for *Tnfrsf11b* (Kruskal-Wallis test and Dunn's multiple comparisons test).
